# Transcriptome Analysis of iPSC-Derived Neurons from Rubinstein-Taybi Patients Reveals Deficits in Neuronal Differentiation

**DOI:** 10.1007/s12035-020-01983-6

**Published:** 2020-06-20

**Authors:** Luciano Calzari, Matteo Barcella, Valentina Alari, Daniele Braga, Rafael Muñoz-Viana, Cristina Barlassina, Palma Finelli, Cristina Gervasini, Angel Barco, Silvia Russo, Lidia Larizza

**Affiliations:** 1 Cytogenetics and Molecular Genetics Laboratory, Istituto Auxologico Italiano, IRCCS, Milan, Italy; 2 Genomic and Bioinformatics Unit, Department of Health Sciences, Università degli Studi di Milano, Milan, Italy; 3 Instituto de Neurociencias, Universidad Miguel Hernández - Consejo Superior de Investigaciones Científicas, Av. Santiago Ramón y Cajal s/n. Sant Joan d’Alacant, 03550 Alicante, Spain; 4 Department of Medical Biotechnology and Translational Medicine, Università degli Studi di Milano, Milan, Italy; 5 Medical Genetics, Department of Health Sciences, Università degli Studi di Milano, Milan, Italy

**Keywords:** Rubinstein Taybi, Intellectual disability, Neuronal differentiation, iPSC-derived neural progenitors, iNeurons, RNA-Seq, Defective transcriptional program

## Abstract

Rubinstein-Taybi syndrome (RSTS) is a rare multisystem developmental disorder with moderate to severe intellectual disability caused by heterozygous mutations of either *CREBBP* or *EP300* genes encoding CBP/p300 chromatin regulators. We explored the gene programs and processes underlying the morphological and functional alterations shown by iPSC-derived neurons modeling RSTS to bridge the molecular changes resulting from defective CBP/p300 to cognitive impairment. By global transcriptome analysis, we compared the differentially expressed genes (DEGs) marking the transition from iPSC-derived neural progenitors to cortical neurons (iNeurons) of five RSTS patients carrying private *CREBBP/EP300* mutations and manifesting differently graded neurocognitive signs with those of four healthy controls. Our data shows a defective and altered neuroprogenitor to neuron transcriptional program in the cells from RSTS patients. First, transcriptional regulation is weaker in RSTS as less genes than in controls are modulated, including genes of key processes of mature functional neurons, such as those for voltage-gated channels and neurotransmitters and their receptors. Second, regulation is subverted as genes acting at pre-terminal stages of neural differentiation in cell polarity and adhesive functions (members of the cadherin family) and axon extension/guidance (members of the semaphorins and SLIT receptors families) are improperly upregulated. Impairment or delay of RSTS neuronal differentiation program is also evidenced by decreased modulation of the overall number of neural differentiation markers, significantly impacting the initial and final stages of the differentiation cascade. Last, extensive downregulation of genes for RNA/DNA metabolic processes confirms that RSTS is a global transcription disorder, consistent with a syndrome driven by chromatin dysregulation. Interestingly, the morphological and functional alterations we have previously appointed as biomarkers of RSTS iNeurons provide functional support to the herein designed transcriptome profile pointing to key dysregulated neuronal genes as main contributors to patients’ cognitive deficit. The impact of RSTS transcriptome may go beyond RSTS as comparison of dysregulated genes across modeled neurodevelopmental disorders could unveil convergent genes of cognitive impairment.

**Electronic supplementary material:**

The online version of this article (10.1007/s12035-020-01983-6) contains supplementary material, which is available to authorized users.

## Introduction

Rubinstein-Taybi syndrome (RSTS1, MIM #180849, RSTS2, MIM #613684) is a multisystem developmental disorder affecting 1:125,000 newborns, characterized by moderate to severe intellectual disability (ID), growth delay, facial dysmorphisms, skeletal abnormalities, mainly of hands and feet, multi-organ malformations, and cancer predisposition [1]. It is caused by heterozygous mutations of either *CREBBP* (cAMP responding element-binding protein (CREB) binding protein) (MIM #600140) (60%) [2] or *EP300* (EIA-associated protein p300) (MIM #602700) (8–10%) [3, 4] genes which encode CBP and p300 homologous transcriptional co-activators with lysine acetyltransferase activity (KAT) acting as epigenetic regulators [5–9]. Besides locus heterogeneity, a pronounced allelic heterogeneity is attested by the mostly unique out of the 372 variants of the major *CREBBP* (https://databases.lovd.nl/shared/genes/CREBBP) and > 100 of the later identified *EP300* gene (https://databases.lovd.nl/shared/genes/EP300). The genetic heterogeneity is the main determinant of the broad RSTS1/RSTS2 phenotypic spectrum with intellectual disability, at times accompanied by behavior alterations, ranging from mild to severe across patients [10].

Generation and in-depth characterization of multiple CBP-deficient strains, including *Cbp+/−*, conditional knock-out (cKO) mice, and transgenic mice expressing a dominant negative allele, provided important clues to unravel the etiology of RSTS and to demonstrate the contribution of CBP/p300 to cognitive functions both during development and adult life [11–13]. CBP and p300 are required during development and contribute to the differentiation of diverse cell types, including different classes of neurons [14–16]. CBP has been also involved in neuronal maturation orchestrating the gene programs underlying neuronal outgrowth and activity-dependent synaptic maturation [17]. In addition, these proteins may also act as a nexus between the environment and transcriptional regulation at later stages of development and in the adult brain [8, 9]. The extensive delineation of the CBP/p300 interactome with > 400 binding proteins (of which ~ 100, mainly transcription factors and chromatin remodelers) implicated as acetylation substrates including enhancer-associated regulators [18] suggested that altered/defective CBP/p300 proteins impact a myriad of downstream targets [6]. However, since CBP/p300 acts in large protein complexes, it has not been possible to distinguish molecular targets of the intertwined KAT, scaffolding, and coactivator functions [8, 19]. Further complexity in deciphering molecular pathomechanisms is accounted for by the differential sensitivity of CBP/p300 effectors depending on the cellular context [20] and by the rapid dynamics of the CBP/p300 acetylome [19].

Despite extensive genomic characterization of RSTS individuals [2, 3], limitations due to the only use of patient-specific lymphoblastoid cell lines [21] have not enabled to decipher the cascade of events going awry in neurodevelopment upon mutation of *CREBBP*/*EP300* genes.

In order to discern the molecular mechanisms and biological processes responsible for the hallmark clinical sign of RSTS patients, i.e., intellectual disability, we took advantage of the iPSC-derived neuronal model generated using the non-integrating Sendai virus as described in [22–24]. iPSCs reprogrammed from blood of RSTS1 and RSTS2 patients successfully generated, likewise iPSCs from healthy individuals, neural progenitor cells (NPCs) which then differentiated into cortical neurons. However, morphological and functional alterations were shown by the young and mature RSTS neurons, respectively [24], raising the question of exploring the transcriptional dysregulation underlying these defects to understand the molecular basis of RSTS patients cognitive deficits.

iPSC-derived neurons (aka iNeurons) are a well-suited system to disclose disease mechanisms and identity and gene expression profiling of patients and control samples allowed to highlight gene processes disrupted in neurodevelopmental disorders (NDDs) [25] including idiopathic autism [26], CHD8 (chromodomain helicase DNA-binding protein 8)-caused autism [27], Rett syndrome [28, 29], Fragile X [30, 31], Prader-Willi/Angelman [32], and Kleefstra [33] syndromes. It has been claimed that human models for studying NDDs that result in intellectual disability are complementary to animal models, as they disclose disease mechanisms unique to humans and can bridge some of the gaps between animal phenotypes and human diseases [34]. Generation of an iPSC neuronal model is highly relevant to RSTS given that the only human model exploring the role of *CREBBP* mutations during neural differentiation is represented by embryonal carcinoma cells (NT2 cells) transfected with *CREBBP* deletion constructs [35]. In addition, Rubinstein-Taybi syndrome belongs to the rare monogenic NDDs resulting from defects of the epigenetic machinery [5, 7, 36, 37] including Rett syndrome, *CDH8*-caused autism, and Kleefstra syndrome, and cross-analysis of the studies performed on transcriptional networks in iNeurons of patients with these NDDs [27, 29, 33] might point to merging dysregulated biological pathways.

By high-throughput global transcriptome RNA sequencing (RNA-Seq), we examined the differentially expressed genes (DEGs) marking the transition from iPSC-derived NPCs to post-mitotic neurons from five RSTS patients carrying private *CREBBP*/*EP300* mutations and manifesting differently graded intellectual disability as compared to four healthy controls. Our data show that genes involved in functions critical for cortical development such as cell-to-cell adhesion and axonal guidance are improperly upregulated in RSTS iNeurons, while many genes which should be active at this and the final neurodifferentiation stage of synaptic integration are not regulated. A profile of extensive downregulation of nuclei acids metabolism genes, mainly safeguarding RNA processing and ribosome biogenesis, characterizes RSTS iNeurons. The overall data showcase the defective transcriptional program underlying the conversion of NPCs to iNeurons in RSTS cells.

## Results

### RSTS iPSC-Neuronal Lines Show a Gradient of Morpho-functional Alterations Mirroring the ID Spectrum of the Donor Patients

**Fig. 1:**
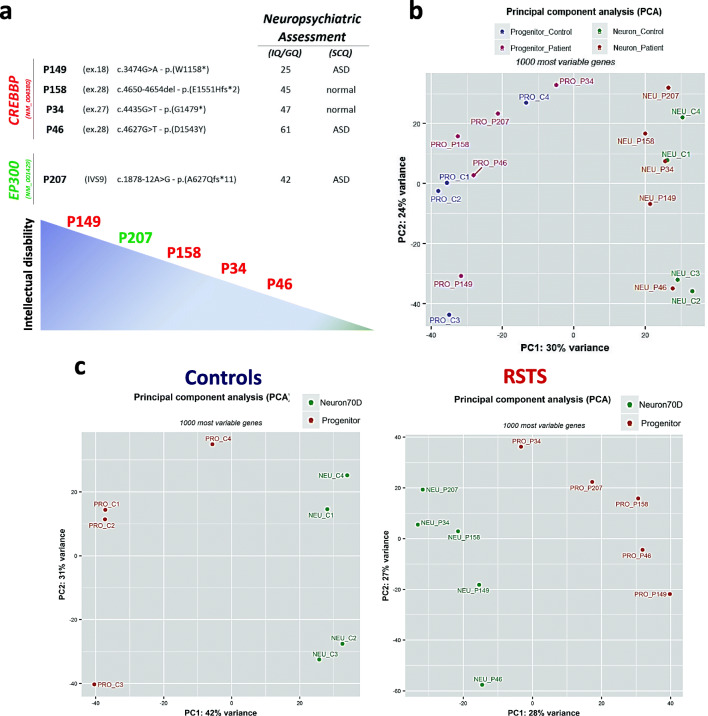
Exploratory data analysis. **a** Table showing RSTS patients selected for transcriptome analysis: individuals are ordered according to the degree of intellectual disability and presence/absence of behavior disorder assessed by different scales (IQ: intellectual quotient (Leiter R); GQ: general quotient of development (Griffith scales); SCQ: Social Communication Questionnaire). Affected loci (*CREBBP*/*EP300*), type of mutation, and predicted effect on protein are provided. **b**, **c** Principal component analysis (PCA) of gene expression data (1000 most variable genes) of all individuals (**b**), controls (**c** left panel), and RSTS patients (**c** right panel) at both differentiation stages (iNeurons vs NPCs). Only the first two major principal components are shown

We carried out transcriptome profiling of neural progenitor cells (NPCs) and neurons differentiated using the monolayer protocol [32] after 35 and 70 days, respectively, from the iPSC lines of 5 RSTS patients (4 *CREBBP*- and 1 *EP300*-mutated). The clinical characteristics of the donor patients and the characterization of their pathogenic variants have been reported [2, 4, 21, 38, 39] as well as the morphological and functional defects of their iPSC-derived neurons [24 and unpublished data]. As the cognitive phenotype is milder in RSTS2 than RSTS1 patients, out of the two in vitro modeled *EP300+/−* patients, we selected P207 who shows a moderate ID and autistic features. Indeed, as shown in Fig. 1a that ranks the iPSC donor patients according to the intellectual quotient (IQ)/general quotient of development (GQ) and behavioral aspects assessment, patient 207 is the second most severe. Out of the three *CREBBP+/−* patients carrying inactivating mutations, P149 displays the most severe ID and overt ASD signs, P158 and P34 exhibit a moderate ID, while P46, carrier of a missense mutation, has a slight ID, though accompanied by stereotypies and social interaction problems.

Successful differentiation to cortical neurons could be obtained in all patients, while differentiation efficiency, slightly variable between neuronal lines, was the lowest for P149, whose cells only in limited percentage expressed the neuronal stage-specific markers at the same time point of controls and showed the most altered morphological parameters and electric activity [24 and unpublished data]. Immunohistochemistry did not evidence relevant differences among patients and between patients and controls in the expression of differentiation markers at the stages of neural rosettes characterized by positivity for the neuroectodermal markers PAX6 and NESTIN and post-mitotic (> 70 days) neurons positive for the neuronal marker TUJ1 (beta III tubulin) and the cortical marker CUX-1. Conversely, at the stage of early (42 days) neurons, positive for the pan-neuronal markers MAP2 (microtubule-associated protein 2) and TUJ1, the low cell density permits to detect differences in the neuronal layout between control and patient samples, thus offering an ideal time point for tracking the morphological parameters of differentiating neurons. These generalities can be appreciated in the Additional File 1 which provides the immunohistochemical and morphological characterization of iPSC-derived neural rosettes, young and mature neurons from one control and three patients, P46, P34, and P149 ranked according to increasing ID with P34 in intermediate position also representing the nearby P158 and P207. No significant differences are observed between samples at the neural rosette and the mature neurons stage, while the morphological alterations of early neurons are increasingly apparent from P46 to P149. The layout of early neurons likewise the e-recordings of mature neurons from the same patients [24 and unpublished data] hence provides cellular biomarkers of patients’ cognitive impairment.

Thus, the 5 neuronal cultures composing the set for transcriptome analysis fairly represent the variable cognitive impairment of RSTS patients.

### Differential Expression Analysis in iPSC-Derived Neural Progenitors and Cortical Neurons from RSTS and Healthy Controls

Total RNA libraries were sequenced in two batches producing 29.2 M ± 2.11 and 29.1 M ± 3.02 M read pairs on average for RSTS patients and controls, respectively. Sequencing quality of all samples resulted adequate in terms of percentages of reads mapping to exons yielding on average 23.47 ± 1.49 and 23.9 ± 2.38 millions of uniquely and unambiguously mapped fragments for patients and controls. Examination of the RNA-Seq reads mapped to the *CREBBP* and *EP300* exons confirmed the patients mutations, indicating that both the wild type and the mutated copies were detected in both NPCs and iNeurons (Additional File 2) although in different and variable proportions.

**Fig. 2:**
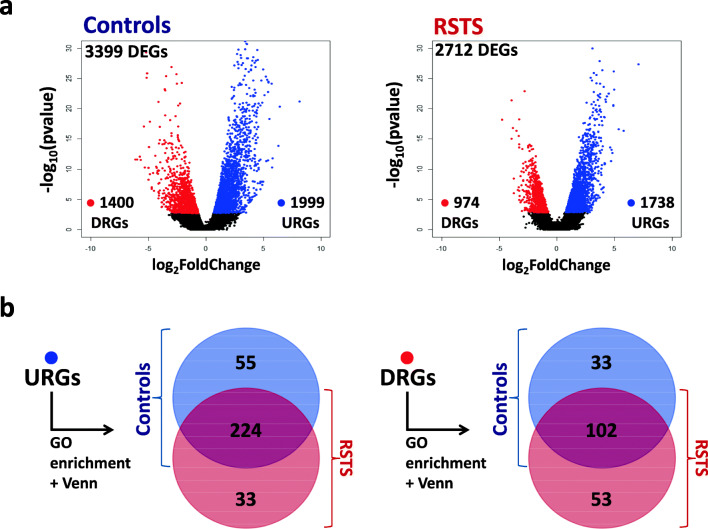
Gene expression changes in the transition from neural progenitors to mature neurons in controls and RSTS patients. **a** Volcano plot representation of differentially expressed genes (DEGs) in controls (left panel) and patients (right panel) in the transition from NPCs (35 days) to iNeurons (70 days). Red and blue points mark genes with significantly (FDR ≤ 0.01) upregulated genes (URGs) and downregulated genes (DRGs), respectively. No cutoff based on LogFoldChange (LFC) was applied. **b** Venn diagrams showing shared and univocal biological processes between RSTS and controls obtained from enrichment analysis of URGs (left panel) and DRGs (right panel) lists, respectively

Explorative data analysis involved principal component analysis (PCA) as primary tool to figure out samples/groups variability: PCA did not show a distinctive separation between RSTS patients and controls at both the NPCs and post-mitotic neurons time points (Fig. 1b and c) but showed a visible clustering of iNeurons from NPCs, which validates the expression program of differentiation to neurons (Fig. 1b and c). In keeping with our preliminary evaluations, we could not identify statistically significant differentially expressed genes (DEGs) (FDR < 0.01) between RSTS patients and controls either at NPCs or post-mitotic iNeurons time points. However, when we explored gene expression changes over time, from NPCs to mature neurons in RSTS and controls groups separately, we found that the total number of modulated genes was lower in RSTS than in controls with a more pronounced decrease of downregulated genes (DRGs) than upregulated genes (URGs) (31% versus 14%) (volcano plots in Fig. 2a and unsupervised heatmaps in Additional File 3). This result suggests that the neuronal identity is dented in RSTS cells, thus driving an impaired or leaky differentiation process.

### Over-Representation Analysis

We first analyzed the two cohorts separately by prioritizing total URGs and DRGs with a Gene Ontology (GO) analysis as unbiased method for identifying modulated/active/switched off biological processes (BP) during neural cell development: this analysis highlighted hundreds of DEGs-enriched BPs in controls and RSTS patients (Fig. 2b and Additional Files 4 and 5) which were compared by Venn analysis to sort common and univocal GO terms.

As can be seen in Fig. 2b, the shared BP lists are quite large (224 URGs- and 102 DRGs-enriched GO terms). To get insight into these lists, we used ReviGO (Reduce+Visualize Gene Ontology) tool [40] to cluster similar GO terms as treemaps (Additional Files 6 (from URGs) and 7 (from DRGs)). As expected, shared GO terms enriched in URGs (Additional File 6 - panel a) mainly involve processes which are switched on during differentiation of NPCs to cortical neurons such as synapsis organization and signaling, regulation of localization to synapse, and transport of proteins, neurotransmitters, and ions. As regards BP assigned to the alternative category of molecular functions (MF), terms aggregate homogeneously into functions encompassing inorganic transmembrane transport activities as ions or solute uptake that contribute to regulation of membrane potential transmission (Additional File 6 - panel b). On the other hand, the majority of shared GO terms enriched in DRGs highlights biological processes appropriately switched off in post-mitotic neurons such as DNA biosynthesis and metabolism, chromatin remodeling, and microtubule cytoskeleton organization (Additional File 7).

In order to identify as first step the transcriptional programs specific to RSTS patients and controls, “univocal” GO terms were investigated (Fig. 2b and Additional Files 4 and 5). Most of control univocal GO terms are related to processes of structural (dendrite) and functional organization (synapses) during neural development, while these processes are almost lacking in RSTS where conversely processes of negative neuronal regulation prevail. As to DRGs, both controls and RSTS show similar cellular and molecular processes converging to the arrest of cell proliferation.

**Fig. 3:**
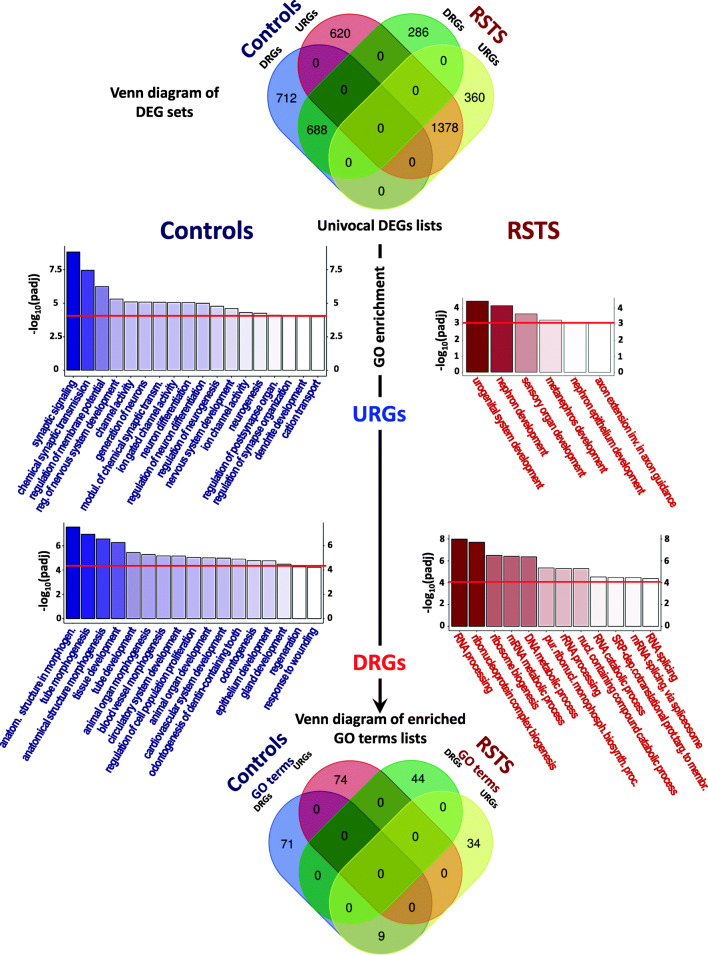
Differences of expression profiles of RSTS and healthy controls by analyzing univocal DEGs and enriched GO terms. Top panel: Venn diagram showing shared and univocal DEGs of RSTS and controls in the transition from NPCs (35 days) to iNeurons (70 days). Analysis highlighted four sets of DEGs not shared among the two groups (controls: 620 URGs and 712 DRGs; RSTS: 360 URGs and 286 DRGs) besides two large lists of “shared” genes, including 1378 URGs and 688 DRGs. Middle panels: bar plots representing most significant (padj<1 × 10^−4^; padj<1 × 10^−3^) GO terms enriched in univocal DEGs (URGs and DRGs) in controls (left side) and RSTS patients (right side). Enrichment of controls highlighted a total of 74 (up) and 80 (down) biological processes respectively, while in RSTS 43 (up) and 44 (down) enriched GO terms. Bottom panel: Venn diagram displaying the extent of overlap of enriched biological processes obtained from univocal lists of DEGs of RSTS and controls

Due to the remarkable redundancy and similarity of GO terms hampering the dissection of their control or RSTS specificity, we directly searched for differences in controls/RSTS DEGs lists, which were combined to identify univocal DEGs (Venn diagram in Fig. 3 top panel). To get insights on their key components, we performed a second step of GO enrichment. A selection of top GO terms (padj cutoff ranging from 1 × 10^−4^ and 1 × 10^−5^) from controls and RSTS enriched URGs and DRGs is shown in Fig. 3 (middle panels) while the 4 complete GO term lists from univocal URGs and from univocal DRGs are provided in Additional Files 8 and 9. Venn analysis confirmed the uniqueness of the enriched GO term lists as attested by the minimal overlap between control and RSTS GO terms (Fig. 3 bottom panel) indicating that the genes exclusively modulated in controls and RSTS iNeurons impact different biological processes. Overlap was only restricted to a few GO terms (9) with an opposite modulation.

**Fig. 4:**
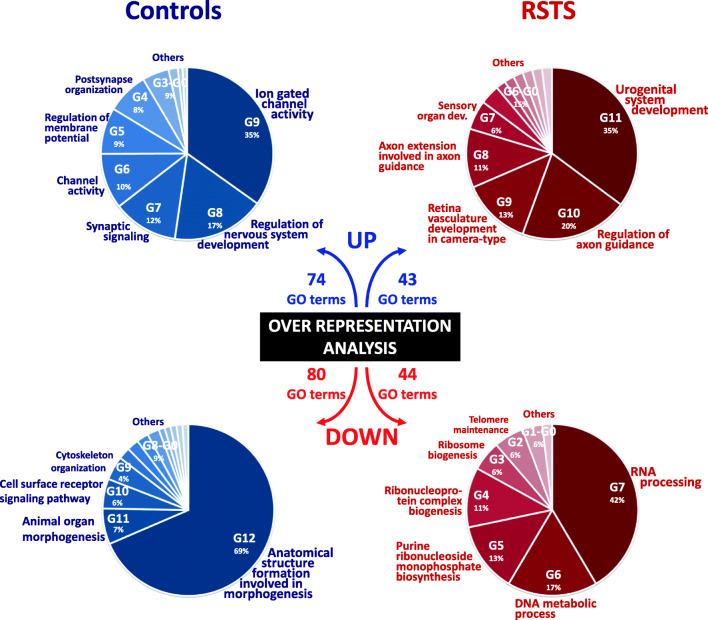
Pie charts of GO terms clusters in RSTS and controls. Pie charts showing GO terms groups obtained from analysis of univocal lists of URGs and DRGs in controls (left) and RSTS patients (right). The name of each cluster is reported near the corresponding slice. The order of groups and the extension of each slice is proportional to the percentage of GO terms

Next, aiming at summarizing biological processes, enriched terms were functionally grouped in clusters: pie charts of UP and DOWN clusters of controls and RSTS are depicted in Fig. 4 (the group hierarchy is set according to the relative percentage of GO terms). The complete lists of GO terms clusters with associated DEGs are provided in Additional Files 10 and 11. Sorting for GO terms (from univocal URGs) of controls and RSTS yielded two different lists of functional clusters (Fig. 4 top). Controls “UP” clusters summarize BP associated to physiological neuronal activities (e.g., regulation of nervous system development (G8), ion-gated channels activities (G9 and G6), membrane potentials (G5), and synaptic organization/signaling (G7 and G4) that are at least partially lacking in RSTS iNeurons. On the contrary, RSTS “UP” clusters depict BP “regulation of axon guidance” (G10) and “axon extension involved in axon guidance” (G8), significantly enriched in genes that, unlike controls, are upregulated.

Also the classification of downregulated GO terms produced two different lists of clusters (Fig. 4 bottom). Not surprisingly, the “DOWN” clusters from controls GO terms pointed to organ and tissue morphogenesis (G12 and G11) and system/organ/tissue/cell development (G9 and others), i.e., biological processes switched off upon neuronal differentiation. Enrichment from the list of downregulated RSTS GO terms highlighted clusters pointing to basic molecular processes of nucleic acids, in particular RNA, with G7 (RNA processing) associating the highest GO terms presence (42%).

### From GO Term Clusters to Top DEGs in RSTS iNeurons

We then focused our attention on the univocal most significant DEGs associated to the emerged macro-categories in RSTS neurons. By sorting the DEGs with a more stringent cutoff (padj < 10–3) according to GO term groups, we assembled the two *p* value ordered lists of top URGs/DRGs also providing log fold changes (LFC) and annotations (Tables 1 and 2 and Additional Files 10 and 11). To note that several significant DEGs pertain to more than one single group, meaning they contribute to enrich multiple, often related, GO terms: these “multifunctional” DEGs are asterisked. Controls univocal DEGs sorted into clusters are listed in Additional Files 10 and 11.

**Table 1:** RSTS top univocal URGs sorted into GO terms clusters

	Gene	padj (FDR)	LFC	Annotation
G11: urogenital system development				
	*AGT*	1.1E−08	3.14	Angiotensinogen
	*FAT4*	4.6E−07	1.84	FAT atypical cadherin 4
	*PDGFRA*	2.6E−06	2.40	Platelet-derived growth factor receptor alpha
	*NFIA*	5.3E−06	1.86	Nuclear factor I A
	*SALL1*	1.1E−05	1.67	Spalt-like transcription factor 1
	*SMAD9*	1.5E−05	1.45	SMAD family member 9
	OPTN*	2.1E−04	1.24	Optineurin
	*WNT4*	4.4E−04	1.78	Wnt family member 4
	*NOTCH1*	4.8E−04	1.08	Notch 1
	*F3*	7.1E−04	1.53	Coagulation factor III, tissue factor
G10: regulation of axon guidance				
	SEMA3F*	3.6E−12	2.15	Semaphorin 3F
	*ENC1*	7.2E−07	1.76	Ectodermal-neural cortex 1
	RHOJ*	2.8E−06	2.50	Ras homolog family member J
	CDH1*	2.8E−05	2.57	Cadherin 1
	*LRP1*	5.8E−05	1.30	LDL receptor–related protein 1
	*LAMA2*	8.0E−05	1.62	Laminin subunit alpha 2
	PTPRM*	1.0E−04	1.50	Protein tyrosine phosphatase, receptor type M
	*DDR2*	1.2E−04	1.79	Discoidin domain receptor tyrosine kinase 2
	*LDLRAD4*	1.6E−04	1.90	Low-density lipoprotein receptor class A domain containing 4
	ISL1*	1.6E−04	2.08	ISL LIM homeobox 1
	*PDZD2*	2.3E−04	1.90	PDZ domain containing 2
	*PLPP3*	2.8E−04	1.14	Phospholipid phosphatase 3
	*ADRA2A*	3.4E−04	2.10	Adrenoceptor alpha 2A
	*LAMA4*	3.4E−04	1.74	Laminin subunit alpha 4
	*PLEKHG5*	4.0E−04	1.33	Pleckstrin homology and RhoGEF domain containing G5
	*CXCL16*	4.5E−04	2.48	C-X-C motif chemokine ligand 16
	*ERBB4*	6.0E−04	1.28	erb-b2 receptor tyrosine kinase 4
	SEMA5B*	6.9E−04	1.29	Semaphorin 5B
G8: axon extension involved in axon guidance				
	SEMA3F*	3.6E−12	2.15	Semaphorin 3F
	ISL1*	1.6E−04	2.08	ISL LIM homeobox 1
	SEMA5B*	6.9E−04	1.29	Semaphorin 5B
G7: sensory organ development				
	*RPE65*	1.8E−14	3.61	RPE65, retinoid isomerohydrolase
	*SLITRK6*	4.2E−08	2.58	SLIT and NTRK like family member 6
	RHOJ*	2.8E−06	2.50	Ras homolog family member J
	CDH1*	2.8E−05	2.57	Cadherin 1
	*ADGRV1*	6.6E−05	1.63	Adhesion G protein-coupled receptor V1
	PTPRM*	1.0E−04	1.50	Protein tyrosine phosphatase, receptor type M
	OPTN*	2.1E−04	1.24	Optineurin
	*OLFM3*	2.9E−04	1.70	Olfactomedin 3
	*JAG2*	9.6E−04	1.57	Jagged 2
G6: detection of external stimulus				
	*NTSR1*	8.2E−07	2.18	Neurotensin receptor 1
	TTN*	2.6E−05	2.30	Titin
	*PRDM12*	3.7E−05	2.16	PR/SET domain 12
G5: regulation of lipid localization				
	PPARA*	5.4E−04	1.26	Peroxisome proliferator activated receptor alpha
	*ABCG1*	6.0E−05	1.90	ATP binding cassette subfamily G member 1
G4: cell-cell adhesion via plasma-membrane adhesion molecules				
	*SLITRK2*	7.6E−07	2.70	SLIT and NTRK like family member 2
	*AMIGO2*	8.2E−07	2.08	Adhesion molecule with Ig like domain 2
	*PCDHA12*	6.0E−04	1.73	Protocadherin alpha 12
	*PCDHGA11*	9.7E−04	1.50	Protocadherin gamma subfamily A, 11
G2: mesenchyme development				
	*RANBP3L*	4.3E−05	3.61	RAN binding protein 3 like
G1: muscle hypertrophy				
	*MYH7*	5.3E−06	2.02	Myosin heavy chain 7
	TTN*	2.6E−05	2.30	Titin
	PPARA*	5.4E−04	1.26	Peroxisome proliferator activated receptor alpha

List of most significant (padj< 1 × 10^−3^) upregulated univocal genes classified according to the respective GO terms groups. *p* values (padj), expression change levels (LFC, logarithmic fold change) and annotations are provided. Asterisks (*) indicate top DEGs associated to multiple (up to 2) groups. Some GO groups in Fig. 4 are not in the tables because not containing top DEGs

**Table 2:** RSTS top univocal DRGs sorted into GO terms clusters

	Gene	padj	LFC	Annotation
G7: RNA processing				
	LYAR*	8.1E−08	− 1.64	Ly1 antibody reactive
	*HNRNPA1*	5.6E−06	− 1.24	Heterogeneous nuclear ribonucleoprotein A1
	TTK*	7.3E−06	− 1.59	TTK protein kinase
	HPRT1*	2.6E−05	− 1.36	Hypoxanthine phosphoribosyltransferase 1
	FBL*	2.9E−05	− 1.30	Fibrillarin
	*MAGOHB*	1.0E−04	− 1.35	Mago homolog B, exon junction complex core component
	SNRPD1*	1.6E−04	− 1.20	Small nuclear ribonucleoprotein D1 polypeptide
	*TNFRSF1B*	2.3E−04	− 3.06	TNF receptor superfamily member 1B
	RPS6*	2.4E−04	− 1.15	Ribosomal protein S6
	SNRPF*	2.5E−04	− 1.16	Small nuclear ribonucleoprotein polypeptide F
	NOP58*	4.4E−04	− 1.09	NOP58 ribonucleoprotein
	RUVBL1*	5.1E−04	− 1.16	RuvB like AAA ATPase 1
	LY6E*	5.6E−04	− 1.31	Lymphocyte antigen 6 complex, locus E
	SNRPD2*	7.0E−04	− 1.13	Small nuclear ribonucleoprotein D2 polypeptide
	*METTL1*	7.3E−04	− 1.19	Methyltransferase like 1
	SNRPG*	8.0E−04	− 1.20	Small nuclear ribonucleoprotein polypeptide G
	RPS3*	8.7E−04	− 1.02	Ribosomal protein S3
G6: DNA metabolic process				
	*DBF4B*	1.1E−06	− 1.66	DBF4 zinc finger B
	*TIPIN*	4.5E−05	− 1.42	TIMELESS interacting protein
	POLD2*	3.3E−04	− 1.01	DNA polymerase delta 2, accessory subunit
	DAXX*	5.4E−04	− 1.11	Death domain–associated protein
	*CDK1*	5.5E−04	− 1.30	Cyclin-dependent kinase 1
	*ABRAXAS1*	6.5E−04	− 1.12	BRCA1 A complex subunit
	NME1*	6.8E−04	− 1.15	NME/NM23 nucleoside diphosphate kinase 1
	*FANCB*	7.1E−04	− 1.83	Fanconi anemia complementation group B
	RPS3*	8.7E−04	− 1.02	Ribosomal protein S3
	PRIM1*	9.3E−04	− 1.13	DNA primase subunit 1
G5: purine ribonucleoside monophosphate biosynthetic process				
	HPRT1*	2.6E−05	− 1.36	Hypoxanthine phosphoribosyltransferase 1
	*PRPS2*	4.3E−05	− 1.29	Phosphoribosyl pyrophosphate synthetase 2
	*NPPB*	2.3E−04	− 2.19	Natriuretic peptide B
	*PAICS*	3.9E−04	− 1.03	Phosphoribosylaminoimidazole carboxylase
	*PPCDC*	9.6E−04	− 1.24	Phosphopantothenoylcysteine decarboxylase
G4: ribonucleoprotein complex biogenesis				
	LYAR*	8.1E−08	− 1.64	Ly1 antibody reactive (LYAR)
	TTK*	7.3E−06	− 1.59	TTK protein kinase (TTK)
	FBL*	2.9E−05	− 1.30	Fibrillarin (FBL)
	SNRPD1*	1.6E−04	− 1.20	Small nuclear ribonucleoprotein D1 polypeptide (SNRPD1)
	RPS6*	2.4E−04	− 1.15	Ribosomal protein S6 (RPS6)
	SNRPF*	2.5E−04	− 1.16	Small nuclear ribonucleoprotein polypeptide F (SNRPF)
	NOP58*	4.4E−04	− 1.09	NOP58 ribonucleoprotein (NOP58)
	RUVBL1*	5.1E−04	− 1.16	RuvB like AAA ATPase 1
	LY6E*	5.6E−04	− 1.31	Lymphocyte antigen 6 complex, locus E
	SNRPD2*	7.0E−04	− 1.13	Small nuclear ribonucleoprotein D2 polypeptide (SNRPD2)
	SNRPG*	8.0E−04	− 1.20	Small nuclear ribonucleoprotein polypeptide G (SNRPG)
G2: telomere maintenance				
	POLD2*	3.3E−04	− 1.01	DNA polymerase delta 2, accessory subunit
	NME1*	6.8E−04	− 1.15	NME/NM23 nucleoside diphosphate kinase 1
	PRIM1*	9.3E−04	− 1.13	DNA primase subunit 1
G1: cellular response to antibiotic				
	DAXX*	5.4E−04	− 1.11	Death domain–associated protein

List of most significant (padj< 1 × 10^−3^) downregulated univocal genes classified according to the respective GO terms groups. *p* values (padj), expression change levels (LFC, logarithmic fold change), and annotations are provided. Asterisks (*) indicate top DEGs associated to multiple (up to 2) groups. Some GO groups in Fig. 4 are not in the tables because not containing top DEGs

Out of RSTS univocal URGs, we underline the most significant top genes, which control phenotypes well recognizable in our in vitro model and hence candidates to account for the morpho-functional alterations detected in neurons from the same patients [24]. This applies to the semaphorin genes *SEMA3F* and *SEMA5B*, encoding members of a family of signaling proteins first described as axon guidance cues and then implicated in multiple aspects of nervous system development [41], which are sorted under the groups “regulation of axon guidance” (G10) and “axon extension involved in axon guidance” (G8). The most gene-enriched group G10, also includes *ENC1* (ectodermal-neural cortex 1) encoding an actin-binding protein favoring cell matrix adhesion, *RHOJ* (Ras Homolog family member 1) encoding a small GTP-binding protein of the Rho family regulating cytoskeleton/cell polarity and *CDH1* for E-cadherin, a calcium-ion-dependent protein, principal component of polarity, and intercellular adhesion. Other G10 URGs involved in adhesive function are *PTPRM* (for a tyrosine phosphatase which dephosphorylates components of cadherin-catenin complexes) and *LAMA2*/*LAMA4* (alpha 2/alpha 4 subunits of laminin, an extracellular matrix protein with a key role in neural development. The G7 “Sensory organ development” group shows as most significant the *RPE65* gene for the retinoid isomerohydrolase protein, converting all trans retinyl esters to 11 cis retinol in the retinal pigment epithelium, followed by *SLITRK6*, encoding a member of SLIT-like and TRK-like (SLITRK) family proteins, involved in extracellular axon guidance, neurite extension, and cell motility [42], plus the abovementioned cell polarity genes *CDH1* and *RHOJ*. The G4 group “cell-cell adhesion via plasma-membrane adhesion molecules” includes the top gene *SLITRK2* (of the same family of *SLITRK6)*, *AMIGO2* for a transmembrane protein involved in cell adhesion, and the protocadherin genes *PCDHA11*and *PCDHA12* (Table 1). Other members of the PCDH family, *PCDHB8* and *PCDH11Y*, are in the same group (Additional File 10). As shown in Table 1, several URGs are sorted to groups related to processes apparently less applicable to our in vitro model, though they need to be inspected as “diseased” cells are present in vivo in the context of the whole organism. Examples are the G11 “urogenital system development” enriched *NFIA* and *SALL1* transcription factors genes and *SMAD9*, *WNT4*, and *NOTCH1*genes that act in fundamental intercellular signaling pathways. In particular*,* WNT4, functioning as non-canonical WNT, has been suggested to promote early neural differentiation and to play a role in differentiation of certain types of neurons through the expression of the *ASCL1* early neuronal gene [43], which is also upregulated in RSTS neurons (Additional File 10).

Processes enriched from RSTS DRGs concern RNA/DNA metabolism and are mainly sorted into three GO terms groups (Table 2 and Additional File 11). The related G7 (RNA processing) and G4 (ribonucleoprotein complex biogenesis) groups share the top genes *RUVBL1* (RuvB like AAA ATPase 1) and *LY6E* (lymphocyte antigen 6 complex, locus E). The G7 top gene *LYAR* encoding a cell growth regulating nucleolar protein is followed by genes for other structural cell proteins, such as *HNRNPA1* for heterogeneous nuclear ribonucleoprotein A1, *MAGOHB* for a component of the exon-exon splicing complex, *FBL* for fibrillarin, *RPS3* and *RPS6* for the respective ribosomal proteins, *NOP58* for the NOP58 ribonucleoprotein, and the *SRNP* family members for the small nuclear ribonucleoproteins D1, D2, F, and G.

The G6 group “DNA metabolic process” is enriched in genes for basic cell processes, such as the DNA replication genes, *DBF4B* for the regulatory subunit of the S-phase kinase, *TIPIN* encoding a protein associated with the components of the MCM7 replicative helicase, *POLD2* for the regulatory subunit of polymerase delta 2, and *PRIM1* for the subunit of the DNA polymerase/primase complex.

### Differential Expression of Markers of the Neuronal Differentiation Between RSTS and Controls

**Fig. 5:**
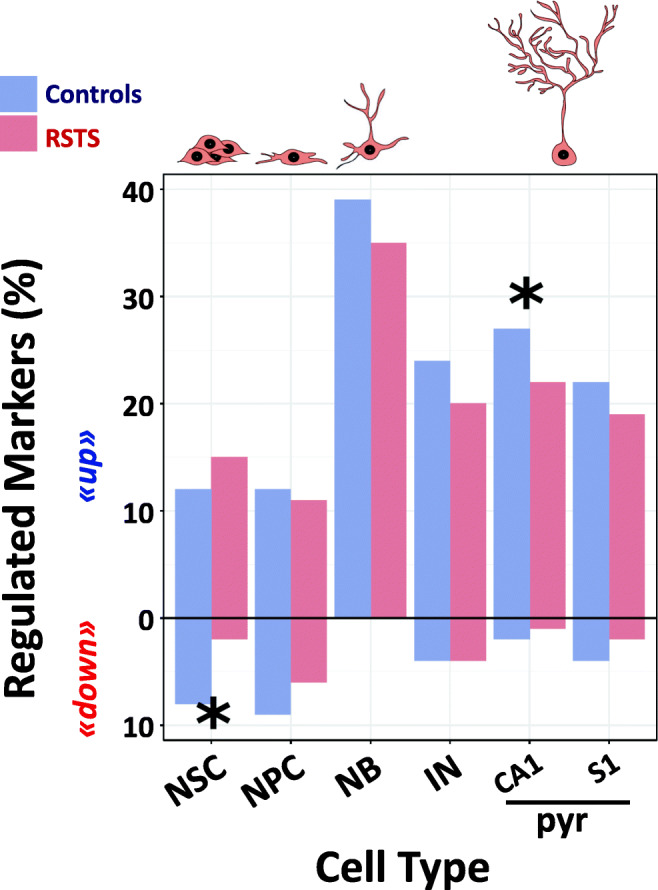
Differential expression of RSTS and controls neurodevelopmental specific markers. Percentage of developmental gene markers found regulated in healthy controls and RSTS patients during transition from iNeurons to NPCs. The gene markers are indicated for the different cell types (NSC: neural stem cells, NPC: neural progenitor cells, NB: neuroblasts, IN: immature neurons, pyr: pyramidal neurons in hippocampal (CA1) and cortical (S1) regions). Asterisks point to significant changes in a one-way *Z* test (NSC downregulated markers *p* = 4.0E−9, CA1pyr upregulated markers *p* = 0.02)

Next, we focused on genes that were identified in single cell RNA-seq (scRNA-seq) screens [44, 45] as markers for five different developmental stages during neurogenesis in rodents: neural stem cells (NSC), neural progenitor cells (NPC), neuroblasts (NB), immature neurons (IN), and mature pyramidal hippocampus (CA1) together with somatosensory cortex (S1) neurons. We found that the total number of differentiation markers differentially expressed in the comparison between iNeurons and NPCs in controls and RSTS is higher in controls (326) than RSTS (268) cells. Moreover, the cells from controls exhibit more downregulation of NSC markers and more upregulation of mature pyramidal neuron markers (Fig. 5). The top gene downregulated in control (Log2FC − 2.7, padj = 7.8 × 10–7) but not in RSTS iNeurons is *ID3*, the hortolog of mouse *id3*, which encodes an inhibitor of proneural transcription factors whose upregulation prevents the terminal differentiation of hippocampal neurons [46]. On the other hand, genes such as the mouse hortologs *CPNE6* (Log2FC 4.7, padj = 5.1 × 10–7; encoding neuronal Copine 6, a calcium, and phospholipid binding protein involved in synaptic maturation and plasticity) [47] and *KCNA4* (Kv1.4) (Log2FC 3.7, padj = 1.6 × 10–5; encoding a voltage-gated potassium channel associated with neuronal maturation both in vivo and in culture) [48] are strongly upregulated in control iNeurons (when compared to NPCs), but not so in RSTS iNeurons.

These results further support the notion that the differentiation process is either impaired or delayed in RSTS cells. In either case, the process of acquisition of neuronal identity is compromised in RSTS.

## Discussion

The in vitro neuronal iPSC model generated for Rubinstein-Taybi syndrome [22–24] provided us the platform to search for dysregulated gene pathways which might lead to the morphological and electrophysiological alterations of RSTS neurons appointed as biomarkers of the neurocognitive signs of the patients. This aim has translational relevance because, at difference of *CREBBP/EP300* causative gene mutations, the resulting epigenetic modifications are reversible and have been demonstrated to impact both brain development and adult brain function raising the opportunity of postnatal treatment with known and novel compounds to ameliorate the cognitive impairment of the patients [7, 49].

Aiming at gathering a better understanding of the molecular mechanisms underlying intellectual disability in RSTS, we performed whole transcriptional analyses on neural progenitors and neurons of iPSC reprogrammed from peripheral blood of five RSTS patients.

**Fig. 6:**
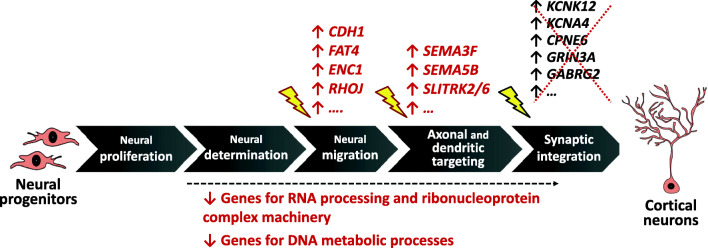
Summary of main transcriptional signatures in RSTS iNeurons. Differentiation from neural progenitors to cortical neurons is depicted by stages, named according to [51]. RSTS univocal upregulated genes (red arrows upon the schematic diagram) include polarity/adhesion and axon guidance genes aberrantly switched on, while RSTS univocal downregulated genes (red arrows below the scheme) are mainly RNA and DNA metabolism genes. Besides these up and down “active modulation” signatures, the RSTS transcriptome is characterized by “passive modulation,” i.e., the lack of genes (crossed out and indicated by blue arrows upon the scheme) functional to synaptic integration, active in controls. The yellow arrows point to the plausible neural differentiation stage at which the indicated genes should act. No relationship with specific neural differentiation stage(s) can be hypothesized for the downregulated RNA and DNA genes

The analyses captured significant DEGs between the two time points: NPCs and post-mitotic neurons, but not between patients and controls at both stages. This result may be due to the variability in the genetic background and in the cognitive deficit of the modeled RSTS patients as well as to the unavoidable differences in culture conditions, neuronal differentiation efficiency of iPSCs [50], and functional maturation of iNeurons from patients and controls, despite application of the same protocol [29]. Relevant to the difficulties in retrieving significant DEGs in our RSTS vs control neuronal samples are also the findings that the elimination of both *Crebbp* and *Ep300* genes in mouse embryonic fibroblasts led to reduction of a multitude of acetylation sites on histone and transcription factors, but the resulting transcriptional changes were modest, due to the rapid dynamics of CBP/p300 acetylome [19]. In keeping with these data and contrary to the broad scope of downstream targets resulting from CBP/p300 acetylation of enhancer-associated transcriptional regulators, the transcription of only a subset of genes was affected in our haploinsufficient/defective *CREBBP/EP300* neuronal model. This subset is represented by the control- and RSTS-specific DEGs observed across the time course of neuronal differentiation, which made informative the RSTS/control paired “over time “transcriptome analysis. First, we noticed that modulation of gene expression is weakened in RSTS as the overall DEGs number is lower respect to control neurons (Fig. 2a). The comparative analysis of controls and RSTS revealed a bulk of shared biological processes (GO terms) (Fig. 2b) and DEGs (Fig. 3a), a finding accounting for the successful differentiation of patients neurons evaluated by qualitative expression of stage-specific immunohistochemical markers in all respective neuronal cultures [24] (Additional File 1). Focus on univocal DEGs lists showed in RSTS improperly upregulated genes of cell polarity and adhesion acting in neuronal migration such as *CDH1*, *FAT4*, and *ENC1* and genes for axonal and dendritic targeting such as *SEMA3F*, *SEMA5B*, and *SLITRK2/6* (Fig. 6, upturned red arrows on the top of the schematic). The picture emerging by over-representation from DRGs is even more striking as peculiar to RSTS is the sharp collapse of the RNA processing/ribonucleoprotein complex machinery and DNA metabolic processes attested by > 35 DRGs for these functions (Fig. 6, downturned red arrows below the schematic).

Besides this “active” modulation, “passive” modulation (de-regulation of genes regulated in controls) appears a distinctive feature of the RSTS transcriptome. To exemplify this concept, some genes acting in the late stage of synaptic integration, sorted as URGs in controls, likewise ion channels, neurotransmitters, and neurotransmitter receptor genes, were not active in RSTS (Fig. 6, crossed out genes with upturned blue arrows on the top of the schematic).

Neural differentiation markers represent an interesting subset of DEGs as they inform our understanding of the affected neurodevelopmental stage and cell type. By using the list of markers set up by scRNA-seq during the developmental stages of rodents neurogenesis [44, 45], we recorded in controls significant more downregulation and upregulation than in RSTS at the transition from stem cells to neural progenitors and in the terminal differentiation to pyramidal neurons, respectively (Fig. 5). Passive gene modulation likely contributes to these differences, as also inferred by the lower number of total marker DEGs in RSTS than in controls. It would be suitable to our human neuronal system to go beyond mouse differentiation markers using the information provided by a single cell RNASeq study on human iPSC-derived organoids modeling human brain development [52] to seek the overlap between the cell stage and cell-type-specific markers and the differentially expressed genes in the progression from progenitors to iNeurons in RSTS and control cells. However, studies with human fetal tissue or cells cannot separate the different stages with the same precision than the mouse studies and actually, some of the DEGs clusters in the mentioned iPSC-derived human organoid study [52] could not be recognized in their cell type origin, advising to wait for more complete information to pursue this analysis on our RSTS neuronal model.

In order to link the significant DEGs emerging from our study to the morphological alterations and the hypoexcitability of RSTS iNeurons RSTS, we list the best candidates according to the putative stage of development at which they should act (Table 1 and Fig. 6). Incorrect upregulation of genes involved in axonal guidance through neuronal process formation (*ENC1*) assemblage of cytoskeletal components for establishment of motility and cell polarity (*RHOJ*), and cell adhesion and synaptic plasticity (*CDH1*) [53] undoubtedly affects neuronal morphology. The same applies to the top URGs *SEMA3F* and *SEMA5B* genes that regulate dendritic morphology and excitatory and inhibitory synaptogenesis [41]. To note that, the genes for semaphorin receptors, neuropilin 1 (*NRP1*), and plexin A3 (*PLXNA3*) that mediates cell adhesion via a homophilic binding in the presence of calcium ions were also upregulated in RSTS neurons (Additional File 10) highlighting the activation of the semaphorin-plexin axis which usually orchestrates central nervous system connectivity through the differential control of spine morphogenesis and synapse formation [54]. Incorrect activation of this axis may account for reduced branch length and increased branch number shown by 42 days iNeurons of the same patients [24]. Furthermore, aberrant upregulation of *SEMA3F* along with the lack of some genes essential for synaptic signaling and neurotransmission, such as ion voltage-gated activity, neurotransmitter, and neurotransmitter receptors (passive gene modulation), may concur to the hypoexcitability recorded in the 70 days iNeurons [24]. Other RSTS URGs with a role in the organization of neuronal synapses are the postsynaptic *SLITRKs* (which selectively bind specific members of the presynaptic type IIa receptor protein tyrosine phosphatase family) [55]. In RSTS iNeurons, the upregulated *SLITRK2* and *SLITRK6* and the improper *PTPRM* and *PTPRR* receptors (Additional File 10) may act in concert mimicking synaptogenic activity.

Impairment in synaptic structure and integrated function has been pointed out as common pathophysiology across NDDs [56]. Synaptic dysfunction and decreased excitability of RSTS iNeurons may contribute to cognitive impairment of the patients [24] as shown for iNeurons of patients with Rett syndrome [29, 57] and idiopathic autism [26]. Genes which regulate axon growth and pathfinding as well as terminal branching of axons such as *SEMA3C* and *SLITRK2* and *SLITRK4* were found downregulated in iNeurons from patients with Fragile X syndrome, an ID disorder associated with epigenetic dysregulation [30]. Downregulation of *SLIT1* gene (alias *MEGF4*) together with other key players of axonal guidance was observed in the first generated *FMR1* iPSC-derived neurons [31], while *MEGF8*, a member of the same family, is among the URGs in our RSTS neurons. Thus, dysregulation of axon guidance/extension is shared by RSTS and FRAXA syndromes, with a different transcriptional profile of the same key genes.

The downregulation of cell maintenance processes, a major culprit of the defective RSTS transcriptome, has been also recognized as underlying mechanism of autism spectrum disorders (ASD) [26], a molecular finding consistent with the frequent occurrence of behavioral alterations in RSTS patients, displayed by three of the five donors of the iNeurons herein analyzed. We found that CBP/p300 regulated genes were overlapped with the targets of *CDH8*-caused autism, including cell cycle and cytoskeleton and cell adhesion genes and ribonucleprotein complex genes [27]. Axonal guidance and extracellular matrix components genes were either upregulated or downregulated in *CHD8+/−* neurons [58]. An exemplary case is the *FAT3* gene, encoding an atypical cadherin associated with large brain volume or head size downregulated in *CDH8+/−* neurons [27], while *FAT4*, a gene also encoding an atypical cadherin, is upregulated in RSTS neurons and associated with an opposite clinical phenotype, i.e., microcephaly, a universal feature of RSTS patients. Another candidate gene for autism, *ISL1* (ISL LIM homeobox 1), significantly upregulated in our RSTS neurons (Table 1), was found downregulated in individuals with duplications of chromosome 15q11-q13.1, which account for 1 to 3% of all autism cases [32]. Again, there are convergent genes dysregulated in NDDs, though with a different profile.

In conclusion, our RNA-Seq study unveiled in RSTS cells hallmark features of dysregulation in the course of differentiation from neural progenitors to post-mitotic neurons (Fig. 6). RSTS transcriptome is quantitatively and qualitatively defective due to aberrant upregulation of genes involved in neural migration and axonal and dendritic targeting and downregulation of RNA and DNA metabolic genes (“active” gene modulation). De-regulation of some genes involved in synaptic integration, also suggested by reduction of excitatory neurons markers as compared to controls (“passive” gene modulation), is a signature of the RSTS transcriptome landscape (Fig. 6). The altered dendritic morphology and the electrophysiological defects revealed in RSTS iNeurons [24] provide functional support to the RSTS transcriptome data. These neuronal biomarkers predict to lead to defective neuronal performance during development and adult life and may thus contribute to elucidate the cognitive deficit and behavioral disorder of RSTS patients.

Last, downregulation in RSTS iNeurons of a huge number of genes for ribosome biogenesis, RNA modifications, and DNA metabolism suggests that RSTS is a global transcription disorder, as proposed for Cornelia de Lange, another syndrome caused by chromatin dysregulation [59]. The contribution of massive downregulation of genes for RNA and DNA maintenance processes to cognitive impairment deserves to be elucidated, as interestingly this transcriptomic signature is shared by other intellectual disability syndromes and idiopathic autism [26, 59].

## Methods

### Study Design and Subjects

This study was approved by the Institutional Ethical Committee of IRCCS Istituto Auxologico Italiano, Milan 2015 Dec 15. Written informed consent was obtained from all patients’ parents and control subjects. Expression analysis was conducted on neural progenitors (35 days of differentiation) and mature neurons (70 days of differentiation) obtained from iPSC lines generated from 5 RSTS patients (4 *CREBBP*- (P34, P46, P149, and P158) and 1 *EP300*-mutated (P207)). Three of the above RSTS iPSC lines were registered on the dedicated Human Pluripotent Stem Cell Registry (https://hpscreg.eu/): P34 (IAIi002-A), P46 (IAIi003-A), and P149 (IAIi004-A). The reprogramming and neural differentiation workflow are described in detail in [24]. In brief, iPSCs were generated from peripheral blood mononuclear cells applying after 9 days of enrichment of the cells to be reprogrammed (erythroblasts) the integration-free Sendai virus kit and then plating cells onto mouse embryonic fibroblasts (MEFs) in human embryonic stem cell medium [60]. Emerging colonies were picked up since day 20, cut and transferred to new feeder layer coated wells, and characterized by karyotyping, array CGH, and original mutation. iPSC clones bypassing the genome stability check were then differentiated into cortical neurons in Neurobasal medium supplemented with noggin [32]. Once neural rosettes appeared, they were passaged on polyornithine-laminin coated dishes and maintained for further 2 weeks. At day 28, the Neurobasal medium was changed to neural differentiation medium. After 1 week, neural progenitors (day 35) were plated at low density for terminal differentiation. NPCs and iNeurons were also obtained through the same workflow from 4 healthy individuals used as control cohort.

### RNA Extraction

Total RNA was extracted with Quick-RNA MiniPrep Kit (Zymo Research) including a DNase digestion step to remove any residual genomic DNA contamination. RNA quality was assessed through the RNA 6000 Nano Kit using an Agilent Bioanalyzer (Agilent). RNA integrity number (RIN) was determined for every sample and only samples with RIN > 7.5 were selected for the RNASeq analysis. RNA concentration was estimated using a Nanoquant Infinite M200 instrument (Tecan).

### RNA-Seq Library Preparation and Sequencing

Sequencing libraries were prepared in two batches using TruSeq Stranded mRNA Library Prep kit (Illumina). One hundred nanograms of total RNA was used as input. Polyadenylated transcripts were purified using poly-T oligo-attached magnetic beads. RNA samples were fragmented at 94 °C for 8 min and retro-transcribed to cDNA using random hexamer primers. All cDNAs were indexed and amplified with 15 PCR cycles. Final libraries were validated with the Agilent DNA 1000 kit and sequenced on a NextSeq500 platform (Illumina), producing 75 × 2 bp paired-end reads.

### Sequencing Data Analysis

Quality control of raw data was performed by using the FastQC tool (v.0.11.5) (https://www.bioinformatics.babraham.ac.uk/projects/fastqc/). Alignment of high-quality paired-end reads to the reference genome (GRCh38) was conducted using STAR (spliced transcripts alignment to a reference) (version 2.5.2b) [61] enabling “outFilterMultimapNmax 1” parameter in order to return and consider alignments that map to one locus only: reads that map to 2 or more loci were considered unmapped. A further quality control step checked several metrics such as coverage distribution across gene length and percentage of reads mapping to exons. Uniquely mapping reads were allocated to genes with featureCounts (version 1.5.1) [62]. Parameters were setup in order to take into account only fragments with both ends mapped and reads overlapping the exons with at least 10 nucleotides (--minOverlap 10). Gencode genes primary assembly (release v.29) was used as annotation source for genomic feature boundaries. We obtained a matrix of counts for each sample (columns) for each gene (rows) (58,721 genes according to gencode v.29).

### Exploratory Data Analysis

Principal component analysis (PCA) was performed using DESeq2 plotPCA function on regularized log transformed counts matrices created by DESeq2 rlog function [63].

### Statistical Analyses

Statistical analyses were performed using R environment. Differential gene expression (DGE) analysis was performed using functions implemented in DESeq2 package. DGE analysis was firstly conducted between patients and controls at either the two time points (35 and 70 days of differentiation). A second round of DGE analysis was carried out by comparing expression profiles of mature neurons to NPCs of patients and controls, separately, using paired analysis (using patients as covariate). Genes with a padj< 0.01 (Benjamini–Hochberg (False Discovery Rate)) were considered differentially expressed (DEGs). Identification of DEGs-enriched biological processes was carried out by using the Cytoscape (v. 3.7.1) [64] and ClueGO plugin (v. 2.5.4 (GO-BP-EBI-UniProt-GOA-27.02.2019) [65]. Over-representation analysis (ORA) was based on a right-sided hypergeometric test using multiple testing correction Bonferroni step down method: terms were grouped using kappa statistics (*K* = 0.4). Two *p* values thresholds for significant clusters were set: (i) padj< 0.01 (from all DEGs of controls and RSTS) and (ii) padj< 0.05 (from univocal DEGs lists). Differential expression of RSTS and controls in neurodevelopmental specific markers was evaluated by using one-way *Z* test. Significance was set to 0.05.

### Data Visualization

PCA charts and volcano plots were produced by “ggplot2” and “graphics” packages in R, respectively. Venn diagrams were obtained by using a web tool (http://bioinformatics.psb.ugent.be/webtools/Venn/). Clustering of biological processes was visualized using a tool such as ReviGO (http://revigo.irb.hr/) [40] setting “Allowed similarity” to Medium (0.7) and using UniProt-to-GO mapping file “goa_uniprot_gcrp.gaf.gz” dated 15 March 2017.

## Electronic Supplementary Material

ESM 1 **Additional File 1 (Additional_File_1.pdf)**. **Immunofluorescence (IF) characterization of iPSC-derived neural rosettes, early and mature neurons from Control (C1), and RSTS patients 46, 34 and 149.** The IF positive staining of the neuroectodermal stem cell markers NESTIN and PAX6, the pan-neuronal cell markers MAP2 (microtubule-associated protein 2) and TUJ1 (beta-III tubulin) and the cortical markers CUX-1 (cut-like homeodomain transcription factor) and TUJ1 can be seen at rosette and early and mature neuron stages on coverslips from the indicated samples. Nuclei are counterstained with DAPI. Differences between C1 and patients and between individual patients are evident at the early neurons stage when the low cell density allows to monitor neuronal morphology. Obj.: Rosettes = 20x; Early (42 days) neurons =40x; Mature neurons = 20x. (PDF 661 kb)

ESM 2 **Additional File 2 (Additional_File_2.pdf)**. **Visualization of RSTS patients mutations.** IGV (Integrative Genome Viewer) snapshots of reads alignments showing patients specific mutations in *CREBBP* (**top panel – patients P34, P46, P149 and P158**) and *EP300* (**bottom panel – patient P207**). Data refer to human assembly GRCh38/hg38. (PDF 285 kb)

ESM 3 **Additional File 3 (Additional_File_3.pdf)**. **Heatmaps of controls and RSTS DEGs.** Heatmaps of the expression profile of DEGs at the two time points (iNeurons vs neural progenitors) in **(a)** controls and **(b)** RSTS patients, by using 3399 and 2712 DEGs, respectively. Each column represents a sample and each row represents a differentially expressed gene. Gene expression levels were normalized to z-score. Differences in expression are displayed through a color graduation: brown tones represent up-regulation while light blue tones represent down-regulation. Figures were obtained in R environment by using “heatmap.2” function of “gplots” package. (PDF 366 kb)

ESM 4 **Additional File 4 (Additional_File_4.pdf)**. **Gene Ontology (GO) enrichment of URGs of controls and RSTS groups.** List of significant (padj<0.01) biological processes enriched in controls (*n* = 279) and RSTS (*n* = 257) URGs. Group name, GO terms identification codes and relative names are reported in the first three columns, respectively. Additional columns show supplementary information related to GO terms enrichment: in particular, last column refers to the shared/univocal status of the go term between RSTS and controls. (PDF 499 kb)

ESM 5 **Additional File 5 (Additional_File_5.pdf)**. **Gene Ontology (GO) enrichment of DRGs of controls and RSTS groups.** List of significant (padj<0.01) biological processes enriched in controls (*n* = 135) and RSTS (*n* = 155) DRGs. Group name, GO terms identification codes and relative names are reported in the first three columns, respectively. Additional columns show supplementary information related to GO terms enrichment: in particular, last column refers to the shared/univocal status of the GO term between RSTS and controls. (PDF 347 kb)

ESM 6 **Additional File 6 (Additional_File_6.pdf)**. **REVIGO treemaps of 224 up- biological processes shared by RSTS and controls.** REVIGO treemaps summarizing Gene Ontology (GO) **(a)** biological processes and **(b)** molecular functions URGs-enriched categories (224) shared by RSTS and controls (**see** Fig. 2b**- left side**). For each panel, not all URGs-enriched terms are reported due to space constraints. (PDF 1868 kb)

ESM 7 **Additional File 7 (Additional_File_7.pdf)**. **REVIGO treemap of 102 down- biological processes shared by RSTS and controls.** REVIGO treemap summarizing Gene Ontology (GO) biological processes DRGs-enriched categories (102) shared by RSTS and controls (**see** Fig. 2b**- right side**). For each panel, not all DRGs-enriched terms are reported due to space constraints. (PDF 843 kb)

ESM 8 **Additional File 8 (Additional_File_8.pdf)**. **Gene Ontology (GO) enrichment of univocal URGs of control and RSTS groups.** List of significant (padj<0.05) biological processes enriched in controls (*n* = 74) and RSTS (*n* = 43) URGs. Groups, GO terms identification codes and names are reported in the first three columns, respectively. Additional columns show information related to GO terms clustering/enrichment. (PDF 239 kb)

ESM 9 **Additional File 9 (Additional_File_9.pdf)**. **Gene Ontology (GO) enrichment of univocal DRGs of controls and RSTS groups.** List of significant (padj<0.05) biological processes enriched in controls (*n* = 80) and RSTS (*n* = 44) DRGs. GO terms identification codes and names are reported in the first three columns, respectively. Additional columns show information related to GO terms clustering/enrichment. (PDF 243 kb)

ESM 10 **Additional File 10 (Additional_File10.pdf)**. **Cluster analysis of univocal-URGs enriched GO terms (Controls and RSTS)**. Cluster lists from GO terms enriched in univocal controls and RSTS URGs. Relative group pvalues (corrected with Bonferroni step down), percentage of GO terms and gene names associated to clusters are provided in additional columns. (PDF 189 kb)

ESM 11 **Additional File 11 (Additional_File_11.pdf)**. **Cluster analysis of univocal-DRGs enriched GO terms (Controls and RSTS)**. Cluster lists from GO terms enriched in univocal controls and RSTS DRGs. Relative group pvalues (corrected with Bonferroni step down), percentage of GO terms and gene names associated to clusters are provided in additional columns. (PDF 197 kb)

## Data Availability

All data are deposited to GEO (https://www.ncbi.nlm.nih.gov/geo/) with accession number GSE135287. The complete lists of controls and RSTS DEGs are available upon request.
