# Supplementary material for: Transcriptome Analysis of iPSC-Derived Neurons from Rubinstein-Taybi Patients Reveals Deficits in Neuronal Differentiation
Source: Mol Neurobiol. 2020 Jun 20;57(9):3685–701. doi: 10.1007/s12035-020-01983-6 (PMC7399686; doi:10.1007/s12035-020-01983-6)

Additional file 1

Immunofluorescence (IF) characterization of iPSC-derived neural rosettes, early and mature neurons from Control and RSTS patients 46, 34 and 149.

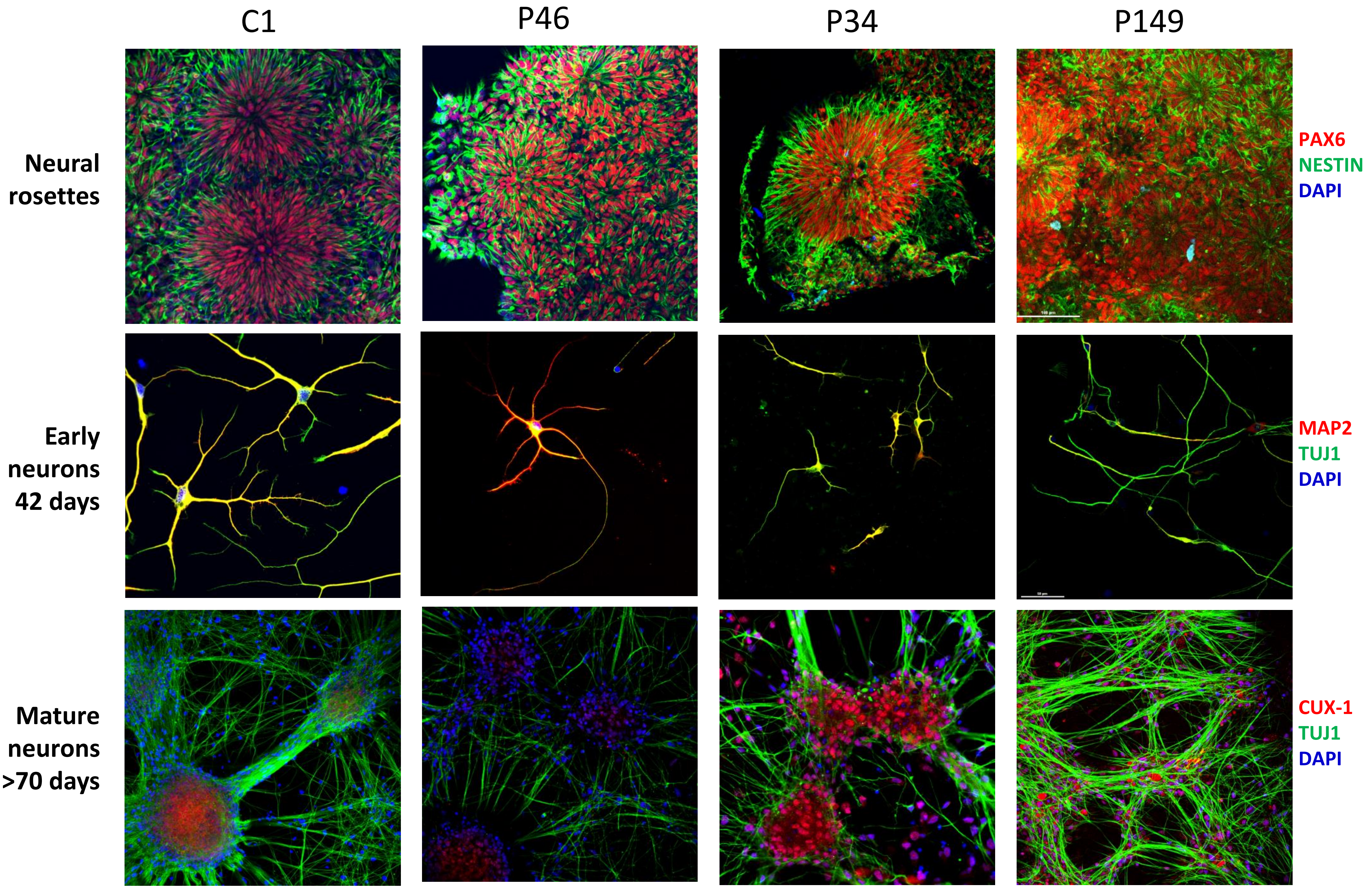

Supplement: Supplementary file 1 — Additional File 1 (Additional_File_1.pdf). Immunofluorescence (IF) characterization of iPSC-derived neural rosettes, early and mature neurons from Control (C1), and RSTS patients 46, 34 and 149. The IF positive staining of the neuroectodermal stem cell markers NESTIN and PAX6, the pan-neuronal cell markers MAP2 (microtubule-associated protein 2) and TUJ1 (beta-III tubulin) and the cortical markers CUX-1 (cut-like homeodomain transcription factor) and TUJ1 can be seen at rosette and early and mature neuron stages on coverslips from the indicated samples. Nuclei are counterstained with DAPI. Differences between C1 and patients and between individual patients are evident at the early neurons stage when the low cell density allows to monitor neuronal morphology. Obj.: Rosettes = 20x; Early (42 days) neurons =40x; Mature neurons = 20x. (PDF 661 kb) [file 12035_2020_1983_MOESM1_ESM.pdf]
