# Supplementary material for: Transcriptome Analysis of iPSC-Derived Neurons from Rubinstein-Taybi Patients Reveals Deficits in Neuronal Differentiation
Source: Mol Neurobiol. 2020 Jun 20;57(9):3685–701. doi: 10.1007/s12035-020-01983-6 (PMC7399686; doi:10.1007/s12035-020-01983-6)

Additional file 2

Visualization of RSTS patients mutations.

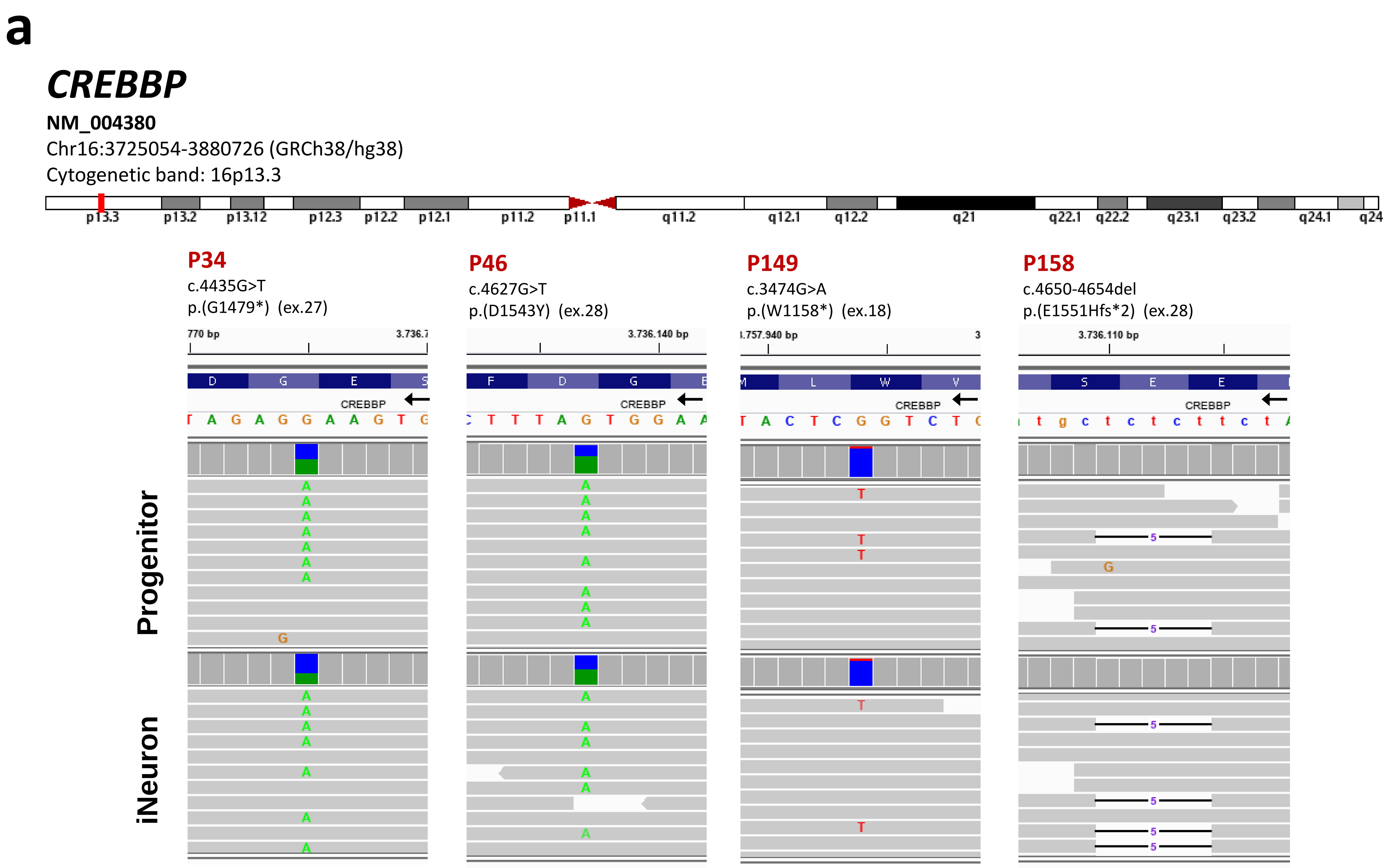

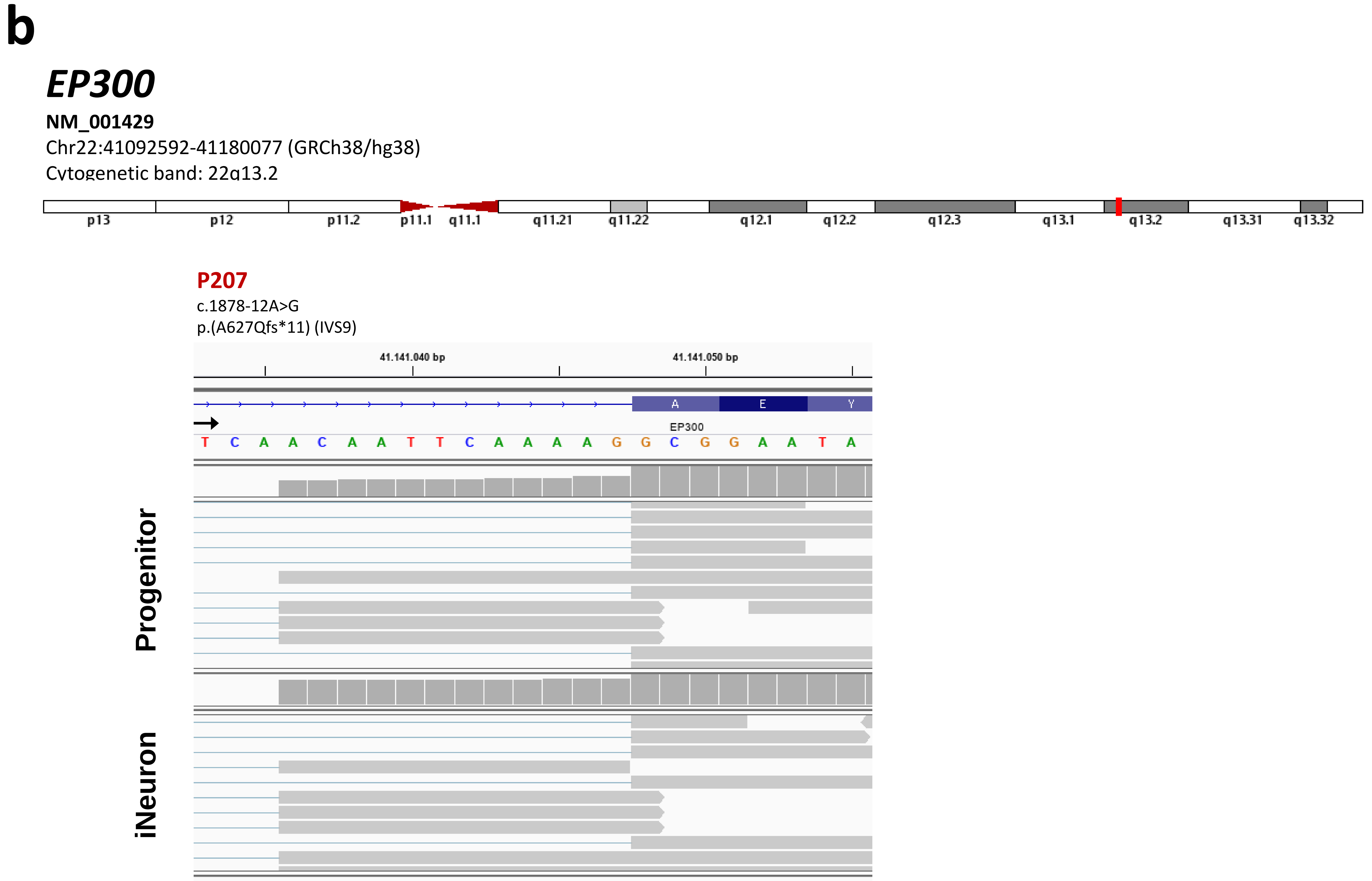

Supplement: Supplementary file 2 — Additional File 2 (Additional_File_2.pdf). Visualization of RSTS patients mutations. IGV (Integrative Genome Viewer) snapshots of reads alignments showing patients specific mutations in CREBBP (top panel – patients P34, P46, P149 and P158) and EP300 (bottom panel – patient P207). Data refer to human assembly GRCh38/hg38. (PDF 285 kb) [file 12035_2020_1983_MOESM2_ESM.pdf]
