# Supplementary figures and images for: Transcriptome Analysis of iPSC-Derived Neurons from Rubinstein-Taybi Patients Reveals Deficits in Neuronal Differentiation
Source: Mol Neurobiol. 2020 Jun 20;57(9):3685–701. doi: 10.1007/s12035-020-01983-6 (PMC7399686; doi:10.1007/s12035-020-01983-6)

# Additional file 3

## Heatmaps of controls and RSTS DEGs.

**a**

**Controls**

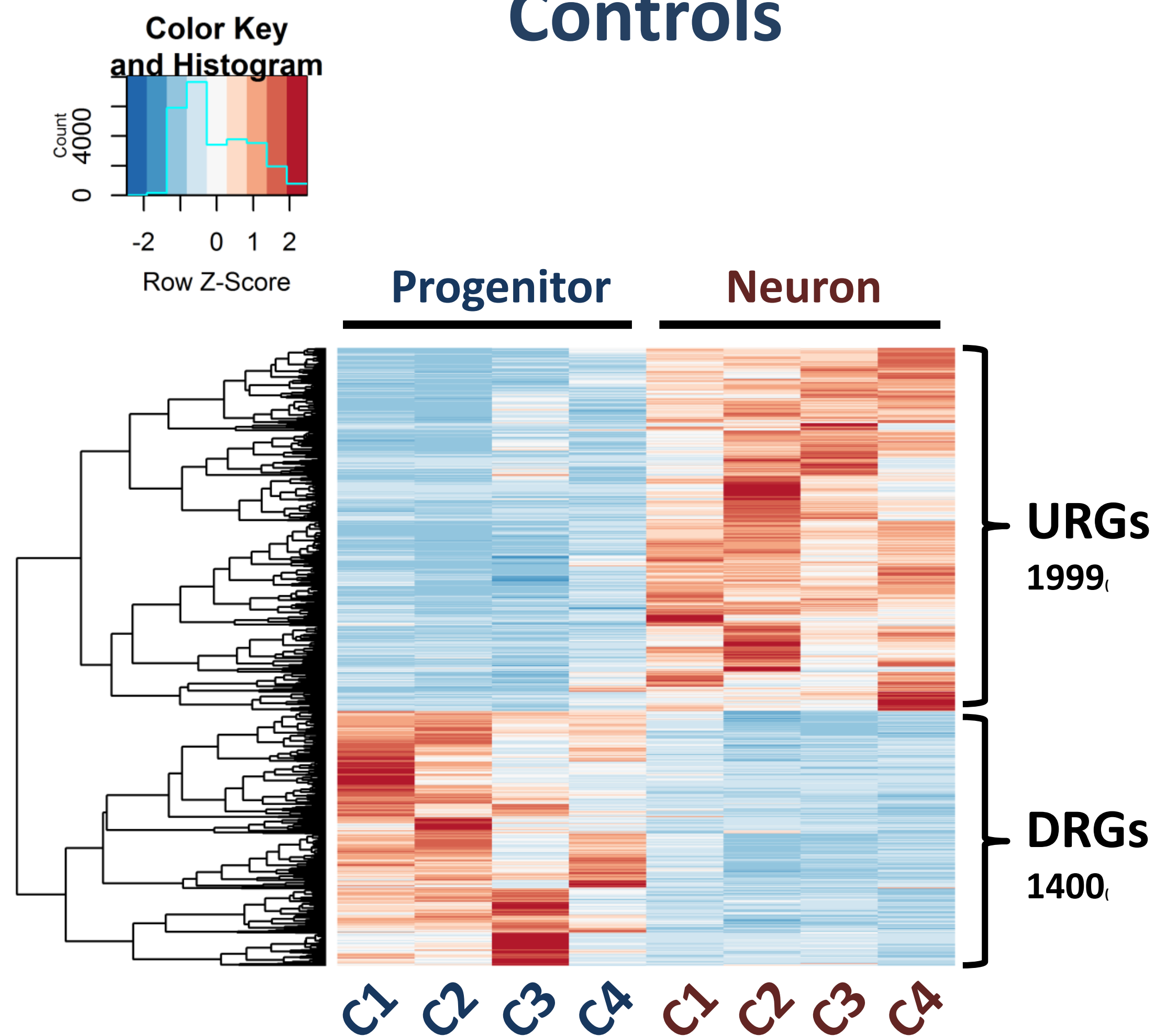

**b**

**RSTS**

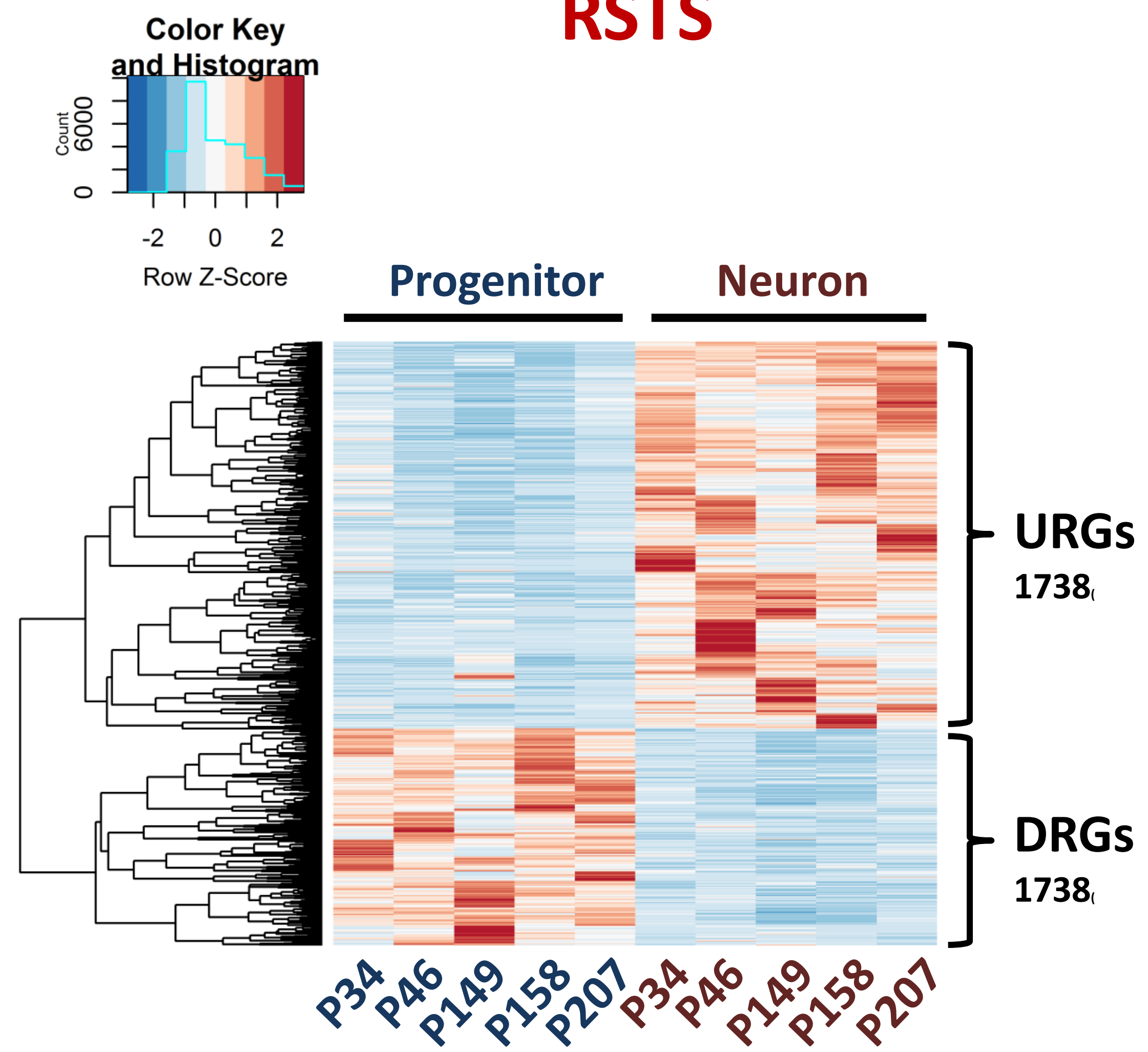

Supplement: Supplementary file 3 — Additional File 3 (Additional_File_3.pdf). Heatmaps of controls and RSTS DEGs. Heatmaps of the expression profile of DEGs at the two time points (iNeurons vs neural progenitors) in (a) controls and (b) RSTS patients, by using 3399 and 2712 DEGs, respectively. Each column represents a sample and each row represents a differentially expressed gene. Gene expression levels were normalized to z-score. Differences in expression are displayed through a color graduation: brown tones represent up-regulation while light blue tones represent down-regulation. Figures were obtained in R environment by using “heatmap.2” function of “gplots” package. (PDF 366 kb) [file 12035_2020_1983_MOESM3_ESM.pdf]
