# Supplementary material for: Transcriptome Analysis of iPSC-Derived Neurons from Rubinstein-Taybi Patients Reveals Deficits in Neuronal Differentiation
Source: Mol Neurobiol. 2020 Jun 20;57(9):3685–701. doi: 10.1007/s12035-020-01983-6 (PMC7399686; doi:10.1007/s12035-020-01983-6)
Supplement: Supplementary file 4 — Additional File 4 (Additional_File_4.pdf). Gene Ontology (GO) enrichment of URGs of controls and RSTS groups. List of significant (padj<0.01) biological processes enriched in controls (n = 279) and RSTS (n = 257) URGs. Group name, GO terms identification codes and relative names are reported in the first three columns, respectively. Additional columns show supplementary information related to GO terms enrichment: in particular, last column refers to the shared/univocal status of the go term between RSTS and controls. (PDF 499 kb) [file 12035_2020_1983_MOESM4_ESM.pdf]

Additional File 4

Gene Ontology (GO) enrichment of URGs of controls and RSTS groups

GROUP	GOID	GOTerm	Nr. Genes	% Associated Genes	Term Adjusted Pvalue	Shared/Univocal
Controls	GO:0099536	synaptic signaling	296	38	1.44E-78	Univocal
Controls	GO:0007268	chemical synaptic transmission	290	38	3.75E-77	Shared
Controls	GO:0007399	nervous system development	574	22	3.36E-56	Shared
Controls	GO:0050804	modulation of chemical synaptic transmission	197	39	2.33E-53	Shared
Controls	GO:0030182	neuron differentiation	381	25	3.14E-45	Shared
Controls	GO:0007267	cell-cell signaling	406	24	5.65E-45	Shared
Controls	GO:0022839	ion gated channel activity	168	39	5.45E-44	Shared
Controls	GO:0048699	generation of neurons	403	24	7.94E-44	Shared
Controls	GO:0050808	synapse organization	179	37	2.18E-42	Shared
Controls	GO:0048666	neuron development	328	26	2.59E-42	Shared
Controls	GO:0022008	neurogenesis	416	23	7.04E-42	Shared
Controls	GO:0015267	channel activity	206	33	1.66E-41	Shared
Controls	GO:0042391	regulation of membrane potential	164	37	1.08E-39	Shared
Controls	GO:0022838	substrate-specific channel activity	195	33	6.63E-39	Shared
Controls	GO:0005261	cation channel activity	168	35	4.58E-36	Shared
Controls	GO:0031175	neuron projection development	288	25	1.54E-34	Shared
Controls	GO:0050877	nervous system process	267	26	2.19E-33	Shared
Controls	GO:0051960	regulation of nervous system development	269	25	5.30E-32	Shared
Controls	GO:0048812	neuron projection morphogenesis	215	28	7.71E-31	Shared
Controls	GO:0015318	inorganic molecular entity transmembrane transporter activity	253	26	5.67E-30	Shared
Controls	GO:0005244	voltage-gated ion channel activity	109	41	9.03E-30	Shared
Controls	GO:0008324	cation transmembrane transporter activity	219	27	6.85E-29	Shared
Controls	GO:0099565	chemical synaptic transmission, postsynaptic	79	50	3.94E-28	Shared
Controls	GO:0032990	cell part morphogenesis	216	27	5.24E-28	Shared
Controls	GO:0045664	regulation of neuron differentiation	210	27	7.68E-28	Shared
Controls	GO:0099504	synaptic vesicle cycle	95	42	7.42E-27	Shared
Controls	GO:0046873	metal ion transmembrane transporter activity	169	30	7.99E-27	Shared
Controls	GO:0006836	neurotransmitter transport	112	38	8.56E-27	Shared
Controls	GO:0060078	regulation of postsynaptic membrane potential	75	50	1.36E-26	Shared
Controls	GO:0048667	cell morphogenesis involved in neuron differentiation	190	28	1.39E-26	Shared
Controls	GO:0043269	regulation of ion transport	191	28	1.59E-26	Shared
Controls	GO:0015075	ion transmembrane transporter activity	257	24	2.41E-26	Shared
Controls	GO:0098655	cation transmembrane transport	236	25	4.85E-26	Shared
Controls	GO:0022857	transmembrane transporter activity	284	23	5.72E-26	Shared
Controls	GO:0007269	neurotransmitter secretion	86	44	1.49E-25	Shared
Controls	GO:0034765	regulation of ion transmembrane transport	151	31	2.01E-25	Shared
Controls	GO:0006812	cation transport	270	23	2.30E-25	Shared
Controls	GO:0050767	regulation of neurogenesis	234	25	2.82E-25	Shared
Controls	GO:0099003	vesicle-mediated transport in synapse	94	40	1.03E-24	Shared
Controls	GO:0050803	regulation of synapse structure or activity	104	37	2.28E-24	Shared
Controls	GO:0050890	cognition	118	34	3.85E-24	Shared
Controls	GO:0097479	synaptic vesicle localization	83	43	4.96E-24	Shared
Controls	GO:0098660	inorganic ion transmembrane transport	221	25	6.64E-24	Shared
Controls	GO:0050807	regulation of synapse organization	101	38	6.80E-24	Shared
Controls	GO:0015276	ligand-gated ion channel activity	91	40	1.94E-23	Shared
Controls	GO:0010975	regulation of neuron projection development	166	28	2.05E-23	Shared
Controls	GO:0034762	regulation of transmembrane transport	164	28	4.36E-23	Shared
Controls	GO:0007611	learning or memory	108	35	8.46E-23	Shared
Controls	GO:0006811	ion transport	336	21	8.67E-23	Shared
Controls	GO:0051049	regulation of transport	370	20	4.07E-22	Shared
Controls	GO:0007215	glutamate receptor signaling pathway	62	51	4.16E-22	Shared
Controls	GO:0048468	cell development	428	19	4.87E-22	Shared
Controls	GO:0034220	ion transmembrane transport	273	22	1.45E-21	Shared
Controls	GO:0048167	regulation of synaptic plasticity	86	39	1.91E-21	Shared
Controls	GO:0030001	metal ion transport	217	24	4.21E-21	Shared
Controls	GO:0098693	regulation of synaptic vesicle cycle	66	47	4.71E-21	Shared

**Controls**	GO:0007409	axonogenesis	153	28	5.70E-21	Shared
**Controls**	GO:0061564	axon development	162	27	1.16E-20	Shared
**Controls**	GO:0017156	calcium ion regulated exocytosis	73	43	1.18E-20	Shared
**Controls**	GO:0120036	plasma membrane bounded cell projection organization	339	20	1.20E-20	Shared
**Controls**	GO:0120035	regulation of plasma membrane bounded cell projection organization	195	25	1.73E-20	Shared
**Controls**	GO:0048489	synaptic vesicle transport	73	42	2.78E-20	Shared
**Controls**	GO:0050806	positive regulation of synaptic transmission	80	40	3.06E-20	Shared
**Controls**	GO:0017158	regulation of calcium ion-dependent exocytosis	61	48	4.83E-20	Shared
**Controls**	GO:0001505	regulation of neurotransmitter levels	119	31	5.68E-20	Shared
**Controls**	GO:0060284	regulation of cell development	239	23	6.08E-20	Shared
**Controls**	GO:0098662	inorganic cation transmembrane transport	197	24	7.94E-20	Shared
**Controls**	GO:0030030	cell projection organization	341	20	1.13E-19	Shared
**Controls**	GO:0016358	dendrite development	98	34	1.27E-19	Shared
**Controls**	GO:0016079	synaptic vesicle exocytosis	63	46	2.85E-19	Shared
**Controls**	GO:1904062	regulation of cation transmembrane transport	114	31	3.09E-19	Shared
**Controls**	GO:0023061	signal release	138	28	4.02E-19	Shared
**Controls**	GO:0007416	synapse assembly	79	39	6.56E-19	Shared
**Controls**	GO:0035235	ionotropic glutamate receptor signaling pathway	50	54	7.42E-19	Shared
**Controls**	GO:0051588	regulation of neurotransmitter transport	66	43	1.32E-18	Shared
**Controls**	GO:0000902	cell morphogenesis	250	22	2.18E-18	Shared
**Controls**	GO:0046928	regulation of neurotransmitter secretion	57	48	2.72E-18	Shared
**Controls**	GO:0004970	ionotropic glutamate receptor activity	48	54	4.25E-18	Shared
**Controls**	GO:0098960	postsynaptic neurotransmitter receptor activity	41	61	5.47E-18	Shared
**Controls**	GO:0007612	learning	70	40	1.37E-17	Shared
**Controls**	GO:0000904	cell morphogenesis involved in differentiation	194	23	2.45E-17	Shared
**Controls**	GO:2001257	regulation of cation channel activity	75	38	2.54E-17	Shared
**Controls**	GO:0055085	transmembrane transport	317	20	2.95E-17	Shared
**Controls**	GO:0051648	vesicle localization	108	31	3.19E-17	Shared
**Controls**	GO:1902803	regulation of synaptic vesicle transport	51	50	3.62E-17	Shared
**Controls**	GO:0051962	positive regulation of nervous system development	159	25	5.33E-17	Shared
**Controls**	GO:0032879	regulation of localization	479	18	5.62E-17	Shared
**Controls**	GO:0035249	synaptic transmission, glutamatergic	51	49	6.27E-17	Shared
**Controls**	GO:0032412	regulation of ion transmembrane transporter activity	94	33	8.30E-17	Shared
**Controls**	GO:2000300	regulation of synaptic vesicle exocytosis	48	51	1.63E-16	Shared
**Controls**	GO:0048731	system development	723	16	1.98E-16	Shared
**Controls**	GO:0032409	regulation of transporter activity	97	31	2.65E-16	Shared
**Controls**	GO:1900449	regulation of glutamate receptor signaling pathway	45	51	1.27E-15	Shared
**Controls**	GO:0099601	regulation of neurotransmitter receptor activity	47	49	2.09E-15	Shared
**Controls**	GO:0032989	cellular component morphogenesis	258	20	2.28E-15	Shared
**Controls**	GO:1903305	regulation of regulated secretory pathway	64	39	2.99E-15	Shared
**Controls**	GO:0007186	G protein-coupled receptor signaling pathway	179	23	5.90E-15	Shared
**Controls**	GO:0017157	regulation of exocytosis	79	34	7.50E-15	Shared
**Controls**	GO:0015077	monovalent inorganic cation transmembrane transporter activity	120	27	7.79E-15	Shared
**Controls**	GO:0051650	establishment of vesicle localization	98	30	2.00E-14	Shared
**Controls**	GO:0007417	central nervous system development	233	21	2.38E-14	Shared
**Controls**	GO:0007275	multicellular organism development	772	15	2.92E-14	Shared
**Controls**	GO:0045666	positive regulation of neuron differentiation	120	26	6.23E-14	Shared
**Controls**	GO:0044057	regulation of system process	148	24	1.64E-13	Shared
**Controls**	GO:0022604	regulation of cell morphogenesis	136	25	1.66E-13	Shared
**Controls**	GO:0099173	postsynapse organization	72	34	2.20E-13	Shared
**Controls**	GO:0048813	dendrite morphogenesis	66	35	2.66E-13	Shared
**Controls**	GO:0071805	potassium ion transmembrane transport	71	33	6.32E-13	Shared
**Controls**	GO:0010769	regulation of cell morphogenesis involved in differentiation	100	28	7.15E-13	Shared
**Controls**	GO:0015079	potassium ion transmembrane transporter activity	67	34	1.84E-12	Shared

**Controls**	GO:0006813	potassium ion transport	76	32	1.87E-12	Shared
**Controls**	GO:0051963	regulation of synapse assembly	49	41	2.65E-12	Shared
**Controls**	GO:0007420	brain development	184	22	2.87E-12	Shared
**Controls**	GO:0004971	AMPA glutamate receptor activity	25	69	4.34E-12	Shared
**Controls**	GO:0050773	regulation of dendrite development	61	35	7.37E-12	Shared
**Controls**	GO:0015672	monovalent inorganic cation transport	130	24	9.14E-12	Shared
**Controls**	GO:0051966	regulation of synaptic transmission, glutamatergic	37	49	1.33E-11	Shared
**Controls**	GO:0099529	neurotransmitter receptor activity involved in regulation of postsynaptic membrane potential	29	59	1.39E-11	Shared
**Controls**	GO:0060079	excitatory postsynaptic potential	45	42	1.52E-11	Shared
**Controls**	GO:0050769	positive regulation of neurogenesis	133	24	2.10E-11	Shared
**Controls**	GO:0060322	head development	187	21	7.43E-11	Shared
**Controls**	GO:0010976	positive regulation of neuron projection development	93	27	1.26E-10	Shared
**Controls**	GO:0099175	regulation of postsynapse organization	48	38	1.28E-10	Shared
**Controls**	GO:0001508	action potential	51	36	2.43E-10	Shared
**Controls**	GO:0007613	memory	52	36	2.89E-10	Shared
**Controls**	GO:0010720	positive regulation of cell development	141	22	3.12E-10	Shared
**Controls**	GO:0050770	regulation of axonogenesis	69	30	6.13E-10	Shared
**Controls**	GO:0051239	regulation of multicellular organismal process	497	16	8.46E-10	Shared
**Controls**	GO:0061337	cardiac conduction	52	35	1.00E-09	Shared
**Controls**	GO:0007411	axon guidance	84	27	1.13E-09	Shared
**Controls**	GO:0060627	regulation of vesicle-mediated transport	128	23	1.36E-09	Shared
**Controls**	GO:0023051	regulation of signaling	569	16	1.57E-09	Shared
**Controls**	GO:0031346	positive regulation of cell projection organization	112	24	1.88E-09	Shared
**Controls**	GO:0015081	sodium ion transmembrane transporter activity	61	31	1.91E-09	Shared
**Controls**	GO:0010646	regulation of cell communication	563	16	2.25E-09	Shared
**Controls**	GO:0051899	membrane depolarization	39	41	2.43E-09	Shared
**Controls**	GO:0007165	signal transduction	765	15	3.28E-09	Shared
**Controls**	GO:0070588	calcium ion transmembrane transport	88	26	3.56E-09	Shared
**Controls**	GO:0051965	positive regulation of synapse assembly	33	45	5.37E-09	Shared
**Controls**	GO:0003015	heart process	82	27	7.10E-09	Shared
**Controls**	GO:0008016	regulation of heart contraction	72	28	7.74E-09	Shared
**Controls**	GO:0015085	calcium ion transmembrane transporter activity	65	30	8.04E-09	Shared
**Controls**	GO:2000311	regulation of AMPA receptor activity	20	67	9.66E-09	Univocal
**Controls**	GO:0035418	protein localization to synapse	39	39	1.14E-08	Shared
**Controls**	GO:1903530	regulation of secretion by cell	153	21	1.62E-08	Shared
**Controls**	GO:0051656	establishment of organelle localization	122	22	1.68E-08	Shared
**Controls**	GO:0048168	regulation of neuronal synaptic plasticity	30	47	1.69E-08	Shared
**Controls**	GO:0051668	localization within membrane	56	31	2.07E-08	Univocal
**Controls**	GO:0060047	heart contraction	79	26	3.25E-08	Univocal
**Controls**	GO:0006816	calcium ion transport	103	23	5.75E-08	Shared
**Controls**	GO:0004972	NMDA glutamate receptor activity	27	48	8.29E-08	Shared
**Controls**	GO:0051046	regulation of secretion	159	20	8.65E-08	Shared
**Controls**	GO:0016247	channel regulator activity	49	32	9.59E-08	Shared
**Controls**	GO:0072511	divalent inorganic cation transport	111	22	1.21E-07	Shared
**Controls**	GO:0010959	regulation of metal ion transport	94	24	1.28E-07	Shared
**Controls**	GO:0098657	import into cell	150	20	1.89E-07	Shared
**Controls**	GO:1903522	regulation of blood circulation	79	25	2.19E-07	Shared
**Controls**	GO:1990138	neuron projection extension	58	29	2.65E-07	Shared
**Controls**	GO:0032594	protein transport within lipid bilayer	29	44	2.68E-07	Univocal
**Controls**	GO:0060341	regulation of cellular localization	189	19	4.45E-07	Shared
**Controls**	GO:0030154	cell differentiation	581	15	4.50E-07	Shared
**Controls**	GO:0060291	long-term synaptic potentiation	38	36	5.70E-07	Shared
**Controls**	GO:2000026	regulation of multicellular organismal development	350	16	5.96E-07	Shared
**Controls**	GO:0051128	regulation of cellular component organization	422	16	6.87E-07	Shared
**Controls**	GO:0006814	sodium ion transport	66	27	6.90E-07	Shared
**Controls**	GO:2000310	regulation of NMDA receptor activity	25	47	7.96E-07	Univocal

**Controls**	GO:0022037	metencephalon development	44	33	7.98E-07	Univocal
**Controls**	GO:0097120	receptor localization to synapse	29	42	9.80E-07	Univocal
**Controls**	GO:0050805	negative regulation of synaptic transmission	31	40	1.33E-06	Shared
**Controls**	GO:0021549	cerebellum development	41	33	1.47E-06	Univocal
**Controls**	GO:0060996	dendritic spine development	41	33	1.47E-06	Shared
**Controls**	GO:0048814	regulation of dendrite morphogenesis	39	34	1.62E-06	Univocal
**Controls**	GO:0051932	synaptic transmission, GABAergic	24	47	1.90E-06	Shared
**Controls**	GO:0051640	organelle localization	153	20	1.94E-06	Univocal
**Controls**	GO:0045595	regulation of cell differentiation	316	17	2.49E-06	Shared
**Controls**	GO:0099637	neurotransmitter receptor transport	25	45	3.39E-06	Univocal
**Controls**	GO:0007214	gamma-aminobutyric acid signaling pathway	16	64	3.49E-06	Shared
**Controls**	GO:0099072	regulation of postsynaptic membrane neurotransmitter receptor levels	32	38	3.55E-06	Univocal
**Controls**	GO:0030900	forebrain development	101	22	4.46E-06	Shared
**Controls**	GO:0060560	developmental growth involved in morphogenesis	72	25	5.16E-06	Shared
**Controls**	GO:0098742	cell-cell adhesion via plasma-membrane adhesion molecules	67	25	5.26E-06	Shared
**Controls**	GO:0030902	hindbrain development	53	28	7.58E-06	Univocal
**Controls**	GO:0031644	regulation of neurological system process	45	30	1.24E-05	Shared
**Controls**	GO:0106027	neuron projection organization	38	32	1.40E-05	Univocal
**Controls**	GO:0099106	ion channel regulator activity	38	32	1.82E-05	Shared
**Controls**	GO:0021953	central nervous system neuron differentiation	59	26	1.84E-05	Shared
**Controls**	GO:0019935	cyclic-nucleotide-mediated signaling	52	27	2.17E-05	Shared
**Controls**	GO:0007188	adenylate cyclase-modulating G protein-coupled receptor signaling pathway	49	28	2.57E-05	Shared
**Controls**	GO:2000463	positive regulation of excitatory postsynaptic potential	17	55	2.97E-05	Univocal
**Controls**	GO:0048588	developmental cell growth	67	24	3.10E-05	Shared
**Controls**	GO:0050794	regulation of cellular process	1212	13	3.14E-05	Shared
**Controls**	GO:0007187	G protein-coupled receptor signaling pathway, coupled to cyclic nucleotide second messenger	52	27	3.18E-05	Shared
**Controls**	GO:0006810	transport	690	14	3.44E-05	Shared
**Controls**	GO:0051961	negative regulation of nervous system development	81	23	3.68E-05	Shared
**Controls**	GO:0015837	amine transport	33	34	3.79E-05	Shared
**Controls**	GO:0060997	dendritic spine morphogenesis	29	37	3.99E-05	Univocal
**Controls**	GO:1905114	cell surface receptor signaling pathway involved in cell-cell signaling	136	19	4.91E-05	Shared
**Controls**	GO:0099084	postsynaptic specialization organization	19	49	5.36E-05	Univocal
**Controls**	GO:1990778	protein localization to cell periphery	82	22	5.44E-05	Shared
**Controls**	GO:0007156	homophilic cell adhesion via plasma membrane adhesion molecules	46	28	6.74E-05	Shared
**Controls**	GO:0098815	modulation of excitatory postsynaptic potential	21	45	6.76E-05	Univocal
**Controls**	GO:0099590	neurotransmitter receptor internalization	16	55	7.09E-05	Univocal
**Controls**	GO:0097061	dendritic spine organization	34	33	7.18E-05	Univocal
**Controls**	GO:0050793	regulation of developmental process	406	15	9.69E-05	Shared
**Controls**	GO:0007616	long-term memory	20	45	1.01E-04	Univocal
**Controls**	GO:0014059	regulation of dopamine secretion	17	52	1.02E-04	Univocal
**Controls**	GO:0051480	regulation of cytosolic calcium ion concentration	76	23	1.03E-04	Shared
**Controls**	GO:0019932	second-messenger-mediated signaling	90	21	1.04E-04	Shared
**Controls**	GO:0003013	circulatory system process	113	20	1.17E-04	Univocal
**Controls**	GO:0019226	transmission of nerve impulse	29	35	1.41E-04	Univocal
**Controls**	GO:0086010	membrane depolarization during action potential	19	46	1.49E-04	Shared
**Controls**	GO:0048172	regulation of short-term neuronal synaptic plasticity	11	73	1.64E-04	Shared
**Controls**	GO:0048791	calcium ion-regulated exocytosis of neurotransmitter	12	67	2.12E-04	Univocal
**Controls**	GO:1903539	protein localization to postsynaptic membrane	22	41	2.41E-04	Univocal
**Controls**	GO:0021537	telencephalon development	71	23	2.52E-04	Shared
**Controls**	GO:0097553	calcium ion transmembrane import into cytosol	43	28	2.64E-04	Shared
**Controls**	GO:0003407	neural retina development	24	38	2.95E-04	Shared

**Controls**	GO:0019933	cAMP-mediated signaling	44	27	3.03E-04	Shared
**Controls**	GO:0099560	synaptic membrane adhesion	15	54	3.23E-04	Shared
**Controls**	GO:0019233	sensory perception of pain	35	30	3.37E-04	Shared
**Controls**	GO:0033555	multicellular organismal response to stress	29	34	3.40E-04	Univocal
**Controls**	GO:0014047	glutamate secretion	22	40	3.55E-04	Shared
**Controls**	GO:0086001	cardiac muscle cell action potential	26	36	4.32E-04	Shared
**Controls**	GO:0097106	postsynaptic density organization	17	47	5.19E-04	Univocal
**Controls**	GO:0060402	calcium ion transport into cytosol	46	26	5.52E-04	Shared
**Controls**	GO:0099068	postsynapse assembly	19	43	5.91E-04	Univocal
**Controls**	GO:0021766	hippocampus development	33	31	6.82E-04	Shared
**Controls**	GO:0031345	negative regulation of cell projection organization	54	24	6.94E-04	Shared
**Controls**	GO:1903169	regulation of calcium ion transmembrane transport	43	27	6.95E-04	Univocal
**Controls**	GO:0009653	anatomical structure morphogenesis	408	15	8.40E-04	Shared
**Controls**	GO:0048675	axon extension	40	28	8.45E-04	Shared
**Controls**	GO:0060074	synapse maturation	17	46	8.49E-04	Shared
**Controls**	GO:0097484	dendrite extension	17	46	8.49E-04	Shared
**Controls**	GO:0099505	regulation of presynaptic membrane potential	8	89	8.56E-04	Univocal
**Controls**	GO:0045665	negative regulation of neuron differentiation	63	23	8.91E-04	Shared
**Controls**	GO:0031646	positive regulation of neurological system process	23	37	9.33E-04	Univocal
**Controls**	GO:0086012	membrane depolarization during cardiac muscle cell action potential	13	57	9.70E-04	Shared
**Controls**	GO:0006897	endocytosis	112	19	0.001141769	Shared
**Controls**	GO:0015800	acidic amino acid transport	27	33	0.001170409	Shared
**Controls**	GO:0055074	calcium ion homeostasis	90	20	0.001263796	Shared
**Controls**	GO:0055082	cellular chemical homeostasis	141	18	0.001280747	Shared
**Controls**	GO:0032386	regulation of intracellular transport	98	20	0.001373172	Univocal
**Controls**	GO:2001259	positive regulation of cation channel activity	25	34	0.001721606	Univocal
**Controls**	GO:0008306	associative learning	30	31	0.001734044	Univocal
**Controls**	GO:0098698	postsynaptic specialization assembly	13	54	0.001873996	Univocal
**Controls**	GO:0015844	monoamine transport	27	33	0.002019871	Univocal
**Controls**	GO:0007618	mating	19	40	0.002020237	Univocal
**Controls**	GO:0008038	neuron recognition	22	37	0.002082274	Shared
**Controls**	GO:0060998	regulation of dendritic spine development	29	31	0.002176393	Univocal
**Controls**	GO:1901019	regulation of calcium ion transmembrane transporter activity	29	31	0.002176393	Univocal
**Controls**	GO:0007600	sensory perception	100	19	0.002204936	Shared
**Controls**	GO:0022603	regulation of anatomical structure morphogenesis	190	17	0.002406792	Univocal
**Controls**	GO:0021761	limbic system development	38	27	0.002523447	Shared
**Controls**	GO:0099150	regulation of postsynaptic specialization assembly	10	67	0.002608909	Univocal
**Controls**	GO:1904889	regulation of excitatory synapse assembly	10	67	0.002608909	Univocal
**Controls**	GO:0044087	regulation of cellular component biogenesis	177	17	0.002637316	Shared
**Controls**	GO:0051968	positive regulation of synaptic transmission, glutamatergic	16	44	0.003224083	Univocal
**Controls**	GO:0021543	pallium development	52	24	0.00333728	Shared
**Controls**	GO:0006874	cellular calcium ion homeostasis	87	20	0.003473067	Shared
**Controls**	GO:0060359	response to ammonium ion	36	27	0.003476127	Shared
**Controls**	GO:0099172	presynapse organization	22	35	0.003941953	Univocal
**Controls**	GO:0046717	acid secretion	33	28	0.004060457	Univocal
**Controls**	GO:0051937	catecholamine transport	24	33	0.004908879	Univocal
**Controls**	GO:0021954	central nervous system neuron development	31	29	0.005410002	Shared
**Controls**	GO:0036465	synaptic vesicle recycling	26	32	0.005477947	Univocal
**Controls**	GO:0060080	inhibitory postsynaptic potential	11	58	0.005544934	Shared
**Controls**	GO:0045921	positive regulation of exocytosis	29	30	0.005639254	Univocal
**Controls**	GO:1900451	positive regulation of glutamate receptor signaling pathway	10	63	0.006158144	Univocal
**Controls**	GO:0006941	striated muscle contraction	45	24	0.006290492	Shared
**Controls**	GO:0072507	divalent inorganic cation homeostasis	93	19	0.00629237	Shared
**Controls**	GO:0001764	neuron migration	46	24	0.006577457	Univocal
**Controls**	GO:0051130	positive regulation of cellular component organization	219	16	0.006595117	Shared

**Controls**	GO:0061387	regulation of extent of cell growth	35	27	0.006610378	Shared
**Controls**	GO:0030073	insulin secretion	51	23	0.006618132	Shared
**Controls**	GO:0050801	ion homeostasis	135	18	0.006784418	Shared
**Controls**	GO:0060048	cardiac muscle contraction	39	26	0.008254102	Shared
**Controls**	GO:0007019	microtubule depolymerization	19	37	0.008561541	Univocal
**Controls**	GO:0140029	exocytic process	26	31	0.009002737	Univocal
**Controls**	GO:0006873	cellular ion homeostasis	115	18	0.009707601	Univocal
**RSTS**	GO:0099537	trans-synaptic signaling	256	34	2.10E-65	Univocal
**RSTS**	GO:0007268	chemical synaptic transmission	254	34	2.23E-65	Shared
**RSTS**	GO:0007399	nervous system development	517	20	5.83E-54	Shared
**RSTS**	GO:0050804	modulation of chemical synaptic transmission	173	35	1.79E-45	Shared
**RSTS**	GO:0007267	cell-cell signaling	365	22	1.52E-42	Shared
**RSTS**	GO:0030182	neuron differentiation	343	23	2.07E-42	Shared
**RSTS**	GO:0048666	neuron development	298	24	9.08E-41	Shared
**RSTS**	GO:0022008	neurogenesis	376	21	2.17E-40	Shared
**RSTS**	GO:0048699	generation of neurons	360	22	2.32E-40	Shared
**RSTS**	GO:0050808	synapse organization	154	32	2.22E-34	Shared
**RSTS**	GO:0015267	channel activity	175	29	5.45E-33	Shared
**RSTS**	GO:0022839	ion gated channel activity	139	33	2.60E-32	Shared
**RSTS**	GO:0031175	neuron projection development	258	23	7.75E-32	Shared
**RSTS**	GO:0022838	substrate-specific channel activity	167	29	1.55E-31	Shared
**RSTS**	GO:0005261	cation channel activity	146	30	7.54E-30	Shared
**RSTS**	GO:0050877	nervous system process	232	23	1.94E-27	Shared
**RSTS**	GO:0042391	regulation of membrane potential	131	30	1.16E-25	Shared
**RSTS**	GO:0048468	cell development	396	18	1.45E-25	Shared
**RSTS**	GO:0048812	neuron projection morphogenesis	187	24	2.60E-25	Shared
**RSTS**	GO:0022890	inorganic cation transmembrane transporter activity	187	24	3.10E-25	Univocal
**RSTS**	GO:0015318	inorganic molecular entity transmembrane transporter activity	221	23	4.16E-25	Shared
**RSTS**	GO:0051960	regulation of nervous system development	230	22	4.48E-25	Shared
**RSTS**	GO:0046873	metal ion transmembrane transporter activity	153	27	1.24E-24	Shared
**RSTS**	GO:0008324	cation transmembrane transporter activity	190	23	3.14E-23	Shared
**RSTS**	GO:0032990	cell part morphogenesis	188	23	5.59E-23	Shared
**RSTS**	GO:0048667	cell morphogenesis involved in neuron differentiation	167	25	1.78E-22	Shared
**RSTS**	GO:0022857	transmembrane transporter activity	250	20	2.08E-22	Shared
**RSTS**	GO:0015075	ion transmembrane transporter activity	224	21	5.69E-22	Shared
**RSTS**	GO:0048731	system development	662	15	5.82E-21	Shared
**RSTS**	GO:0017156	calcium ion regulated exocytosis	69	41	8.74E-21	Shared
**RSTS**	GO:0005244	voltage-gated ion channel activity	89	34	9.51E-21	Shared
**RSTS**	GO:0043269	regulation of ion transport	162	24	1.06E-20	Shared
**RSTS**	GO:0099565	chemical synaptic transmission, postsynaptic	65	42	1.38E-20	Shared
**RSTS**	GO:0045664	regulation of neuron differentiation	177	23	1.56E-20	Shared
**RSTS**	GO:0099504	synaptic vesicle cycle	81	36	1.57E-20	Shared
**RSTS**	GO:0007409	axonogenesis	140	26	1.64E-20	Shared
**RSTS**	GO:0048167	regulation of synaptic plasticity	78	36	4.50E-20	Shared
**RSTS**	GO:0061564	axon development	147	25	9.12E-20	Shared
**RSTS**	GO:0010975	regulation of neuron projection development	145	25	1.57E-19	Shared
**RSTS**	GO:0098655	cation transmembrane transport	201	21	2.53E-19	Shared
**RSTS**	GO:0007269	neurotransmitter secretion	73	37	3.96E-19	Shared
**RSTS**	GO:0007275	multicellular organism development	707	14	4.23E-19	Shared
**RSTS**	GO:0006811	ion transport	293	18	4.73E-19	Shared
**RSTS**	GO:0098660	inorganic ion transmembrane transport	191	22	5.81E-19	Shared
**RSTS**	GO:0006836	neurotransmitter transport	93	31	6.37E-19	Shared
**RSTS**	GO:0120036	plasma membrane bounded cell projection organization	302	18	6.40E-19	Shared
**RSTS**	GO:0060078	regulation of postsynaptic membrane potential	61	42	6.51E-19	Shared
**RSTS**	GO:0032879	regulation of localization	437	16	7.18E-19	Shared
**RSTS**	GO:0120035	regulation of plasma membrane bounded cell projection organization	174	22	1.50E-18	Shared
**RSTS**	GO:0030030	cell projection organization	305	18	2.03E-18	Shared
**RSTS**	GO:0034765	regulation of ion transmembrane transport	126	26	2.06E-18	Shared

RSTS	GO:0006812	cation transport	229	20	2.38E-18	Shared
RSTS	GO:0050767	regulation of neurogenesis	196	21	3.76E-18	Shared
RSTS	GO:0051049	regulation of transport	321	18	5.56E-18	Shared
RSTS	GO:0017158	regulation of calcium ion-dependent exocytosis	55	44	6.11E-18	Shared
RSTS	GO:0030001	metal ion transport	190	21	1.41E-17	Shared
RSTS	GO:0050806	positive regulation of synaptic transmission	70	36	3.34E-17	Shared
RSTS	GO:0097479	synaptic vesicle localization	69	36	4.25E-17	Shared
RSTS	GO:0015276	ligand-gated ion channel activity	75	33	7.97E-17	Shared
RSTS	GO:0034762	regulation of transmembrane transport	137	24	1.45E-16	Shared
RSTS	GO:0007416	synapse assembly	70	35	1.67E-16	Shared
RSTS	GO:0034220	ion transmembrane transport	234	19	1.71E-16	Shared
RSTS	GO:0001505	regulation of neurotransmitter levels	103	27	1.81E-16	Shared
RSTS	GO:0050890	cognition	96	28	2.24E-16	Shared
RSTS	GO:0048489	synaptic vesicle transport	63	36	6.70E-16	Shared
RSTS	GO:0050803	regulation of synapse structure or activity	83	30	9.64E-16	Shared
RSTS	GO:0098662	inorganic cation transmembrane transport	171	21	1.13E-15	Shared
RSTS	GO:0000902	cell morphogenesis	220	19	1.19E-15	Shared
RSTS	GO:0099003	vesicle-mediated transport in synapse	75	32	1.41E-15	Shared
RSTS	GO:0016079	synaptic vesicle exocytosis	55	40	1.54E-15	Shared
RSTS	GO:0060284	regulation of cell development	204	20	2.30E-15	Shared
RSTS	GO:0007215	glutamate receptor signaling pathway	50	42	4.50E-15	Shared
RSTS	GO:0007611	learning or memory	87	29	5.89E-15	Shared
RSTS	GO:0000904	cell morphogenesis involved in differentiation	171	21	7.87E-15	Shared
RSTS	GO:0023061	signal release	118	24	1.19E-14	Shared
RSTS	GO:0050807	regulation of synapse organization	79	30	1.56E-14	Shared
RSTS	GO:0051962	positive regulation of nervous system development	140	22	1.57E-14	Shared
RSTS	GO:0032989	cellular component morphogenesis	231	19	1.72E-14	Shared
RSTS	GO:0098693	regulation of synaptic vesicle cycle	54	38	1.81E-14	Shared
RSTS	GO:0098960	postsynaptic neurotransmitter receptor activity	35	54	2.64E-14	Shared
RSTS	GO:0007417	central nervous system development	208	19	2.34E-13	Shared
RSTS	GO:1903305	regulation of regulated secretory pathway	57	35	2.92E-13	Shared
RSTS	GO:0051239	regulation of multicellular organismal process	460	15	2.95E-13	Shared
RSTS	GO:0071805	potassium ion transmembrane transport	67	31	3.19E-13	Shared
RSTS	GO:0007612	learning	59	34	3.25E-13	Shared
RSTS	GO:0051588	regulation of neurotransmitter transport	55	36	3.45E-13	Shared
RSTS	GO:0055085	transmembrane transport	273	17	4.27E-13	Shared
RSTS	GO:0007186	G protein-coupled receptor signaling pathway	156	21	6.05E-13	Shared
RSTS	GO:0023051	regulation of signaling	523	14	8.38E-13	Shared
RSTS	GO:0044057	regulation of system process	132	22	1.40E-12	Shared
RSTS	GO:0015079	potassium ion transmembrane transporter activity	63	32	1.84E-12	Shared
RSTS	GO:0006813	potassium ion transport	71	29	1.87E-12	Shared
RSTS	GO:0007165	signal transduction	696	14	1.94E-12	Shared
RSTS	GO:0035235	ionotropic glutamate receptor signaling pathway	40	43	1.98E-12	Shared
RSTS	GO:0007411	axon guidance	83	27	2.20E-12	Shared
RSTS	GO:0017157	regulation of exocytosis	69	30	2.20E-12	Shared
RSTS	GO:0010646	regulation of cell communication	516	14	2.52E-12	Shared
RSTS	GO:0003015	heart process	82	27	3.10E-12	Shared
RSTS	GO:0061337	cardiac conduction	53	35	3.95E-12	Shared
RSTS	GO:0035249	synaptic transmission, glutamatergic	42	41	6.06E-12	Shared
RSTS	GO:1902803	regulation of synaptic vesicle transport	42	41	6.06E-12	Shared
RSTS	GO:2000300	regulation of synaptic vesicle exocytosis	40	42	7.53E-12	Shared
RSTS	GO:0008016	regulation of heart contraction	72	28	1.35E-11	Shared
RSTS	GO:0004970	ionotropic glutamate receptor activity	38	43	1.36E-11	Shared
RSTS	GO:1904062	regulation of cation transmembrane transport	91	25	1.62E-11	Shared
RSTS	GO:0030154	cell differentiation	538	14	3.24E-11	Shared
RSTS	GO:0015077	monovalent inorganic cation transmembrane transporter activity	103	23	4.30E-11	Shared
RSTS	GO:0046928	regulation of neurotransmitter secretion	45	37	5.07E-11	Shared
RSTS	GO:1903522	regulation of blood circulation	80	26	5.51E-11	Shared

**RSTS**	GO:0007420	brain development	163	19	7.67E-11	Shared
**RSTS**	GO:0045666	positive regulation of neuron differentiation	103	23	1.46E-10	Shared
**RSTS**	GO:0099529	neurotransmitter receptor activity involved in regulation of postsynaptic membrane potential	26	55	1.74E-10	Shared
**RSTS**	GO:0051648	vesicle localization	87	25	1.86E-10	Shared
**RSTS**	GO:1990138	neuron projection extension	59	30	2.38E-10	Shared
**RSTS**	GO:0010976	positive regulation of neuron projection development	85	25	3.13E-10	Shared
**RSTS**	GO:0098742	cell-cell adhesion via plasma-membrane adhesion molecules	70	27	3.47E-10	Shared
**RSTS**	GO:0060322	head development	167	19	4.87E-10	Shared
**RSTS**	GO:0016358	dendrite development	74	26	5.29E-10	Shared
**RSTS**	GO:0031346	positive regulation of cell projection organization	104	22	6.02E-10	Shared
**RSTS**	GO:0032412	regulation of ion transmembrane transporter activity	74	26	6.40E-10	Shared
**RSTS**	GO:0032409	regulation of transporter activity	77	25	7.02E-10	Shared
**RSTS**	GO:0051963	regulation of synapse assembly	42	36	1.08E-09	Shared
**RSTS**	GO:0050770	regulation of axonogenesis	63	28	1.29E-09	Shared
**RSTS**	GO:0008038	neuron recognition	28	48	1.70E-09	Shared
**RSTS**	GO:0051650	establishment of vesicle localization	81	24	1.76E-09	Shared
**RSTS**	GO:2001257	regulation of cation channel activity	57	29	1.92E-09	Shared
**RSTS**	GO:0060560	developmental growth involved in morphogenesis	73	25	2.79E-09	Shared
**RSTS**	GO:0015085	calcium ion transmembrane transporter activity	61	28	3.12E-09	Shared
**RSTS**	GO:0015672	monovalent inorganic cation transport	113	21	3.69E-09	Shared
**RSTS**	GO:0010769	regulation of cell morphogenesis involved in differentiation	84	24	3.88E-09	Shared
**RSTS**	GO:0060627	regulation of vesicle-mediated transport	115	21	5.19E-09	Shared
**RSTS**	GO:0001508	action potential	46	32	5.83E-09	Shared
**RSTS**	GO:0048588	developmental cell growth	69	26	6.31E-09	Shared
**RSTS**	GO:1900449	regulation of glutamate receptor signaling pathway	34	40	7.67E-09	Shared
**RSTS**	GO:0008015	blood circulation	113	21	8.84E-09	Univocal
**RSTS**	GO:0022604	regulation of cell morphogenesis	112	21	1.23E-08	Shared
**RSTS**	GO:0050769	positive regulation of neurogenesis	114	20	1.33E-08	Shared
**RSTS**	GO:2000026	regulation of multicellular organismal development	319	15	3.14E-08	Shared
**RSTS**	GO:0010720	positive regulation of cell development	122	20	4.10E-08	Shared
**RSTS**	GO:0007156	homophilic cell adhesion via plasma membrane adhesion molecules	48	30	4.40E-08	Shared
**RSTS**	GO:0051965	positive regulation of synapse assembly	30	41	4.47E-08	Shared
**RSTS**	GO:0099601	regulation of neurotransmitter receptor activity	35	36	5.66E-08	Shared
**RSTS**	GO:0015081	sodium ion transmembrane transporter activity	54	28	7.70E-08	Shared
**RSTS**	GO:0051966	regulation of synaptic transmission, glutamatergic	30	40	1.01E-07	Shared
**RSTS**	GO:0048168	regulation of neuronal synaptic plasticity	27	43	1.41E-07	Shared
**RSTS**	GO:0070588	calcium ion transmembrane transport	77	23	2.35E-07	Shared
**RSTS**	GO:0098657	import into cell	134	18	5.31E-07	Shared
**RSTS**	GO:0051128	regulation of cellular component organization	377	14	5.66E-07	Shared
**RSTS**	GO:0099173	postsynapse organization	55	26	6.29E-07	Shared
**RSTS**	GO:0016247	channel regulator activity	44	29	6.75E-07	Shared
**RSTS**	GO:0006816	calcium ion transport	91	21	7.72E-07	Shared
**RSTS**	GO:0072511	divalent inorganic cation transport	99	20	8.27E-07	Shared
**RSTS**	GO:0006935	chemotaxis	118	19	1.06E-06	Univocal
**RSTS**	GO:0048675	axon extension	42	29	1.63E-06	Shared
**RSTS**	GO:0051961	negative regulation of nervous system development	77	22	1.75E-06	Shared
**RSTS**	GO:0007613	memory	41	29	3.61E-06	Shared
**RSTS**	GO:0010959	regulation of metal ion transport	82	21	3.64E-06	Shared
**RSTS**	GO:0045595	regulation of cell differentiation	281	15	3.89E-06	Shared
**RSTS**	GO:0048813	dendrite morphogenesis	49	26	4.84E-06	Shared

RSTS	GO:0007187	G protein-coupled receptor signaling pathway, coupled to cyclic nucleotide second messenger	49	26	5.88E-06	Shared
RSTS	GO:0007188	adenylate cyclase-modulating G protein-coupled receptor signaling pathway	46	27	6.04E-06	Shared
RSTS	GO:0009653	anatomical structure morphogenesis	374	14	6.14E-06	Shared
RSTS	GO:0006814	sodium ion transport	59	24	6.52E-06	Shared
RSTS	GO:1903530	regulation of secretion by cell	129	18	7.50E-06	Shared
RSTS	GO:0030900	forebrain development	92	20	7.61E-06	Shared
RSTS	GO:0007413	axonal fasciculation	17	53	8.49E-06	Univocal
RSTS	GO:0060291	long-term synaptic potentiation	33	32	9.61E-06	Shared
RSTS	GO:0051046	regulation of secretion	136	18	9.75E-06	Shared
RSTS	GO:0050793	regulation of developmental process	367	14	1.16E-05	Shared
RSTS	GO:0060079	excitatory postsynaptic potential	33	32	1.26E-05	Shared
RSTS	GO:0051899	membrane depolarization	31	33	1.29E-05	Shared
RSTS	GO:0050768	negative regulation of neurogenesis	71	21	2.18E-05	Univocal
RSTS	GO:1905114	cell surface receptor signaling pathway involved in cell-cell signaling	124	18	2.19E-05	Shared
RSTS	GO:0019932	second-messenger-mediated signaling	83	20	2.34E-05	Shared
RSTS	GO:0014047	glutamate secretion	22	41	2.57E-05	Shared
RSTS	GO:0006810	transport	613	13	3.35E-05	Shared
RSTS	GO:0019935	cyclic-nucleotide-mediated signaling	47	25	3.55E-05	Shared
RSTS	GO:0019933	cAMP-mediated signaling	42	27	4.26E-05	Shared
RSTS	GO:0050794	regulation of cellular process	1068	12	5.89E-05	Shared
RSTS	GO:0021537	telencephalon development	67	21	6.23E-05	Shared
RSTS	GO:0015800	acidic amino acid transport	27	34	7.01E-05	Shared
RSTS	GO:0045665	negative regulation of neuron differentiation	60	22	7.13E-05	Shared
RSTS	GO:0044087	regulation of cellular component biogenesis	166	16	8.03E-05	Shared
RSTS	GO:0021953	central nervous system neuron differentiation	53	23	8.04E-05	Shared
RSTS	GO:0004971	AMPA glutamate receptor activity	17	47	8.38E-05	Shared
RSTS	GO:0099106	ion channel regulator activity	34	29	8.84E-05	Shared
RSTS	GO:0050773	regulation of dendrite development	44	25	1.18E-04	Shared
RSTS	GO:0060341	regulation of cellular localization	161	16	1.51E-04	Shared
RSTS	GO:0098772	molecular function regulator	248	15	1.68E-04	Univocal
RSTS	GO:0006941	striated muscle contraction	44	25	1.69E-04	Shared
RSTS	GO:0051932	synaptic transmission, GABAergic	20	40	1.70E-04	Shared
RSTS	GO:0060048	cardiac muscle contraction	39	26	1.74E-04	Shared
RSTS	GO:0007600	sensory perception	94	19	1.75E-04	Shared
RSTS	GO:0003407	neural retina development	23	36	1.93E-04	Shared
RSTS	GO:0086012	membrane depolarization during cardiac muscle cell action potential	13	57	2.24E-04	Shared
RSTS	GO:0060041	retina development in camera-type eye	41	25	2.28E-04	Univocal
RSTS	GO:1990778	protein localization to cell periphery	73	20	2.74E-04	Shared
RSTS	GO:0050771	negative regulation of axonogenesis	27	32	2.91E-04	Univocal
RSTS	GO:0051656	establishment of organelle localization	99	18	2.91E-04	Shared
RSTS	GO:0051130	positive regulation of cellular component organization	203	15	3.10E-04	Shared
RSTS	GO:0050805	negative regulation of synaptic transmission	25	33	3.94E-04	Shared
RSTS	GO:0086001	cardiac muscle cell action potential	25	33	3.94E-04	Shared
RSTS	GO:0007166	cell surface receptor signaling pathway	386	13	3.96E-04	Univocal
RSTS	GO:0051094	positive regulation of developmental process	215	15	4.37E-04	Univocal
RSTS	GO:0098609	cell-cell adhesion	133	17	4.60E-04	Univocal
RSTS	GO:0086003	cardiac muscle cell contraction	24	33	5.21E-04	Univocal
RSTS	GO:0021543	pallium development	50	23	5.47E-04	Shared
RSTS	GO:0060074	synapse maturation	16	44	5.99E-04	Shared
RSTS	GO:0015837	amine transport	28	30	6.03E-04	Shared
RSTS	GO:0061387	regulation of extent of cell growth	34	27	6.29E-04	Shared
RSTS	GO:0031345	negative regulation of cell projection organization	49	23	6.59E-04	Shared
RSTS	GO:0035418	protein localization to synapse	29	29	7.10E-04	Shared
RSTS	GO:0048172	regulation of short-term neuronal synaptic plasticity	10	67	8.07E-04	Shared
RSTS	GO:0007214	gamma-aminobutyric acid signaling pathway	13	52	8.19E-04	Shared
RSTS	GO:0010721	negative regulation of cell development	73	19	8.20E-04	Univocal

**RSTS**	GO:0010771	negative regulation of cell morphogenesis involved in differentiation	31	28	9.11E-04	Univocal
**RSTS**	GO:0004972	NMDA glutamate receptor activity	20	36	0.001058839	Shared
**RSTS**	GO:0046530	photoreceptor cell differentiation	20	36	0.001058839	Univocal
**RSTS**	GO:0051480	regulation of cytosolic calcium ion concentration	65	20	0.001068576	Shared
**RSTS**	GO:0021954	central nervous system neuron development	30	28	0.001268758	Shared
**RSTS**	GO:0086010	membrane depolarization during action potential	17	40	0.001298023	Shared
**RSTS**	GO:0021766	hippocampus development	30	28	0.001576456	Shared
**RSTS**	GO:0031644	regulation of neurological system process	37	25	0.001820823	Shared
**RSTS**	GO:0055082	cellular chemical homeostasis	125	16	0.00183348	Shared
**RSTS**	GO:0006936	muscle contraction	67	19	0.002178679	Univocal
**RSTS**	GO:0086065	cell communication involved in cardiac conduction	22	33	0.002181972	Univocal
**RSTS**	GO:0010977	negative regulation of neuron projection development	42	23	0.002400545	Univocal
**RSTS**	GO:0048878	chemical homeostasis	167	15	0.002401344	Univocal
**RSTS**	GO:0050801	ion homeostasis	122	16	0.002734827	Shared
**RSTS**	GO:0021761	limbic system development	35	25	0.003011531	Shared
**RSTS**	GO:0072507	divalent inorganic cation homeostasis	84	18	0.003128577	Shared
**RSTS**	GO:0004930	G protein-coupled receptor activity	60	20	0.003229763	Univocal
**RSTS**	GO:0050433	regulation of catecholamine secretion	18	37	0.003289644	Univocal
**RSTS**	GO:0055074	calcium ion homeostasis	79	18	0.003321876	Shared
**RSTS**	GO:0019233	sensory perception of pain	30	27	0.003622225	Shared
**RSTS**	GO:0097484	dendrite extension	15	42	0.003826203	Shared
**RSTS**	GO:0099175	regulation of postsynapse organization	32	26	0.004011108	Shared
**RSTS**	GO:0045956	positive regulation of calcium ion-dependent exocytosis	14	44	0.004179026	Univocal
**RSTS**	GO:0099560	synaptic membrane adhesion	13	46	0.004274112	Shared
**RSTS**	GO:0051240	positive regulation of multicellular organismal process	251	14	0.004446732	Univocal
**RSTS**	GO:0051489	regulation of filopodium assembly	18	36	0.004607984	Univocal
**RSTS**	GO:0072503	cellular divalent inorganic cation homeostasis	81	18	0.005095197	Univocal
**RSTS**	GO:0006874	cellular calcium ion homeostasis	77	18	0.005366318	Shared
**RSTS**	GO:0006897	endocytosis	98	17	0.006021121	Shared
**RSTS**	GO:0007193	adenylate cyclase-inhibiting G protein-coupled receptor signaling pathway	22	31	0.006481649	Univocal
**RSTS**	GO:0006835	dicarboxylic acid transport	30	26	0.006529879	Univocal
**RSTS**	GO:0098659	inorganic cation import across plasma membrane	25	29	0.006569615	Univocal
**RSTS**	GO:0046847	filopodium assembly	21	32	0.006725349	Univocal
**RSTS**	GO:0060402	calcium ion transport into cytosol	39	23	0.007644665	Shared
**RSTS**	GO:0097553	calcium ion transmembrane import into cytosol	36	24	0.008259884	Shared
**RSTS**	GO:0060359	response to ammonium ion	32	25	0.008327986	Shared
**RSTS**	GO:0060080	inhibitory postsynaptic potential	10	56	0.00850694	Shared
**RSTS**	GO:1902287	semaphorin-plexin signaling pathway involved in axon guidance	10	56	0.00850694	Univocal
**RSTS**	GO:0021879	forebrain neuron differentiation	21	31	0.008788968	Univocal
**RSTS**	GO:0043523	regulation of neuron apoptotic process	50	21	0.009066054	Univocal
**RSTS**	GO:0030073	insulin secretion	46	21	0.009330637	Shared
**RSTS**	GO:0060996	dendritic spine development	31	25	0.009771876	Shared
