# Supplementary material for: Transcriptome Analysis of iPSC-Derived Neurons from Rubinstein-Taybi Patients Reveals Deficits in Neuronal Differentiation
Source: Mol Neurobiol. 2020 Jun 20;57(9):3685–701. doi: 10.1007/s12035-020-01983-6 (PMC7399686; doi:10.1007/s12035-020-01983-6)
Supplement: Supplementary file 5 — Additional File 5 (Additional_File_5.pdf). Gene Ontology (GO) enrichment of DRGs of controls and RSTS groups. List of significant (padj<0.01) biological processes enriched in controls (n = 135) and RSTS (n = 155) DRGs. Group name, GO terms identification codes and relative names are reported in the first three columns, respectively. Additional columns show supplementary information related to GO terms enrichment: in particular, last column refers to the shared/univocal status of the GO term between RSTS and controls. (PDF 347 kb) [file 12035_2020_1983_MOESM5_ESM.pdf]

Additional File 5

Gene Ontology (GO) enrichment of DRGs of controls and RSTS groups

GROUP	GOID	GOTerm	Nr. Genes	% Associated Genes	Term Adjusted Pvalue	Shared/Univocal
**Controls**	GO:0000278	mitotic cell cycle	232	20	1.81E-34	Shared
**Controls**	GO:1903047	mitotic cell cycle process	208	20	8.10E-33	Shared
**Controls**	GO:0022402	cell cycle process	256	17	3.72E-27	Shared
**Controls**	GO:0000280	nuclear division	115	25	4.98E-24	Shared
**Controls**	GO:0048285	organelle fission	118	23	5.56E-22	Shared
**Controls**	GO:0006260	DNA replication	93	27	1.22E-21	Shared
**Controls**	GO:0006261	DNA-dependent DNA replication	66	34	4.37E-21	Shared
**Controls**	GO:0140014	mitotic nuclear division	91	27	4.51E-21	Shared
**Controls**	GO:0044770	cell cycle phase transition	142	20	1.86E-19	Shared
**Controls**	GO:0044772	mitotic cell cycle phase transition	136	20	2.54E-19	Shared
**Controls**	GO:0006259	DNA metabolic process	182	16	1.26E-16	Shared
**Controls**	GO:0007346	regulation of mitotic cell cycle	144	18	1.56E-16	Shared
**Controls**	GO:0010564	regulation of cell cycle process	157	17	2.74E-15	Shared
**Controls**	GO:0051726	regulation of cell cycle	208	15	9.66E-15	Shared
**Controls**	GO:0051783	regulation of nuclear division	66	26	5.03E-14	Univocal
**Controls**	GO:0098813	nuclear chromosome segregation	71	25	6.74E-14	Shared
**Controls**	GO:0007088	regulation of mitotic nuclear division	61	27	1.82E-13	Shared
**Controls**	GO:0000819	sister chromatid segregation	59	27	6.51E-13	Shared
**Controls**	GO:0000070	mitotic sister chromatid segregation	52	29	1.73E-12	Shared
**Controls**	GO:0007093	mitotic cell cycle checkpoint	56	27	3.12E-12	Shared
**Controls**	GO:0044843	cell cycle G1/S phase transition	73	23	3.69E-12	Shared
**Controls**	GO:0000082	G1/S transition of mitotic cell cycle	70	23	4.31E-12	Shared
**Controls**	GO:0090068	positive regulation of cell cycle process	76	22	1.34E-11	Shared
**Controls**	GO:0045787	positive regulation of cell cycle	92	19	1.94E-11	Shared
**Controls**	GO:0000075	cell cycle checkpoint	65	24	1.98E-11	Shared
**Controls**	GO:0045930	negative regulation of mitotic cell cycle	81	21	3.09E-11	Shared
**Controls**	GO:0007010	cytoskeleton organization	198	14	3.49E-11	Shared
**Controls**	GO:0051276	chromosome organization	186	14	4.40E-11	Shared
**Controls**	GO:0071103	DNA conformation change	67	23	6.00E-11	Shared
**Controls**	GO:0000226	microtubule cytoskeleton organization	108	18	9.19E-11	Shared
**Controls**	GO:0044786	cell cycle DNA replication	32	38	1.11E-10	Shared
**Controls**	GO:0006974	cellular response to DNA damage stimulus	149	15	1.33E-10	Shared
**Controls**	GO:1901987	regulation of cell cycle phase transition	100	18	2.31E-10	Shared
**Controls**	GO:1901990	regulation of mitotic cell cycle phase transition	95	18	4.26E-10	Shared
**Controls**	GO:0033260	nuclear DNA replication	28	39	1.55E-09	Shared
**Controls**	GO:0051983	regulation of chromosome segregation	37	31	2.44E-09	Shared
**Controls**	GO:0006323	DNA packaging	47	26	2.56E-09	Shared
**Controls**	GO:0051321	meiotic cell cycle	55	23	9.08E-09	Shared
**Controls**	GO:1901988	negative regulation of cell cycle phase transition	64	21	1.36E-08	Shared
**Controls**	GO:0006281	DNA repair	104	17	1.36E-08	Shared
**Controls**	GO:0010948	negative regulation of cell cycle process	79	18	3.86E-08	Shared
**Controls**	GO:0006996	organelle organization	427	11	7.67E-08	Shared
**Controls**	GO:0031570	DNA integrity checkpoint	48	23	9.84E-08	Shared
**Controls**	GO:0006270	DNA replication initiation	20	45	1.36E-07	Shared
**Controls**	GO:1901991	negative regulation of mitotic cell cycle phase transition	59	21	1.45E-07	Shared
**Controls**	GO:1903046	meiotic cell cycle process	44	24	1.76E-07	Shared
**Controls**	GO:0006275	regulation of DNA replication	37	26	4.67E-07	Shared
**Controls**	GO:0006310	DNA recombination	60	20	7.33E-07	Shared
**Controls**	GO:0007051	spindle organization	44	23	1.12E-06	Shared
**Controls**	GO:0033045	regulation of sister chromatid segregation	30	29	1.21E-06	Shared
**Controls**	GO:0042127	regulation of cell population proliferation	208	12	1.26E-06	Univocal
**Controls**	GO:0042770	signal transduction in response to DNA damage	41	24	1.48E-06	Shared
**Controls**	GO:0045786	negative regulation of cell cycle	111	15	2.29E-06	Shared
**Controls**	GO:0044774	mitotic DNA integrity checkpoint	35	26	2.53E-06	Shared
**Controls**	GO:0090329	regulation of DNA-dependent DNA replication	24	34	2.82E-06	Shared

**Controls**	GO:0008284	positive regulation of cell population proliferation	130	14	4.10E-06	Univocal
**Controls**	GO:0140013	meiotic nuclear division	39	23	5.04E-06	Shared
**Controls**	GO:0044839	cell cycle G2/M phase transition	62	18	5.25E-06	Shared
**Controls**	GO:0051052	regulation of DNA metabolic process	80	16	5.49E-06	Shared
**Controls**	GO:0003006	developmental process involved in reproduction	101	15	6.25E-06	Univocal
**Controls**	GO:0031100	animal organ regeneration	28	28	1.19E-05	Univocal
**Controls**	GO:0051784	negative regulation of nuclear division	25	30	1.91E-05	Univocal
**Controls**	GO:0035239	tube morphogenesis	122	14	2.21E-05	Univocal
**Controls**	GO:0006302	double-strand break repair	51	19	2.30E-05	Shared
**Controls**	GO:1902850	microtubule cytoskeleton organization involved in mitosis	36	23	2.38E-05	Shared
**Controls**	GO:0006333	chromatin assembly or disassembly	36	23	3.42E-05	Shared
**Controls**	GO:0033046	negative regulation of sister chromatid segregation	21	33	4.01E-05	Shared
**Controls**	GO:0007098	centrosome cycle	37	23	4.11E-05	Shared
**Controls**	GO:0035295	tube development	142	13	4.67E-05	Univocal
**Controls**	GO:0033554	cellular response to stress	256	11	4.72E-05	Shared
**Controls**	GO:0045005	DNA-dependent DNA replication maintenance of fidelity	19	36	4.91E-05	Shared
**Controls**	GO:0006271	DNA strand elongation involved in DNA replication	11	61	5.42E-05	Shared
**Controls**	GO:0065004	protein-DNA complex assembly	47	20	5.50E-05	Shared
**Controls**	GO:0007052	mitotic spindle organization	31	25	5.69E-05	Shared
**Controls**	GO:0044773	mitotic DNA damage checkpoint	31	25	5.69E-05	Shared
**Controls**	GO:0072422	signal transduction involved in DNA damage checkpoint	26	28	6.88E-05	Shared
**Controls**	GO:0006334	nucleosome assembly	29	26	7.04E-05	Shared
**Controls**	GO:0000724	double-strand break repair via homologous recombination	34	23	7.99E-05	Shared
**Controls**	GO:0044784	metaphase/anaphase transition of cell cycle	22	31	8.31E-05	Univocal
**Controls**	GO:0048646	anatomical structure formation involved in morphogenesis	144	13	9.14E-05	Univocal
**Controls**	GO:0051304	chromosome separation	28	26	9.54E-05	Shared
**Controls**	GO:0000077	DNA damage checkpoint	40	21	1.00E-04	Shared
**Controls**	GO:0031055	chromatin remodeling at centromere	16	40	1.05E-04	Shared
**Controls**	GO:0000086	G2/M transition of mitotic cell cycle	56	18	1.07E-04	Shared
**Controls**	GO:0048513	animal organ development	350	11	1.13E-04	Univocal
**Controls**	GO:1905818	regulation of chromosome separation	23	29	1.15E-04	Univocal
**Controls**	GO:0009888	tissue development	219	12	1.16E-04	Univocal
**Controls**	GO:0009790	embryo development	139	13	1.18E-04	Univocal
**Controls**	GO:0048608	reproductive structure development	72	16	1.37E-04	Univocal
**Controls**	GO:0032201	telomere maintenance via semi-conservative replication	15	42	1.43E-04	Shared
**Controls**	GO:0031099	regeneration	43	20	1.48E-04	Univocal
**Controls**	GO:1901989	positive regulation of cell cycle phase transition	29	25	1.60E-04	Shared
**Controls**	GO:0061982	meiosis I cell cycle process	27	26	1.98E-04	Shared
**Controls**	GO:0072331	signal transduction by p53 class mediator	58	17	1.99E-04	Shared
**Controls**	GO:2000816	negative regulation of mitotic sister chromatid separation	18	35	2.12E-04	Univocal
**Controls**	GO:0042476	odontogenesis	30	24	2.51E-04	Univocal
**Controls**	GO:0060429	epithelium development	146	13	2.82E-04	Univocal
**Controls**	GO:0045931	positive regulation of mitotic cell cycle	39	20	2.97E-04	Shared
**Controls**	GO:0033047	regulation of mitotic sister chromatid segregation	24	27	3.07E-04	Shared
**Controls**	GO:0042475	odontogenesis of dentin-containing tooth	23	28	3.17E-04	Univocal
**Controls**	GO:0022616	DNA strand elongation	13	45	3.81E-04	Shared
**Controls**	GO:0071824	protein-DNA complex subunit organization	50	18	4.28E-04	Shared
**Controls**	GO:1902749	regulation of cell cycle G2/M phase transition	48	18	6.06E-04	Shared
**Controls**	GO:0034508	centromere complex assembly	17	34	6.45E-04	Shared
**Controls**	GO:0032392	DNA geometric change	28	24	7.08E-04	Shared
**Controls**	GO:0000083	regulation of transcription involved in G1/S transition of mitotic cell cycle	14	40	7.18E-04	Shared
**Controls**	GO:2001251	negative regulation of chromosome organization	36	21	7.34E-04	Shared

**Controls**	GO:0030330	DNA damage response, signal transduction by p53 class mediator	31	22	7.44E-04	Shared
**Controls**	GO:0006336	DNA replication-independent nucleosome assembly	17	33	8.91E-04	Shared
**Controls**	GO:0009611	response to wounding	93	14	0.00111	Univocal
**Controls**	GO:0000079	regulation of cyclin-dependent protein serine/threonine kinase activity	29	23	0.00124	Univocal
**Controls**	GO:0021915	neural tube development	38	20	0.00129	Univocal
**Controls**	GO:0034728	nucleosome organization	32	21	0.00139	Shared
**Controls**	GO:0032508	DNA duplex unwinding	25	24	0.00190	Shared
**Controls**	GO:0031297	replication fork processing	15	35	0.00219	Shared
**Controls**	GO:0071897	DNA biosynthetic process	44	18	0.00270	Shared
**Controls**	GO:0051225	spindle assembly	27	23	0.00292	Shared
**Controls**	GO:0007127	meiosis I	24	24	0.00387	Shared
**Controls**	GO:0048598	embryonic morphogenesis	87	14	0.00389	Univocal
**Controls**	GO:0042981	regulation of apoptotic process	185	12	0.00410	Univocal
**Controls**	GO:0031571	mitotic G1 DNA damage checkpoint	21	26	0.00447	Shared
**Controls**	GO:1901992	positive regulation of mitotic cell cycle phase transition	24	24	0.00466	Shared
**Controls**	GO:0048732	gland development	72	15	0.00472	Univocal
**Controls**	GO:0006297	nucleotide-excision repair, DNA gap filling	12	40	0.00498	Shared
**Controls**	GO:0010212	response to ionizing radiation	33	20	0.00650	Univocal
**Controls**	GO:0048514	blood vessel morphogenesis	86	14	0.00669	Univocal
**Controls**	GO:1902751	positive regulation of cell cycle G2/M phase transition	14	34	0.00670	Shared
**Controls**	GO:0004693	cyclin-dependent protein serine/threonine kinase activity	31	20	0.00754	Univocal
**Controls**	GO:0043486	histone exchange	16	30	0.00850	Shared
**Controls**	GO:1902806	regulation of cell cycle G1/S phase transition	39	18	0.00851	Univocal
**Controls**	GO:0051054	positive regulation of DNA metabolic process	47	17	0.00890	Shared
**Controls**	GO:0030261	chromosome condensation	14	33	0.00925	Shared
**Controls**	GO:0043009	chordate embryonic development	90	13	0.00933	Univocal
**Controls**	GO:2000134	negative regulation of G1/S transition of mitotic cell cycle	29	21	0.00986	Univocal
**Controls**	GO:0012501	programmed cell death	226	11	0.00988	Univocal
**RSTS**	GO:0000278	mitotic cell cycle	206	17	2.10E-45	Shared
**RSTS**	GO:1903047	mitotic cell cycle process	188	18	1.75E-44	Shared
**RSTS**	GO:0022402	cell cycle process	226	15	1.19E-38	Shared
**RSTS**	GO:0006261	DNA-dependent DNA replication	72	37	1.09E-35	Shared
**RSTS**	GO:0006260	DNA replication	95	27	7.11E-35	Shared
**RSTS**	GO:0006259	DNA metabolic process	179	16	6.68E-34	Shared
**RSTS**	GO:0044770	cell cycle phase transition	135	19	4.93E-31	Shared
**RSTS**	GO:0044772	mitotic cell cycle phase transition	128	19	1.22E-29	Shared
**RSTS**	GO:0000280	nuclear division	102	22	1.22E-28	Shared
**RSTS**	GO:0048285	organelle fission	104	20	2.00E-26	Shared
**RSTS**	GO:0140014	mitotic nuclear division	83	24	3.28E-26	Shared
**RSTS**	GO:0051276	chromosome organization	180	14	1.06E-25	Shared
**RSTS**	GO:0098813	nuclear chromosome segregation	71	25	1.99E-22	Shared
**RSTS**	GO:0044786	cell cycle DNA replication	38	45	1.96E-21	Shared
**RSTS**	GO:0000819	sister chromatid segregation	60	27	5.63E-21	Shared
**RSTS**	GO:0006281	DNA repair	106	17	5.04E-20	Shared
**RSTS**	GO:0006974	cellular response to DNA damage stimulus	138	14	1.35E-19	Shared
**RSTS**	GO:0000070	mitotic sister chromatid segregation	52	29	5.00E-19	Shared
**RSTS**	GO:0033260	nuclear DNA replication	33	46	8.01E-19	Shared
**RSTS**	GO:0007346	regulation of mitotic cell cycle	117	15	2.29E-17	Shared
**RSTS**	GO:0044843	cell cycle G1/S phase transition	68	21	3.05E-17	Shared
**RSTS**	GO:0010564	regulation of cell cycle process	128	14	8.78E-17	Shared
**RSTS**	GO:0071103	DNA conformation change	64	22	1.17E-16	Shared
**RSTS**	GO:0000082	G1/S transition of mitotic cell cycle	64	21	3.52E-16	Shared
**RSTS**	GO:0007088	regulation of mitotic nuclear division	53	23	5.59E-15	Shared
**RSTS**	GO:0000075	cell cycle checkpoint	59	21	6.15E-15	Shared
**RSTS**	GO:0006310	DNA recombination	62	20	1.38E-14	Shared
**RSTS**	GO:0007093	mitotic cell cycle checkpoint	50	24	1.39E-14	Shared
**RSTS**	GO:0051726	regulation of cell cycle	160	12	8.57E-14	Shared

**RSTS**	GO:1901987	regulation of cell cycle phase transition	87	16	9.09E-14	Shared
**RSTS**	GO:0006302	double-strand break repair	55	21	2.45E-13	Shared
**RSTS**	GO:0051321	meiotic cell cycle	52	22	4.22E-13	Shared
**RSTS**	GO:0006323	DNA packaging	44	24	8.63E-13	Shared
**RSTS**	GO:0006270	DNA replication initiation	22	50	9.03E-13	Shared
**RSTS**	GO:1901990	regulation of mitotic cell cycle phase transition	81	16	1.73E-12	Shared
**RSTS**	GO:0044839	cell cycle G2/M phase transition	62	18	1.79E-12	Shared
**RSTS**	GO:0051983	regulation of chromosome segregation	35	29	2.16E-12	Shared
**RSTS**	GO:0000724	double-strand break repair via homologous recombination	38	26	1.02E-11	Shared
**RSTS**	GO:0000226	microtubule cytoskeleton organization	88	14	1.24E-11	Shared
**RSTS**	GO:0090068	positive regulation of cell cycle process	62	18	1.29E-11	Shared
**RSTS**	GO:0032201	telomere maintenance via semi-conservative replication	19	53	2.46E-11	Shared
**RSTS**	GO:0051052	regulation of DNA metabolic process	75	15	2.85E-11	Shared
**RSTS**	GO:0045787	positive regulation of cell cycle	73	15	7.24E-11	Shared
**RSTS**	GO:0031570	DNA integrity checkpoint	44	21	1.28E-10	Shared
**RSTS**	GO:1903046	meiotic cell cycle process	41	23	1.39E-10	Shared
**RSTS**	GO:0090329	regulation of DNA-dependent DNA replication	25	35	2.11E-10	Shared
**RSTS**	GO:0006275	regulation of DNA replication	35	25	4.47E-10	Shared
**RSTS**	GO:0000723	telomere maintenance	42	21	7.27E-10	Univocal
**RSTS**	GO:0000086	G2/M transition of mitotic cell cycle	55	17	7.33E-10	Shared
**RSTS**	GO:0007051	spindle organization	41	21	9.20E-10	Shared
**RSTS**	GO:1901988	negative regulation of cell cycle phase transition	54	18	1.01E-09	Shared
**RSTS**	GO:0033045	regulation of sister chromatid segregation	29	28	1.23E-09	Shared
**RSTS**	GO:0006996	organelle organization	326	8	1.57E-09	Shared
**RSTS**	GO:0010948	negative regulation of cell cycle process	66	15	1.84E-09	Shared
**RSTS**	GO:0033554	cellular response to stress	210	9	2.41E-09	Shared
**RSTS**	GO:0051304	chromosome separation	29	27	5.96E-09	Shared
**RSTS**	GO:1902749	regulation of cell cycle G2/M phase transition	48	18	6.86E-09	Shared
**RSTS**	GO:0045930	negative regulation of mitotic cell cycle	61	16	7.62E-09	Shared
**RSTS**	GO:0045005	DNA-dependent DNA replication maintenance of fidelity	20	38	1.34E-08	Shared
**RSTS**	GO:0140013	meiotic nuclear division	36	22	1.50E-08	Shared
**RSTS**	GO:0022616	DNA strand elongation	15	52	2.21E-08	Shared
**RSTS**	GO:1902850	microtubule cytoskeleton organization involved in mitosis	34	22	3.35E-08	Shared
**RSTS**	GO:0006271	DNA strand elongation involved in DNA replication	12	67	4.07E-08	Shared
**RSTS**	GO:0033047	regulation of mitotic sister chromatid segregation	25	28	4.76E-08	Shared
**RSTS**	GO:0007091	metaphase/anaphase transition of mitotic cell cycle	22	32	5.92E-08	Univocal
**RSTS**	GO:0045839	negative regulation of mitotic nuclear division	22	32	5.92E-08	Univocal
**RSTS**	GO:0033046	negative regulation of sister chromatid segregation	21	33	6.34E-08	Shared
**RSTS**	GO:0051054	positive regulation of DNA metabolic process	48	17	6.70E-08	Shared
**RSTS**	GO:0071897	DNA biosynthetic process	44	18	8.44E-08	Shared
**RSTS**	GO:0061982	meiosis I cell cycle process	27	26	9.79E-08	Shared
**RSTS**	GO:0044774	mitotic DNA integrity checkpoint	31	23	1.09E-07	Shared
**RSTS**	GO:1901991	negative regulation of mitotic cell cycle phase transition	48	17	1.11E-07	Shared
**RSTS**	GO:0006333	chromatin assembly or disassembly	33	21	2.20E-07	Shared
**RSTS**	GO:0045132	meiotic chromosome segregation	24	28	2.31E-07	Univocal
**RSTS**	GO:0007052	mitotic spindle organization	29	23	2.84E-07	Shared
**RSTS**	GO:0032392	DNA geometric change	28	24	3.34E-07	Shared
**RSTS**	GO:0034508	centromere complex assembly	18	36	3.67E-07	Shared
**RSTS**	GO:0003887	DNA-directed DNA polymerase activity	15	44	3.94E-07	Univocal
**RSTS**	GO:0031055	chromatin remodeling at centromere	16	40	5.88E-07	Shared
**RSTS**	GO:0006334	nucleosome assembly	27	24	5.92E-07	Shared
**RSTS**	GO:0000077	DNA damage checkpoint	36	19	9.72E-07	Shared
**RSTS**	GO:0072331	signal transduction by p53 class mediator	51	15	1.12E-06	Shared
**RSTS**	GO:0045841	negative regulation of mitotic metaphase/anaphase transition	17	35	1.56E-06	Univocal

RSTS	GO:0045786	negative regulation of cell cycle	87	12	1.57E-06	Shared
RSTS	GO:0034061	DNA polymerase activity	25	24	2.31E-06	Univocal
RSTS	GO:0007098	centrosome cycle	32	20	3.56E-06	Shared
RSTS	GO:0006284	base-excision repair	18	32	4.21E-06	Univocal
RSTS	GO:0065004	protein-DNA complex assembly	40	17	4.24E-06	Shared
RSTS	GO:0006336	DNA replication-independent nucleosome assembly	17	33	4.56E-06	Shared
RSTS	GO:0007127	meiosis I	24	24	5.06E-06	Shared
RSTS	GO:0044773	mitotic DNA damage checkpoint	27	22	6.40E-06	Shared
RSTS	GO:0072422	signal transduction involved in DNA damage checkpoint	23	24	7.19E-06	Shared
RSTS	GO:0006297	nucleotide-excision repair, DNA gap filling	13	43	8.18E-06	Shared
RSTS	GO:0010389	regulation of G2/M transition of mitotic cell cycle	40	16	8.82E-06	Univocal
RSTS	GO:0032508	DNA duplex unwinding	24	23	9.50E-06	Shared
RSTS	GO:0006139	nucleobase-containing compound metabolic process	420	7	9.79E-06	Univocal
RSTS	GO:0045931	positive regulation of mitotic cell cycle	34	18	1.33E-05	Shared
RSTS	GO:0003678	DNA helicase activity	19	28	1.49E-05	Univocal
RSTS	GO:0006338	chromatin remodeling	32	18	1.86E-05	Univocal
RSTS	GO:0031297	replication fork processing	15	35	1.91E-05	Shared
RSTS	GO:0070192	chromosome organization involved in meiotic cell cycle	17	30	2.26E-05	Univocal
RSTS	GO:0034728	nucleosome organization	29	19	2.47E-05	Shared
RSTS	GO:1901989	positive regulation of cell cycle phase transition	25	21	3.00E-05	Shared
RSTS	GO:0036297	interstrand cross-link repair	18	28	3.25E-05	Univocal
RSTS	GO:0046483	heterocycle metabolic process	424	7	3.58E-05	Univocal
RSTS	GO:0006725	cellular aromatic compound metabolic process	426	7	3.68E-05	Univocal
RSTS	GO:0071824	protein-DNA complex subunit organization	42	15	4.19E-05	Shared
RSTS	GO:0042770	signal transduction in response to DNA damage	31	18	5.11E-05	Shared
RSTS	GO:0090304	nucleic acid metabolic process	377	7	5.46E-05	Univocal
RSTS	GO:0043486	histone exchange	16	30	6.53E-05	Shared
RSTS	GO:0000083	regulation of transcription involved in G1/S transition of mitotic cell cycle	13	37	7.53E-05	Shared
RSTS	GO:0031572	G2 DNA damage checkpoint	15	31	0.00010	Univocal
RSTS	GO:1901360	organic cyclic compound metabolic process	433	7	0.00012	Univocal
RSTS	GO:0000731	DNA synthesis involved in DNA repair	17	27	0.00012	Univocal
RSTS	GO:0042769	DNA damage response, detection of DNA damage	15	31	0.00014	Univocal
RSTS	GO:0004536	deoxyribonuclease activity	18	26	0.00015	Univocal
RSTS	GO:0051383	kinetochore organization	11	42	0.00017	Univocal
RSTS	GO:0051225	spindle assembly	24	20	0.00018	Shared
RSTS	GO:0090305	nucleic acid phosphodiester bond hydrolysis	45	14	0.00020	Univocal
RSTS	GO:2001251	negative regulation of chromosome organization	30	17	0.00023	Shared
RSTS	GO:0034641	cellular nitrogen compound metabolic process	447	7	0.00042	Univocal
RSTS	GO:0000076	DNA replication checkpoint	9	50	0.00042	Univocal
RSTS	GO:1900264	positive regulation of DNA-directed DNA polymerase activity	6	86	0.00045	Univocal
RSTS	GO:0033044	regulation of chromosome organization	51	13	0.00052	Univocal
RSTS	GO:0034502	protein localization to chromosome	23	20	0.00054	Univocal
RSTS	GO:1901992	positive regulation of mitotic cell cycle phase transition	21	21	0.00065	Shared
RSTS	GO:0030261	chromosome condensation	13	31	0.00087	Shared
RSTS	GO:0006268	DNA unwinding involved in DNA replication	8	53	0.00106	Univocal
RSTS	GO:1902969	mitotic DNA replication	8	53	0.00106	Univocal
RSTS	GO:0046112	nucleobase biosynthetic process	10	40	0.00120	Univocal
RSTS	GO:0051303	establishment of chromosome localization	17	23	0.00153	Univocal
RSTS	GO:0031571	mitotic G1 DNA damage checkpoint	18	22	0.00154	Shared
RSTS	GO:0043044	ATP-dependent chromatin remodeling	18	22	0.00154	Univocal
RSTS	GO:0045740	positive regulation of DNA replication	14	27	0.00164	Univocal
RSTS	GO:0019439	aromatic compound catabolic process	79	10	0.00174	Univocal
RSTS	GO:0008608	attachment of spindle microtubules to kinetochore	12	32	0.00186	Univocal

**RSTS**	GO:0009112	nucleobase metabolic process	13	29	0.00210	Univocal
**RSTS**	GO:0034655	nucleobase-containing compound catabolic process	74	10	0.00236	Univocal
**RSTS**	GO:0007062	sister chromatid cohesion	17	22	0.00277	Univocal
**RSTS**	GO:0071459	protein localization to chromosome, centromeric region	11	33	0.00282	Univocal
**RSTS**	GO:0044270	cellular nitrogen compound catabolic process	77	10	0.00348	Univocal
**RSTS**	GO:0030330	DNA damage response, signal transduction by p53 class mediator	24	17	0.00350	Shared
**RSTS**	GO:0001556	oocyte maturation	10	36	0.00406	Univocal
**RSTS**	GO:1902751	positive regulation of cell cycle G2/M phase transition	12	29	0.00457	Shared
**RSTS**	GO:0007080	mitotic metaphase plate congression	13	27	0.00466	Univocal
**RSTS**	GO:0045143	homologous chromosome segregation	13	27	0.00466	Univocal
**RSTS**	GO:0007010	cytoskeleton organization	124	9	0.00555	Shared
**RSTS**	GO:0044818	mitotic G2/M transition checkpoint	12	29	0.00603	Univocal
**RSTS**	GO:0046700	heterocycle catabolic process	76	10	0.00656	Univocal
**RSTS**	GO:0006977	DNA damage response, signal transduction by p53 class mediator resulting in cell cycle arrest	16	22	0.00732	Univocal
**RSTS**	GO:0006301	postreplication repair	15	23	0.00733	Univocal
**RSTS**	GO:0042276	error-prone translesion synthesis	9	38	0.00808	Univocal
**RSTS**	GO:1901796	regulation of signal transduction by p53 class mediator	32	14	0.00852	Univocal
**RSTS**	GO:1901361	organic cyclic compound catabolic process	79	10	0.00865	Univocal
**RSTS**	GO:0009113	purine nucleobase biosynthetic process	7	50	0.00939	Univocal
