# Supplementary material for: Transcriptome Analysis of iPSC-Derived Neurons from Rubinstein-Taybi Patients Reveals Deficits in Neuronal Differentiation
Source: Mol Neurobiol. 2020 Jun 20;57(9):3685–701. doi: 10.1007/s12035-020-01983-6 (PMC7399686; doi:10.1007/s12035-020-01983-6)
Supplement: Supplementary file 6 — Additional File 6 (Additional_File_6.pdf). REVIGO treemaps of 224 up- biological processes shared by RSTS and controls. REVIGO treemaps summarizing Gene Ontology (GO) (a) biological processes and (b) molecular functions URGs-enriched categories (224) shared by RSTS and controls (see Fig. 2b- left side). For each panel, not all URGs-enriched terms are reported due to space constraints. (PDF 1868 kb) [file 12035_2020_1983_MOESM6_ESM.pdf]

Additional file 6

REVIGO treemaps of 224 up- biological processes shared by RSTS and controls.

Shared GO terms from URGs

a) Biological processes (197)

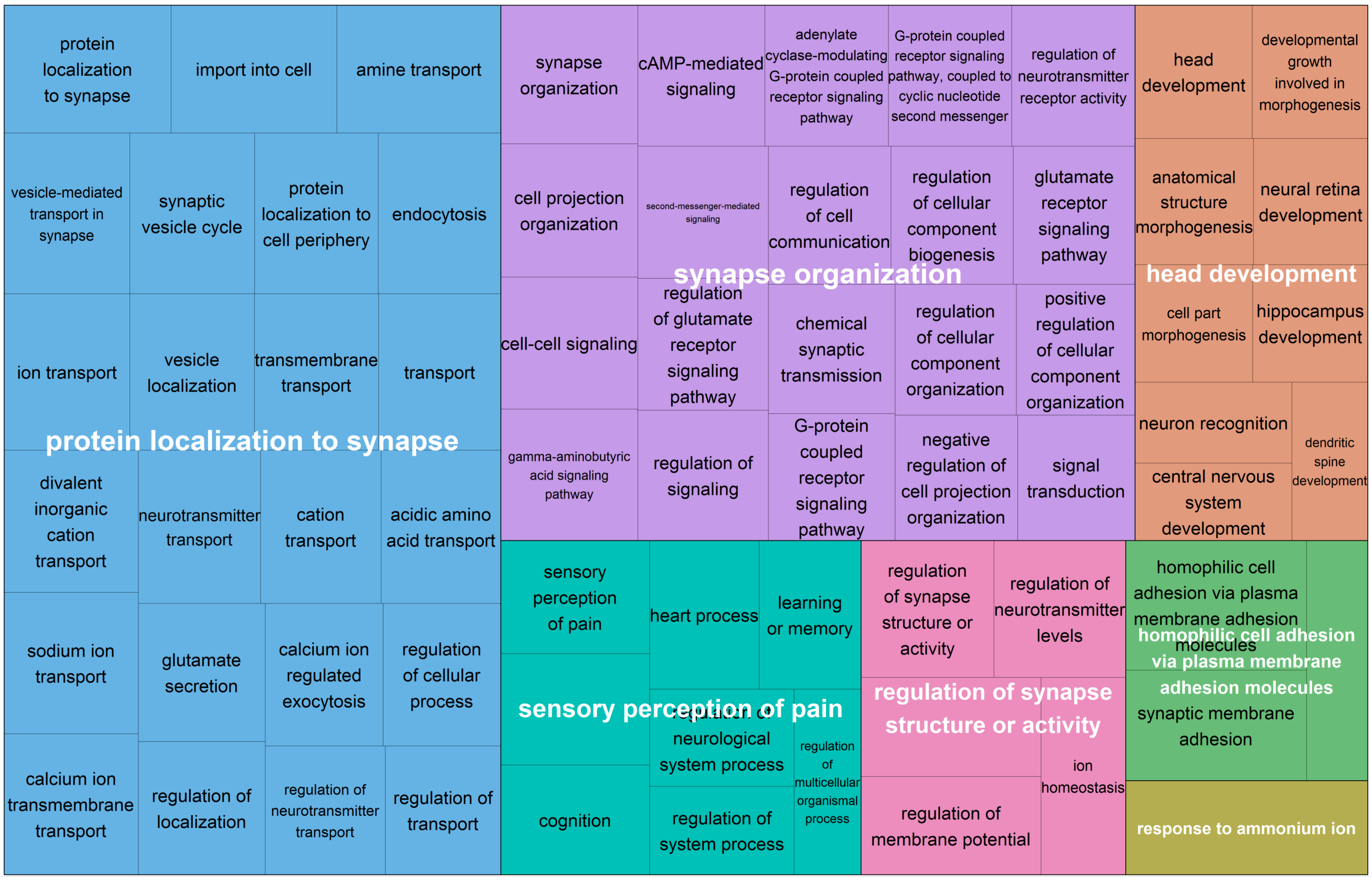

b) Molecular function (22)

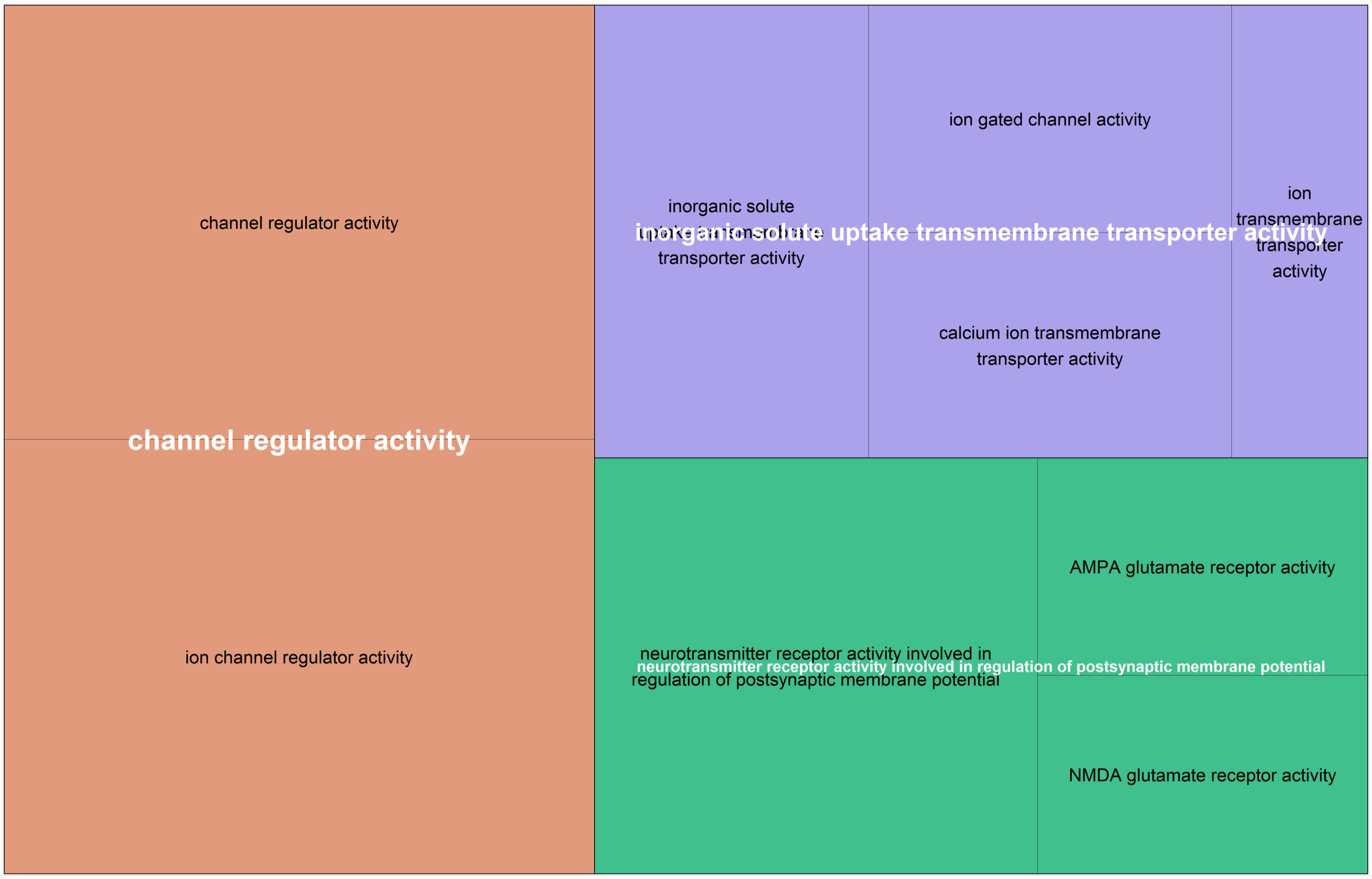

5 GO terms were not found in the current version of the GeneOntology
