# Supplementary material for: Transcriptome Analysis of iPSC-Derived Neurons from Rubinstein-Taybi Patients Reveals Deficits in Neuronal Differentiation
Source: Mol Neurobiol. 2020 Jun 20;57(9):3685–701. doi: 10.1007/s12035-020-01983-6 (PMC7399686; doi:10.1007/s12035-020-01983-6)
Supplement: Supplementary file 7 — Additional File 7 (Additional_File_7.pdf). REVIGO treemap of 102 down- biological processes shared by RSTS and controls. REVIGO treemap summarizing Gene Ontology (GO) biological processes DRGs-enriched categories (102) shared by RSTS and controls (see Fig. 2b- right side). For each panel, not all DRGs-enriched terms are reported due to space constraints. (PDF 843 kb) [file 12035_2020_1983_MOESM7_ESM.pdf]

Additional file 7

REVIGO treemap of 102 down- biological processes shared by RSTS and controls.

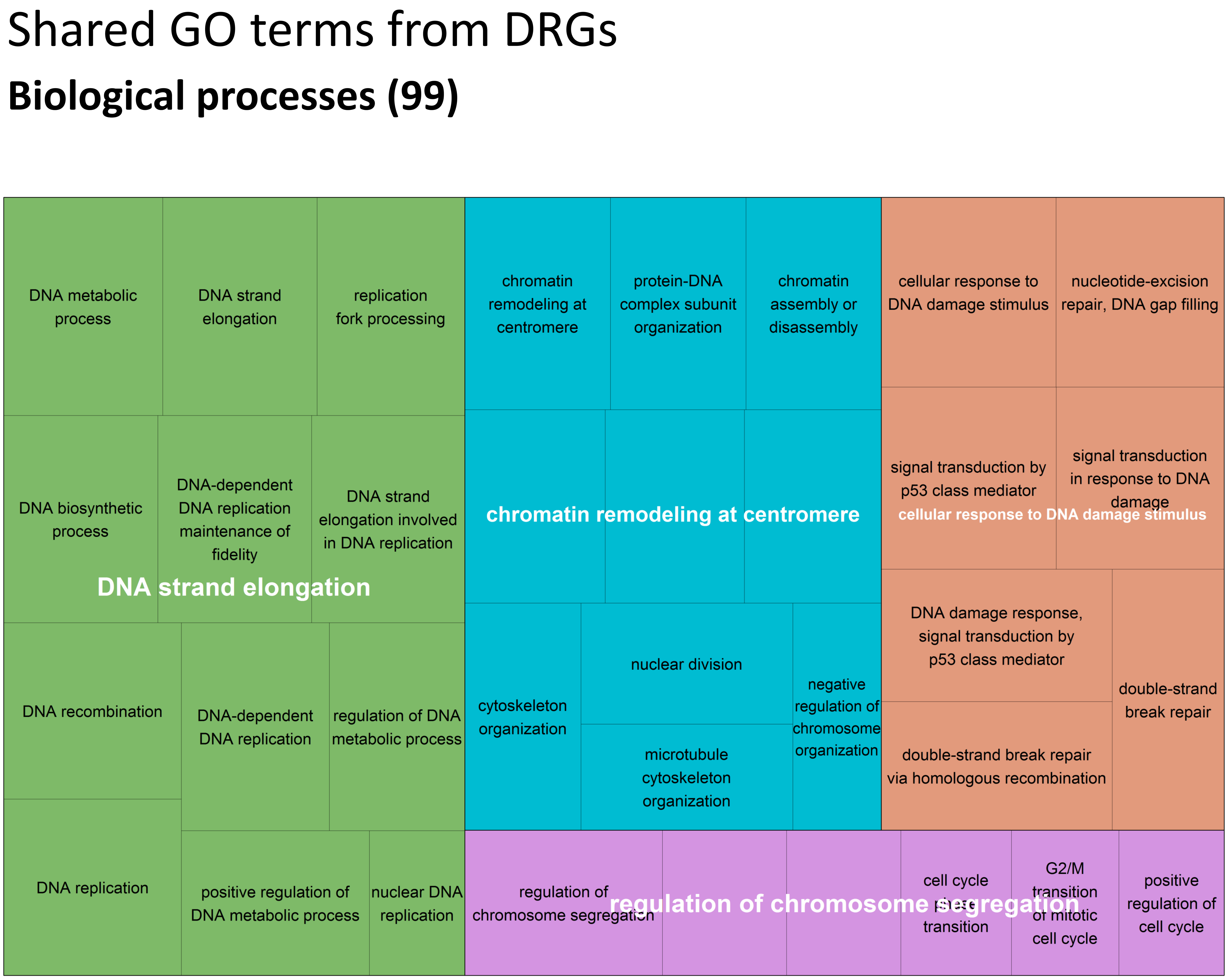

3 GO terms were not found in the current version of the GeneOntology
