# Supplementary material for: Transcriptome Analysis of iPSC-Derived Neurons from Rubinstein-Taybi Patients Reveals Deficits in Neuronal Differentiation
Source: Mol Neurobiol. 2020 Jun 20;57(9):3685–701. doi: 10.1007/s12035-020-01983-6 (PMC7399686; doi:10.1007/s12035-020-01983-6)
Supplement: Supplementary file 8 — Additional File 8 (Additional_File_8.pdf). Gene Ontology (GO) enrichment of univocal URGs of control and RSTS groups. List of significant (padj<0.05) biological processes enriched in controls (n = 74) and RSTS (n = 43) URGs. Groups, GO terms identification codes and names are reported in the first three columns, respectively. Additional columns show information related to GO terms clustering/enrichment. (PDF 239 kb) [file 12035_2020_1983_MOESM8_ESM.pdf]

Additional File 8

Gene Ontology (GO) enrichment of univocal URGs of control and RSTS groups

GROUP	GOID	GOTerm	GOGroups	Nr. Genes	% Associated Genes	Term PValue (Bonferroni step down)
**Controls**	GO:0099536	synaptic signaling	Group07	69	9	1.50E-09
**Controls**	GO:0007268	chemical synaptic transmission	Group07	65	9	3.50E-08
**Controls**	GO:0042391	regulation of membrane potential	Group05, Group06, Group07, Group09	44	10	5.60E-07
**Controls**	GO:0051960	regulation of nervous system development	Group08	75	7	4.90E-06
**Controls**	GO:0015267	channel activity	Group06, Group09	52	8	8.10E-06
**Controls**	GO:0048699	generation of neurons	Group08	104	6	8.30E-06
**Controls**	GO:0050804	modulation of chemical synaptic transmission	Group05, Group07	45	9	8.50E-06
**Controls**	GO:0022839	ion gated channel activity	Group06, Group09	41	10	8.80E-06
**Controls**	GO:0030182	neuron differentiation	Group08	97	6	8.80E-06
**Controls**	GO:0045664	regulation of neuron differentiation	Group08	60	8	1.00E-05
**Controls**	GO:0050767	regulation of neurogenesis	Group08	68	7	1.70E-05
**Controls**	GO:0007399	nervous system development	Group08	141	5	2.60E-05
**Controls**	GO:0005216	ion channel activity	Group06, Group09	48	8	5.00E-05
**Controls**	GO:0022008	neurogenesis	Group08	106	6	5.60E-05
**Controls**	GO:0099175	regulation of postsynapse organization	Group04, Group08	19	15	7.60E-05
**Controls**	GO:0050807	regulation of synapse organization	Group04, Group08	29	11	8.80E-05
**Controls**	GO:0016358	dendrite development	Group04, Group08	30	11	9.80E-05
**Controls**	GO:0006812	cation transport	Group09	76	7	9.90E-05
**Controls**	GO:0050877	nervous system process	Group06, Group07	69	7	1.80E-04
**Controls**	GO:0098655	cation transmembrane transport	Group09	65	7	1.90E-04
**Controls**	GO:0022890	inorganic cation transmembrane transporter activity	Group09	56	7	2.00E-04
**Controls**	GO:0005261	cation channel activity	Group06, Group09	41	8	2.30E-04
**Controls**	GO:0007267	cell-cell signaling	Group07	99	6	2.40E-04
**Controls**	GO:0050808	synapse organization	Group04	41	8	3.00E-04
**Controls**	GO:0051668	localization within membrane	Group03, Group09	22	12	3.50E-04
**Controls**	GO:0099003	vesicle-mediated transport in synapse	Group03, Group07	25	11	7.70E-04
**Controls**	GO:1904062	regulation of cation transmembrane transport	Group09	33	9	7.80E-04
**Controls**	GO:0048666	neuron development	Group08	78	6	8.20E-04
**Controls**	GO:0015318	inorganic molecular entity transmembrane transporter activity	Group06, Group09	65	7	9.90E-04
**Controls**	GO:0098660	inorganic ion transmembrane transport	Group09	60	7	1.10E-03
**Controls**	GO:0043269	regulation of ion transport	Group06, Group09	50	7	1.10E-03
**Controls**	GO:0060078	regulation of postsynaptic membrane potential	Group05, Group06, Group07, Group09	19	13	1.30E-03
**Controls**	GO:0034220	ion transmembrane transport	Group09	76	6	1.40E-03
**Controls**	GO:0033555	multicellular organismal response to stress	Group02	14	16	1.60E-03
**Controls**	GO:0099173	postsynapse organization	Group04, Group08, Group09	23	11	1.90E-03
**Controls**	GO:0030001	metal ion transport	Group09	60	7	1.90E-03
**Controls**	GO:0034765	regulation of ion transmembrane transport	Group06, Group09	39	8	2.20E-03
**Controls**	GO:0006836	neurotransmitter transport	Group07	28	9	2.30E-03
**Controls**	GO:0048813	dendrite morphogenesis	Group04, Group08	21	11	2.50E-03
**Controls**	GO:0032594	protein transport within lipid bilayer	Group03, Group09	12	18	2.60E-03
**Controls**	GO:0048812	neuron projection morphogenesis	Group08	53	7	2.60E-03
**Controls**	GO:0005244	voltage-gated ion channel activity	Group09	26	10	2.70E-03
**Controls**	GO:0099565	chemical synaptic transmission, postsynaptic	Group05, Group06, Group07, Group09	19	12	2.90E-03
**Controls**	GO:0050773	regulation of dendrite development	Group04, Group08	20	11	3.20E-03
**Controls**	GO:0007186	G protein-coupled receptor signaling pathway	Group01	53	7	3.40E-03
**Controls**	GO:0006811	ion transport	Group09	91	6	4.20E-03
**Controls**	GO:0034762	regulation of transmembrane transport	Group09	43	7	4.20E-03
**Controls**	GO:0051049	regulation of transport	Group09	101	5	4.70E-03
**Controls**	GO:0015075	ion transmembrane transporter activity	Group09	66	6	5.30E-03
**Controls**	GO:0007215	glutamate receptor signaling pathway	Group05, Group09	16	13	5.50E-03
**Controls**	GO:0031175	neuron projection development	Group08	69	6	5.80E-03
**Controls**	GO:2000311	regulation of AMPA receptor activity	Group09	8	27	5.80E-03
**Controls**	GO:0001505	regulation of neurotransmitter levels	Group07	32	8	5.90E-03

**Controls**	GO:2001257	regulation of cation channel activity	Group09	21	11	6.60E-03
**Controls**	GO:0099072	regulation of postsynaptic membrane neurotransmitter receptor levels	Group03, Group09	13	15	7.30E-03
**Controls**	GO:0022857	transmembrane transporter activity	Group09	73	6	7.90E-03
**Controls**	GO:0048814	regulation of dendrite morphogenesis	Group04, Group08	15	13	1.00E-02
**Controls**	GO:0098662	inorganic cation transmembrane transport	Group09	53	7	1.00E-02
**Controls**	GO:0055085	transmembrane transport	Group09	89	6	1.10E-02
**Controls**	GO:1900449	regulation of glutamate receptor signaling pathway	Group04, Group05, Group09	13	15	1.10E-02
**Controls**	GO:0004970	ionotropic glutamate receptor activity	Group05, Group09	13	15	1.20E-02
**Controls**	GO:0007218	neuropeptide signaling pathway	Group00	13	15	1.20E-02
**Controls**	GO:0010975	regulation of neuron projection development	Group08	42	7	1.30E-02
**Controls**	GO:0015276	ligand-gated ion channel activity	Group05, Group09	22	10	1.80E-02
**Controls**	GO:0099637	neurotransmitter receptor transport	Group03, Group07, Group09	10	18	1.90E-02
**Controls**	GO:0120035	regulation of plasma membrane bounded cell projection organization	Group08	51	6	2.00E-02
**Controls**	GO:0060079	excitatory postsynaptic potential	Group05, Group06, Group09	14	13	2.10E-02
**Controls**	GO:0048265	response to pain	Group02	8	22	2.40E-02
**Controls**	GO:0022604	regulation of cell morphogenesis	Group08	39	7	2.50E-02
**Controls**	GO:0099601	regulation of neurotransmitter receptor activity	Group05, Group09	13	14	2.70E-02
**Controls**	GO:0032412	regulation of ion transmembrane transporter activity	Group09	25	9	3.20E-02
**Controls**	GO:0050890	cognition	Group07	28	8	3.20E-02
**Controls**	GO:0099590	neurotransmitter receptor internalization	Group03, Group09	7	24	4.30E-02
**Controls**	GO:0097479	synaptic vesicle localization	Group07	19	10	4.50E-02
**RSTS**	GO:0001655	urogenital system development	Group11	26	7	3.60E-05
**RSTS**	GO:0072006	nephron development	Group11	16	10	7.10E-05
**RSTS**	GO:0007423	sensory organ development	Group07	32	5	2.30E-04
**RSTS**	GO:0001656	metanephros development	Group11	12	12	5.60E-04
**RSTS**	GO:0072009	nephron epithelium development	Group11	13	10	7.50E-04
**RSTS**	GO:0048846	axon extension involved in axon guidance	Group08, Group10	8	19	7.70E-04
**RSTS**	GO:0072073	kidney epithelium development	Group11	15	9	1.10E-03
**RSTS**	GO:0061298	retina vasculature development in camera-type eye	Group07, Group09, Group10	6	29	1.20E-03
**RSTS**	GO:1902667	regulation of axon guidance	Group08, Group10	8	16	2.60E-03
**RSTS**	GO:0006935	chemotaxis	Group10	31	5	3.20E-03
**RSTS**	GO:0072080	nephron tubule development	Group11	11	11	3.20E-03
**RSTS**	GO:0072277	metanephric glomerular capillary formation	Group09, Group11	3	100	3.40E-03
**RSTS**	GO:0072028	nephron morphogenesis	Group11	10	12	3.40E-03
**RSTS**	GO:0072210	metanephric nephron development	Group09, Group11	8	16	3.50E-03
**RSTS**	GO:0061005	cell differentiation involved in kidney development	Group11	9	13	4.00E-03
**RSTS**	GO:0050920	regulation of chemotaxis	Group08, Group09, Group10	16	7	4.10E-03
**RSTS**	GO:0021637	trigeminal nerve structural organization	Group08	4	50	4.50E-03
**RSTS**	GO:0040012	regulation of locomotion	Group10	43	4	4.70E-03
**RSTS**	GO:0021559	trigeminal nerve development	Group08	5	31	5.00E-03
**RSTS**	GO:2000145	regulation of cell motility	Group10	40	4	8.80E-03
**RSTS**	GO:0060485	mesenchyme development	Group02	19	6	8.90E-03
**RSTS**	GO:0072170	metanephric tubule development	Group11	6	21	8.90E-03
**RSTS**	GO:0072164	mesonephric tubule development	Group11	11	10	9.10E-03
**RSTS**	GO:0051270	regulation of cellular component movement	Group10	42	4	1.10E-02
**RSTS**	GO:0001658	branching involved in ureteric bud morphogenesis	Group11	8	13	1.30E-02
**RSTS**	GO:0061440	kidney vasculature development	Group09, Group10	6	19	1.30E-02
**RSTS**	GO:0072202	cell differentiation involved in metanephros development	Group11	6	19	1.30E-02
**RSTS**	GO:0060041	retina development in camera-type eye	Group07, Group09	13	8	1.50E-02
**RSTS**	GO:0072078	nephron tubule morphogenesis	Group11	9	11	1.50E-02
**RSTS**	GO:0014896	muscle hypertrophy	Group01	10	10	1.50E-02
**RSTS**	GO:0072273	metanephric nephron morphogenesis	Group11	6	19	1.60E-02
**RSTS**	GO:0003183	mitral valve morphogenesis	Group03	4	36	1.90E-02
**RSTS**	GO:0009581	detection of external stimulus	Group06	11	9	2.20E-02

**RSTS**	GO:0061138	morphogenesis of a branching epithelium	Group11	14	7	2.70E-02
**RSTS**	GO:0072017	distal tubule development	Group11	4	33	2.80E-02
**RSTS**	GO:0072173	metanephric tubule morphogenesis	Group11	4	33	2.80E-02
**RSTS**	GO:0009582	detection of abiotic stimulus	Group06	11	8	3.00E-02
**RSTS**	GO:0032650	regulation of interleukin-1 alpha production	Group00	3	60	3.10E-02
**RSTS**	GO:0021612	facial nerve structural organization	Group08	4	31	4.00E-02
**RSTS**	GO:0060976	coronary vasculature development	Group09, Group10	7	13	4.20E-02
**RSTS**	GO:0051272	positive regulation of cellular component movement	Group10	26	5	4.30E-02
**RSTS**	GO:0098742	cell-cell adhesion via plasma-membrane adhesion molecules	Group04	16	6	4.60E-02
**RSTS**	GO:1905952	regulation of lipid localization	Group05	11	8	4.70E-02
