# Supplementary material for: Transcriptome Analysis of iPSC-Derived Neurons from Rubinstein-Taybi Patients Reveals Deficits in Neuronal Differentiation
Source: Mol Neurobiol. 2020 Jun 20;57(9):3685–701. doi: 10.1007/s12035-020-01983-6 (PMC7399686; doi:10.1007/s12035-020-01983-6)
Supplement: Supplementary file 9 — Additional File 9 (Additional_File_9.pdf). Gene Ontology (GO) enrichment of univocal DRGs of controls and RSTS groups. List of significant (padj<0.05) biological processes enriched in controls (n = 80) and RSTS (n = 44) DRGs. GO terms identification codes and names are reported in the first three columns, respectively. Additional columns show information related to GO terms clustering/enrichment. (PDF 243 kb) [file 12035_2020_1983_MOESM9_ESM.pdf]

Additional File 9

Gene Ontology (GO) enrichment of univocal DRGs of control and RSTS groups

GROUP	GOID	GOTerm	GOGroups	Nr. Genes	% Associated Genes	Term PValue (Bonferroni step down)
**Controls**	GO:0048646	anatomical structure formation involved in morphogenesis	Group12	95	8	2.90E-08
**Controls**	GO:0035239	tube morphogenesis	Group12	79	9	1.10E-07
**Controls**	GO:0009653	anatomical structure morphogenesis	Group12	174	6	2.70E-07
**Controls**	GO:0009888	tissue development	Group09, Group12	133	7	5.40E-07
**Controls**	GO:0035295	tube development	Group12	87	8	3.70E-06
**Controls**	GO:0009887	animal organ morphogenesis	Group11, Group12	85	8	5.10E-06
**Controls**	GO:0048514	blood vessel morphogenesis	Group12	59	9	6.80E-06
**Controls**	GO:0072359	circulatory system development	Group12	89	8	7.00E-06
**Controls**	GO:0042127	regulation of cell population proliferation	Group12	117	7	9.00E-06
**Controls**	GO:0048513	animal organ development	Group12	197	6	9.80E-06
**Controls**	GO:0072358	cardiovascular system development	Group12	66	9	1.00E-05
**Controls**	GO:0042475	odontogenesis of dentin-containing tooth	Group10, Group12	18	21	1.20E-05
**Controls**	GO:0042476	odontogenesis	Group10	22	17	1.70E-05
**Controls**	GO:0060429	epithelium development	Group09, Group12	89	8	1.70E-05
**Controls**	GO:0048732	gland development	Group12	49	10	3.30E-05
**Controls**	GO:0031099	regeneration	Group06	29	13	5.50E-05
**Controls**	GO:0009611	response to wounding	Group07	59	9	5.90E-05
**Controls**	GO:0048870	cell motility	Group12	109	7	1.20E-04
**Controls**	GO:0043010	camera-type eye development	Group11	36	11	1.50E-04
**Controls**	GO:0007166	cell surface receptor signaling pathway	Group05	172	6	1.60E-04
**Controls**	GO:0001654	eye development	Group11	39	10	2.00E-04
**Controls**	GO:0001655	urogenital system development	Group12	39	10	2.00E-04
**Controls**	GO:0031100	animal organ regeneration	Group06	18	18	2.00E-04
**Controls**	GO:0007423	sensory organ development	Group11, Group12	52	9	2.40E-04
**Controls**	GO:0016477	cell migration	Group12	101	7	2.80E-04
**Controls**	GO:0007389	pattern specification process	Group12	44	9	3.40E-04
**Controls**	GO:0072001	renal system development	Group12	35	10	5.80E-04
**Controls**	GO:0042060	wound healing	Group07	48	9	6.50E-04
**Controls**	GO:0008284	positive regulation of cell population proliferation	Group12	71	8	8.00E-04
**Controls**	GO:0007010	cytoskeleton organization	Group00	96	7	8.90E-04
**Controls**	GO:0048729	tissue morphogenesis	Group12	57	8	1.20E-03
**Controls**	GO:0003007	heart morphogenesis	Group12	30	11	2.30E-03
**Controls**	GO:0050678	regulation of epithelial cell proliferation	Group12	36	10	2.80E-03
**Controls**	GO:0072073	kidney epithelium development	Group12	22	13	3.10E-03
**Controls**	GO:0042981	regulation of apoptotic process	Group08	104	6	3.90E-03
**Controls**	GO:0001822	kidney development	Group12	32	10	4.40E-03
**Controls**	GO:0002088	lens development in camera-type eye	Group11	14	18	4.50E-03
**Controls**	GO:0060562	epithelial tube morphogenesis	Group12	34	10	5.40E-03
**Controls**	GO:0022612	gland morphogenesis	Group12	19	14	5.50E-03
**Controls**	GO:0048754	branching morphogenesis of an epithelial tube	Group10, Group12	21	13	7.50E-03
**Controls**	GO:0090596	sensory organ morphogenesis	Group11, Group12	28	10	8.20E-03
**Controls**	GO:0061326	renal tubule development	Group12	16	15	8.40E-03
**Controls**	GO:0001525	angiogenesis	Group12	45	8	8.70E-03
**Controls**	GO:0003006	developmental process involved in reproduction	Group02	53	8	8.70E-03
**Controls**	GO:0051270	regulation of cellular component movement	Group12	73	7	9.70E-03
**Controls**	GO:0030154	cell differentiation	Group12	207	5	9.90E-03
**Controls**	GO:0072006	nephron development	Group12	20	13	1.10E-02
**Controls**	GO:0060425	lung morphogenesis	Group12	11	21	1.20E-02
**Controls**	GO:0060541	respiratory system development	Group12	24	11	1.20E-02
**Controls**	GO:0030324	lung development	Group12	22	12	1.30E-02
**Controls**	GO:0030850	prostate gland development	Group12	11	20	1.40E-02
**Controls**	GO:0001763	morphogenesis of a branching structure	Group10, Group12	24	11	1.50E-02
**Controls**	GO:0007507	heart development	Group12	50	8	1.70E-02
**Controls**	GO:0010941	regulation of cell death	Group08	108	6	2.00E-02
**Controls**	GO:0072009	nephron epithelium development	Group12	17	14	2.10E-02

**Controls**	GO:0060993	kidney morphogenesis	Group12	15	15	2.20E-02
**Controls**	GO:0009799	specification of symmetry	Group12	17	13	2.30E-02
**Controls**	GO:0030855	epithelial cell differentiation	Group09, Group12	49	8	2.30E-02
**Controls**	GO:0060572	morphogenesis of an epithelial bud	Group12	6	40	2.40E-02
**Controls**	GO:0097186	amelogenesis	Group10	7	32	2.70E-02
**Controls**	GO:0021915	neural tube development	Group12	22	11	2.80E-02
**Controls**	GO:0045667	regulation of osteoblast differentiation	Group04	17	13	2.80E-02
**Controls**	GO:0021532	neural tube patterning	Group12	10	21	2.90E-02
**Controls**	GO:0002009	morphogenesis of an epithelium	Group12	45	8	3.00E-02
**Controls**	GO:2000145	regulation of cell motility	Group12	67	7	3.00E-02
**Controls**	GO:0007498	mesoderm development	Group12	17	13	3.10E-02
**Controls**	GO:0040012	regulation of locomotion	Group12	71	7	3.20E-02
**Controls**	GO:0048731	system development	Group12	235	5	3.20E-02
**Controls**	GO:0007275	multicellular organism development	Group12	255	5	3.30E-02
**Controls**	GO:0051093	negative regulation of developmental process	Group12	70	7	3.40E-02
**Controls**	GO:0008544	epidermis development	Group09	30	9	3.40E-02
**Controls**	GO:0007165	signal transduction	Group05	262	5	3.50E-02
**Controls**	GO:0060675	ureteric bud morphogenesis	Group12	12	17	3.60E-02
**Controls**	GO:0048518	positive regulation of biological process	Group01	282	5	3.60E-02
**Controls**	GO:0003002	regionalization	Group12	33	9	3.60E-02
**Controls**	GO:0031016	pancreas development	Group12	13	16	3.90E-02
**Controls**	GO:0072088	nephron epithelium morphogenesis	Group12	13	16	3.90E-02
**Controls**	GO:0061448	connective tissue development	Group12	28	10	4.20E-02
**Controls**	GO:0043062	extracellular structure organization	Group03	36	9	4.30E-02
**Controls**	GO:0060485	mesenchyme development	Group12	29	9	4.30E-02
**RSTS**	GO:0006396	RNA processing	Group7	47	5	1.00E-08
**RSTS**	GO:0022613	ribonucleoprotein complex biogenesis	Group4,Group7	32	6	1.90E-08
**RSTS**	GO:0042254	ribosome biogenesis	Group3,Group7	23	7	3.10E-07
**RSTS**	GO:0016071	mRNA metabolic process	Group7	42	4	3.80E-07
**RSTS**	GO:0006259	DNA metabolic process	Group6	46	4	4.20E-07
**RSTS**	GO:0009168	purine ribonucleoside monophosphate biosynthetic process	Group5	7	32	4.40E-06
**RSTS**	GO:0006364	rRNA processing	Group7	18	8	5.10E-06
**RSTS**	GO:0034655	nucleobase-containing compound catabolic process	Group7	33	5	5.10E-06
**RSTS**	GO:0006401	RNA catabolic process	Group7	24	5	3.00E-05
**RSTS**	GO:0006614	SRP-dependent cotranslational protein targeting to membrane	Group7	12	11	3.40E-05
**RSTS**	GO:0000398	mRNA splicing, via spliceosome	Group7	22	6	3.40E-05
**RSTS**	GO:0008380	RNA splicing	Group7	25	5	4.20E-05
**RSTS**	GO:0046112	nucleobase biosynthetic process	Group5	6	24	2.50E-04
**RSTS**	GO:0042273	ribosomal large subunit biogenesis	Group3,Group7	9	12	3.20E-04
**RSTS**	GO:0006913	nucleocytoplasmic transport	Group4,Group7	21	5	3.90E-04
**RSTS**	GO:0006402	mRNA catabolic process	Group7	21	5	4.20E-04
**RSTS**	GO:0051054	positive regulation of DNA metabolic process	Group6	17	6	4.40E-04
**RSTS**	GO:0000723	telomere maintenance	Group2,Group6	14	7	5.20E-04
**RSTS**	GO:0019083	viral transcription	Group7	14	7	8.10E-04
**RSTS**	GO:0044786	cell cycle DNA replication	Group6	9	11	8.70E-04
**RSTS**	GO:0006403	RNA localization	Group7	17	6	8.80E-04
**RSTS**	GO:0000956	nuclear-transcribed mRNA catabolic process	Group7	15	6	1.20E-03
**RSTS**	GO:0006260	DNA replication	Group6	18	5	1.70E-03
**RSTS**	GO:0006261	DNA-dependent DNA replication	Group6	13	7	1.70E-03
**RSTS**	GO:0006188	IMP biosynthetic process	Group5	4	36	1.90E-03
**RSTS**	GO:0000470	maturation of LSU-rRNA	Group3	5	23	2.10E-03
**RSTS**	GO:0006397	mRNA processing	Group7	24	4	2.60E-03
**RSTS**	GO:0006269	DNA replication, synthesis of RNA primer	Group6	3	60	3.70E-03
**RSTS**	GO:0034660	ncRNA metabolic process	Group7	24	4	3.70E-03
**RSTS**	GO:0071236	cellular response to antibiotic	Group1	12	7	4.40E-03
**RSTS**	GO:0071897	DNA biosynthetic process	Group2	14	6	4.80E-03
**RSTS**	GO:0051170	import into nucleus	Group4,Group7	12	6	6.80E-03
**RSTS**	GO:0008334	histone mRNA metabolic process	Group4	5	17	9.50E-03
**RSTS**	GO:0046129	purine ribonucleoside biosynthetic process	Group5	5	17	9.50E-03

**RSTS**	GO:0000387	spliceosomal snRNP assembly	Group4,Group7	6	12	1.30E-02
**RSTS**	GO:0051052	regulation of DNA metabolic process	Group6	20	4	1.30E-02
**RSTS**	GO:0072522	purine-containing compound biosynthetic process	Group5	16	5	1.40E-02
**RSTS**	GO:0050658	RNA transport	Group7	13	5	1.90E-02
**RSTS**	GO:0034404	nucleobase-containing small molecule biosynthetic process	Group5	13	5	1.90E-02
**RSTS**	GO:0032201	telomere maintenance via semi-conservative replication	Group2,Group6	5	14	2.20E-02
**RSTS**	GO:0009260	ribonucleotide biosynthetic process	Group5	15	5	2.60E-02
**RSTS**	GO:0022618	ribonucleoprotein complex assembly	Group4,Group7	14	5	2.90E-02
**RSTS**	GO:0097237	cellular response to toxic substance	Group1	13	5	4.20E-02
**RSTS**	GO:0051096	positive regulation of helicase activity	Group0	3	27	4.70E-02
