# Supplementary material for: Transcriptome Analysis of iPSC-Derived Neurons from Rubinstein-Taybi Patients Reveals Deficits in Neuronal Differentiation
Source: Mol Neurobiol. 2020 Jun 20;57(9):3685–701. doi: 10.1007/s12035-020-01983-6 (PMC7399686; doi:10.1007/s12035-020-01983-6)
Supplement: Supplementary file 10 — Additional File 10 (Additional_File10.pdf). Cluster analysis of univocal-URGs enriched GO terms (Controls and RSTS). Cluster lists from GO terms enriched in univocal controls and RSTS URGs. Relative group pvalues (corrected with Bonferroni step down), percentage of GO terms and gene names associated to clusters are provided in additional columns. (PDF 189 kb) [file 12035_2020_1983_MOESM10_ESM.pdf]

Additional file 10

Cluster analysis of univocal-URGs enriched GO terms (Controls and RSTS)

Group Cohort	Cluster	Leader GO term	padj	%	Group Genes
Controls	G09	ion gated channel activity	3.75E-06	34.783	*ABHD17C, ADCYAP1, ADORA2B, ANKH, ANO5, AQP11, ARC, ARG2, ARHGAP44, ARHGEF7, ARID4A, ARL6, ASIC4, ATAD1, ATP13A2, ATP6V1G2, BEST1, BNIP3L, BTBD9, CACNA1A, CACNB4, CACNG3, CADM1, CALY, CARTPT, CHMP2B, CHRNA3, CHRNA6, CLCN3, CLCN4, CNIH2, CORO1A, CP, CUX2, DAB2IP, DAGLA, DCN, EDNRB, EEF2K, EGLN1, EPS15, FAM155A, FAM155B, FGF13, FHL1, FKBP1B, FXYD6, FYN, GABRG2, GABRQ, GALR1, GIT1, GJD2, GLRB, GLRX, GNAQ, GNB5, GPR88, GRIN3A, GRIP1, GSK3B, HAP1, HMGCR, HTR2A, IFNAR1, IL1RAP, KCNA4, KCNB2, KCNC1, KCNH2, KCNH5, KCNK10, KCNK12, KCNK2, KCNV1, KLF7, LRFN1, LRFN4, MAP1B, MAPK10, MECP2, MEF2C, MFSD4A, MICU3, MMAA, MPP2, MPP3, MTTP, NETO1, NEUROD1, NIPA1, NKAIN1, NLGN3, NNAT, NOL3, NOS1, NPY, NRG1, OSBPL8, PARD6A, PEX5L, PIRT, PLA2G12A, PLCH2, PNOC, POU4F2, PPARGC1A, PPP1R11, PPP3CA, PREPL, PRKAB2, PRKAR1B, RAB15, RAB29, RAPGEF1, REEP2, RELN, REM2, RGS7BP, RHOT1, RHOV, RUBCN, RUFY2, SCN9A, SHISA6, SLC16A7, SLC23A2, SLC25A4, SLC38A1, SLC46A3, SLC6A11, SLC6A15, SLC6A5, SLC7A11, SLC8A1, SNX30, SORT1, SPHKAP, SPP1, SRC, SV2B, SYT12, SYT7, TAC1, TCF4, TET2, TFR2, TMEM163, TOMM40L, TRIB3, TRPC5, TSPAN13, TUB, WNT7A, YWHAG, ZFYVE9*
Controls	G08	regulation of nervous system development	2.42E-08	17.391	*ABHD17C, ADCYAP1, ADORA2B, ARC, ARHGAP44, ARHGEF7, ARL6, ATF5, AUTS2, BCHE, BEX2, BRINP3, CACNA1A, CAMKV, CAMSAP2, CARMIL2, CDHR1, CHAC1, CHMP2B, CHRNA3, CHST8, COCH, CORO1A, CPNE6, CUX2, DAB2IP, DAGLA, DCC, DIO3, DMBX1, DPF1, DPYSL5, DRGX, EDNRB, EEF2K, EFNA3, EGLN1, EN2, EPB41L3, EPHA6, EPHA8, ESPN, FGD4, FGF13, FKBP1B, FOXP2, FYN, GABRG2, GFRA2, GLRB, GRIN3A, GRIP1, GSDME, GSK3B, HAP1, HDAC5, HDGFL3, HMGCS1, ID4, IL1RAP, IL1RAPL2, INPP5J, IRX5, ITPK1, KCNC1, KIT, KLF7, LBX1, LHX4, LMO4, LRFN1, LRFN4, MACROD2, MAP1B, MBD5, MCF2, MECP2, MEF2C, MEIS1, MMD, MPP2, MPP3, MPPED2, MTTP, NAP1L2, NDNF, NEUROD1, NEUROD4, NHLH1, NLGN3, NNAT, NOL3, NOS1, NPY, NRG1, NTNG1, PALM2, PARP6, PAX5, PCSK2, POU3F2, POU4F1, POU4F2, PPARGC1A, PPP3CA, PRDM13, PTPRG, PTPRT, RAB18, RAB29, RAP1GAP, RAPGEF1, RCAN1, RELN, RET, RHOV, RPS6KA5, SARM1, SCN9A, SHISA6, SLC23A2, SLC25A4, SLC6A11, SLC7A11, SLC8A1, SOCS2, SPAST, SPHKAP, SPP1, SRC, SRD5A1, SSTR2, ST7, STMN3, SYNDIG1, TAB3, TAFA1, TBC1D24, TCF12, TCF4, TET2, THBS2, TIMP4, TMEM106B, TRAPPC6B, TRPC5, TTBK2, TUBA1A, TUBB2B, UCHL1, UNCX, WNT7A, YWHAG, ZEB1, ZFHX3, ZSWIM5*
Controls	G07	synaptic signaling	4.14E-10	12.174	*ADCYAP1, ADORA2B, ADRA1B, AKAP5, AMPH, AP3B2, ARC, ARG2, ARHGAP44, ARHGEF7, ARID4A, ARL6, ATAD1, BCHE, BEST1, BNIP3L, BTBD9, CACNA1A, CACNB4, CACNG3, CALY, CARTPT, CHMP2B, CHRNA3, CHRNA6, CNIH2, COCH, CPNE6, CUX2, CXXC4, DAAM1, DAB2IP, DAGLA, DCC, DCN, DOP1B, DRGX, EDNRB, EFNA3, EGLN1, EPS15, ESPN, FGF13, FHL1, FKBP1B, FOXP2, FYN, GABRG2, GABRQ, GALR1, GIT1, GJD2, GLRB, GLRX, GNAQ, GPR88, GRIN3A, GRIP1, GSDME, GSK3B, HAP1, HECA, HMGCR, HOXA5, HTR2A, IFNAR1, IL1RAP, IRX5, KCNA4, KCNC1, KCNH2, KCNH5, KCNK10, KCNK12, KCNK2, KIT, KLF7, LGR6, LIN7A, LIN7C, MAOA, MAPK10, MATK, MECP2, MEF2C, MPP2, MPP3, MTTP, NETO1, NEUROD1, NLGN3, NLK, NNAT, NOL3, NOS1, NPY, NRG1, NTNG1, OR2L13, PARD6A, PDYN, PLCL2, PNOC, POU4F1, POU4F2, PPEF1, PPP3CA, PREPL, PRKAR1B, RAPGEF1, RCAN1, REEP2, RELN, RET, RGS7BP, RHOT1, RNF180, SCN9A, SHISA6, SLC25A4, SLC38A1, SLC6A11, SLC6A15, SLC6A5, SLC7A11, SLC8A1, SPP1, SRC, SRD5A1, SV2B, SYN2, SYNDIG1, SYT12, SYT7, TAC1, TAFA2, TCF4, TET2, TFR2, TMEM170B, TMEM198, TRPC5, TUB, TUBB2B, UCHL1, UNC13C, VGF, WNT7A, YWHAG, ZBTB33*
Controls	G06	channel activity	1.04E-08	10.435	*ADCYAP1, ADORA2B, ANKH, ANO5, AQP11, ARC, ARID4A, ARL6, ASIC4, ATAD1, ATP13A2, ATP6V1G2, BCHE, BEST1, BNIP3L, BTBD9, CACNA1A, CACNB4, CACNG3, CARTPT, CHMP2B, CHRNA3, CHRNA6, CLCN3, CLCN4, CNIH2, COCH, CORO1A, CUX2, DCN, DOP1B, DRGX, EDNRB, ESPN, FAM155A, FAM155B, FGF13, FHL1, FKBP1B, FOXP2, FXYD6, FYN, GABRG2, GABRQ, GJD2, GLRB, GLRX, GNAQ, GNB5, GPR88, GRIN3A, GSDME, GSK3B, HAP1, HMGCR, HTR2A, IFNAR1, IRX5, KCNA4, KCNB2, KCNC1, KCNH2, KCNH5, KCNK10, KCNK12, KCNK2, KCNV1, KIT, MECP2, MEF2C, MMAA, MPP2, MPP3, MTTP, NETO1, NIPA1, NKAIN1, NLGN3, NOL3, NOS1, NPY, OR2L13, PIRT, PNOC, POU4F1, POU4F2, PPARGC1A, PPEF1, PPP3CA, PRKAR1B, RCAN1, REEP2, RELN, REM2, RET, RGS7BP, RHOV, SCN9A, SHISA6, SLC16A7, SLC23A2, SLC38A1, SLC6A11, SLC6A15, SLC6A5, SLC7A11, SLC8A1, SPHKAP, SRC, SYT12, SYT7, TAC1, TAFA2, TET2, TFR2, TOMM40L, TRPC5, TSPAN13, TUB, UCHL1, WNT7A*
Controls	G05	regulation of membrane potential	2.55E-10	8.696	*ADCYAP1, ADORA2B, ARC, ATAD1, BCHE, BNIP3L, CACNA1A, CACNB4, CACNG3, CHRNA3, CHRNA6, CNIH2, CUX2, DAGLA, DCC, DCN, EGLN1, FGF13, FHL1, FKBP1B, FYN, GABRG2, GABRQ, GIT1, GJD2, GLRB, GLRX, GNAQ, GPR88, GRIN3A, GSK3B, HAP1, HTR2A, KCNA4, KCNC1, KCNH2, KCNH5, KCNK10, KCNK12, KCNK2, KIT, MECP2, MEF2C, MPP2, MPP3, MTTP, NETO1, NLGN3, NOL3, NOS1, NTNG1, PLCL2, PPARGC1A, PPP3CA, PREPL, PRKAR1B, RELN, RGS7BP, RHOT1, SCN9A, SHISA6, SLC7A11, SLC8A1, SRC, SYT12, SYT7, TAC1, TRPC5, TUBB2B, UNC13C, WNT7A, YWHAG*

**Controls**	**G04**	**regulation of postsynapse organization**	1.25E-09	7.826	*ABHD17C, ARC, ARHGAP44, ARHGEF7, CACNA1A, CACNB4, CACNG3, CAMKV, CAMSAP2, CHMP2B, CHRNA3, CNIH2, CUX2, DAB2IP, DCC, EEF2K, EGLN1, FYN, GABRG2, GLRB, GRIN3A, GRIP1, GSK3B, IL1RAP, IL1RAPL2, KLF7, LRFN1, LRFN4, MAP1B, MCF2, MECP2, MEF2C, MPP2, MPP3, MTTP, NETO1, NLGN3, NOL3, NRG1, NTNG1, PARP6, PCDHGC4, POU4F1, PPARGC1A, PPP3CA, PTPRT, RAB29, RELN, SARM1, SHISA6, SLC7A11, SYNDIG1, TET2, THBS2, TMEM106B, TRPC5, TUBA1A, UNC13C, WNT7A*
**Controls**	**G03**	**localization within membrane**	4.22E-08	5.217	*ADORA2B, AMPH, AP3B2, ARC, ARHGAP44, ARL6, ATAD1, BTBD9, CACNA1A, CACNG3, CALY, CHRNA6, CNIH2, DAB2IP, EPS15, GIT1, GLRB, GRIN3A, GRIP1, GSK3B, HTR2A, LIN7A, MAPK10, MPP2, MPP3, NETO1, NLGN3, NOL3, PREPL, REEP2, RELN, SHISA6, SLC7A11, SYT12, SYT7, UNC13C, WNT7A, ZFYVE9*
**Controls**	**G02**	**multicellular organismal response to stress**	5.95E-06	1.739	*ADCYAP1, ASIC4, CACNA1A, EDNRB, MECP2, MEF2C, NOS1, PIRT, PPP3CA, RELN, RET, SCN9A, SLC6A5, TAC1*
**Controls**	**G01**	**G protein-coupled receptor signaling pathway**	8.50E-06	0.87	*ADCY9, ADCYAP1, ADORA2B, ADRA1B, AKAP5, ARHGEF7, ATRNL1, CACNA1A, CALY, CARTPT, DAGLA, DPYSL5, EDNRB, ELOVL4, FGD4, GABRG2, GALR1, GIT1, GLRB, GNAQ, GNB5, GNG3, GPR39, GPR88, GRK3, GSK3B, HTR2A, IFNAR1, IQGAP2, KCNK2, LGR6, MCF2, NOS1, NPY, NXPH3, OR2L13, PDYN, PLCL2, PNOC, PPEF1, PROKR1, RAPGEF1, RET, RGS7BP, SLC6A5, SORT1, SPHKAP, SRC, SSTR2, TAC1, TAC3, TMEM145, VAV3*
**Controls**	**G00**	**neuropeptide signaling pathway**	1.56E-05	0.87	*ADCYAP1, CARTPT, GALR1, GLRB, NPY, NXPH3, PDYN, PNOC, PROKR1, SORT1, SSTR2, TAC1, TAC3*
**RSTS**	**G11**	**urogenital system development**	2.19E-05	35.185	*AGT, CER1, CORO7, DCHS1, EGF, FAT4, IGFBP7, KLF15, LRP4, NFIA, NOTCH1, NRP1, OPTN, PAX8, PDGFA, PDGFRA, PDGFRB, PKD1, PLCE1, PODXL, POU3F3, PSAP, SALL1, SMAD9, SOX8, TNS2, WNT4*
**RSTS**	**G10**	**regulation of axon guidance**	2.15E-04	20.37	*ADRA2A, AGT, ALCAM, B4GAT1, BMPR2, CDH1, CHRM4, CORO7, CX3CL1, CXCL16, DDR2, EFNB3, EGF, ENC1, EPHA3, ERBB4, F3, HDAC4, HRH1, ISL1, LAMA2, LAMA4, LDLRAD4, LRP1, MCAM, MEGF8, MMP28, MSTN, NOTCH1, NRP1, OPTN, PDGFA, PDGFRA, PDGFRB, PDZD2, PKD1, PLEKHG5, PLPP3, PLXNA3, PODXL, PRR5L, PTPRM, PTPRR, RHOJ, SEMA3F, SEMA5B, SMAD9, SPTAN1, SUN2, VASH1, VEGFB, WNT4, ZSWIM8*
**RSTS**	**G09**	**retina vasculature development in camera-type eye**	2.58E-06	12.963	*ATP2B4, BMPR2, CORO7, F3, MEGF8, MMP28, MSTN, NOTCH1, NRP1, OPTN, PAX8, PDGFRA, PDGFRB, PKD1, PLXNA3, PRPH, PTPRM, RHOJ, RPE65, SALL1, SDK1, SEMA3F, SEMA5B, SMAD9, SOX8, VEGFB, WNT4, ZSWIM8*
**RSTS**	**G08**	**axon extension involved in axon guidance**	1.58E-05	11.111	*ALCAM, BMPR2, F3, ISL1, MEGF8, MMP28, MSTN, NOTCH1, NRP1, OPTN, PDGFRA, PDGFRB, PKD1, PLXNA3, SEMA3F, SEMA5B, VEGFB, ZSWIM8*
**RSTS**	**G07**	**sensory organ development**	6.45E-06	5.5556	*ADGRV1, ASCL1, ATP2B4, BMPR2, CDH1, CRYAB, DCHS1, FAT4, FSCN2, HMX3, JAG2, LRP4, NOTCH1, NRP1, OLFM3, OPTN, PAX8, PDGFRA, PDGFRB, PDZD7, PKD1, PRPH, PSAP, PTPRM, PTPRR, RHOJ, RPE65, SALL1, SDK1, SLITRK6, SOX8, TMIE*
**RSTS**	**G06**	**detection of external stimulus**	2.68E-04	3.7037	*ADGRV1, CDS1, GRK4, NTSR1, PDZD7, PKD1, PRDM12, PTPRR, RPE65, SEMA5B, TTN*
**RSTS**	**G05**	**regulation of lipid localization**	1.33E-04	1.8519	*ABCA2, ABCG1, AGT, CD36, EGF, FABP3, LIPG, LRP1, NTSR1, P2RX7, PPARA*
**RSTS**	**G04**	**cell-cell adhesion via plasma-membrane adhesion molecules**	2.60E-04	1.8519	*ALCAM, AMIGO2, CD36, CDH1, CX3CL1, DCHS1, FAT4, PCDH11Y, PCDHA12, PCDHB8, PCDHGA11, PKD1, PTPRM, SDK1, SLITRK2, TENM2*
**RSTS**	**G03**	**mitral valve morphogenesis**	2.68E-04	1.8519	*BMPR2, DCHS1, NOTCH1, SMAD9*
**RSTS**	**G02**	**mesenchyme development**	1.92E-04	1.8519	*BMPR2, CDKN2A, CER1, CORO7, DCHS1, EPHA3, ERBB4, ISL1, LDLRAD4, NOTCH1, NRP1, OPTN, PDGFRB, RANBP3L, SEMA3F, SEMA5B, SOX8, TAPT1, WNT4*
**RSTS**	**G01**	**muscle hypertrophy**	2.55E-04	1.8519	*AGT, ATP2B4, HDAC4, KLF15, MSTN, MYH7, NOTCH1, PDZD2, PPARA, TTN*
**RSTS**	**G00**	**regulation of interleukin-1 alpha production**	3.44E-04	1.8519	*CX3CL1, ISL1, P2RX7*
