# Supplementary material for: Transcriptome Analysis of iPSC-Derived Neurons from Rubinstein-Taybi Patients Reveals Deficits in Neuronal Differentiation
Source: Mol Neurobiol. 2020 Jun 20;57(9):3685–701. doi: 10.1007/s12035-020-01983-6 (PMC7399686; doi:10.1007/s12035-020-01983-6)
Supplement: Supplementary file 11 — Additional File 11 (Additional_File_11.pdf). Cluster analysis of univocal-DRGs enriched GO terms (Controls and RSTS). Cluster lists from GO terms enriched in univocal controls and RSTS DRGs. Relative group pvalues (corrected with Bonferroni step down), percentage of GO terms and gene names associated to clusters are provided in additional columns. (PDF 197 kb) [file 12035_2020_1983_MOESM11_ESM.pdf]

Additional file 11

Cluster analysis of univocal-DRGs enriched GO terms (Controls and RSTS)

Group Cohort	Cluster	Leader GO term	padj	%	Group Genes
**Controls**	**G12**	**anatomical structure formation involved in morphogenesis**	3.40E-08	69	*ACP6, ADAMTS1, ADAMTS12, ADAMTS18, ADAMTS9, AFP, AGPAT2, ALDH1A3, ANKRD1, ANO1, ANXA1, ANXA3, APOA1, APOE, APRT, ARHGEF19, ASB2, ASL, ATIC, AXIN2, B4GALT1, BAAT, BAK1, BAX, BCAR3, BMP4, BSG, BTG3, C14orf39, C5AR1, CASZ1, CCBE1, CCN2, CCND1, CCNJ, CCNL1, CCNO, CD4, CDK4, CDON, CEACAM21, CERS2, CES1, CFAP157, CFAP69, CFLAR, CFTR, CHMP4C, CIP2A, CISH, CLDN1, CLDN4, CLMN, CNN2, COL27A1, CORIN, CTDSPL, CTSZ, CXCL5, DLEU2, DMRT3, DNAAF1, DNAH11, DNAI2, DNMBP, DNPH1, DOCK8, DONSON, DSG2, DTYMK, E2F1, ECM1, EFHD1, EFNA5, ELF3, EMP2, ENO3, EPHA2, EPHA7, EPOP, EPPK1, ERBB2, ESM1, F11R, FAM110C, FAP, FES, FEZF1, FGF18, FGFR1OP, FLII, FLNA, FOSL1, FOXA1, FOXO1, FRMD6, FZD4, FZD6, GADD45B, GAL3ST1, GCNT1, GDF10, GDF15, GDNF, GLI3, GLRX5, GNPDA1, GORAB, GPD2, GPRC5A, GRHL3, HAPLN3, HDAC7, HES1, HHAT, HLA-DPB1, HOXB9, HSPA1B, HSPA2, HSPG2, ID1, ID3, IER3, IFITM1, IL1A, IL4R, INF2, INPP5D, ITGA6, ITGB1BP2, ITGB7, KANK1, KANK2, KCNN4, KCP, KIAA1522, KLK6, KREMEN2, KRT18, KRT8, LAMA5, LAMC3, LCTL, LDLRAP1, LGR5, LIF, LIM2, LMNB2, LMOD1, LMX1A, LPL, LRIG3, LRRC34, LZTS2, MAF, MAK, MAL, MAPK15, MCIDAS, MEOX1, MERTK, MFGE8, MGMT, MPZL2, MSX1, MSX2, MTR, MYCBPAP, MYH9, MYLK, MYO3A, NBEAL2, NEBL, NECTIN2, NEDD9, NEK8, NFKB2, NLRP2, NLRX1, NOG, NOP2, NOS3, NOX4, NPHP1, NPHP3, NR2F6, NRAP, NRTN, NXN, OFD1, OLIG3, OPTC, OSBPL1A, OTX1, P3H2, P3H3, PAK4, PAMR1, PAPSS2, PAX6, PAX7, PBX2, PDE6B, PERP, PGM5, PIH1D3, PITX2, PKHD1, PKM, PLAG1, PLEKHA4, PLS3, PMEL, PPL, PPP1CA, PPP1R13L, PRKD2, PRKDC, PRSS8, PTBP1, PTPN3, PTPN6, PTPRU, RAB32, RASSF3, RAX, RBM47, RBP1, RCC2, REST, RFX2, RHBDF1, RHBDF2, RHOH, RPS6KA1, RREB1, RSPO2, RSPO3, RTTN, S100A4, S100A6, S1PR2, SAMD11, SBNO2, SERPINA5, SERPINF1, SERTAD1, SFRP2, SH3D19, SHC1, SHH, SHROOM4, SIX6, SLC24A5, SLC25A37, SLC26A6, SLC2A1, SLC2A4, SLC4A11, SLC7A8, SLC8B1, SLC9A3R1, SLIT2, SMAD3, SMAD6, SMO, SMTN, SMYD2, SNAI1, SP1, SPAG6, SPHK1, STAT6, STX3, SULF1, SULT1A1, SYDE1, TAF4B, TBXAS1, TCF7L2, TCIRG1, TEAD3, TEK, TES, TFAP2C, TFPI2, TGFBI, TGFBR3, THBD, THNSL2, TIE1, TMEM88, TNFRSF10B, TNFRSF10D, TNNC1, TPBG, TPCN1, TRIM11, TRIM6, TRIP6, TRPM1, TST, TULP1, TWIST1, TYR, TYRP1, UGT8, UNC119B, VAMP3, VASN, VASP, VEGFD, VIT, VTN, WASF2, WDR38, WDR66, WEE1, WLS, WNT1, WNT2B, WNT5A, WNT8B, WTIP, YAP1, ZFP36L2, ZIC2*
**Controls**	**G11**	**animal organ morphogenesis**	1.60E-09	7	*ADAMTS1, ADAMTS18, ALDH1A3, ANKRD1, ARHGEF19, AXIN2, BAK1, BAX, BCAR3, BMP4, BSG, CCN2, CCNL1, CDK4, CDON, CFLAR, COL27A1, CTSZ, DMRT3, DNAAF1, DNAH11, ELF3, EPHA2, FGF18, FOXA1, FOXO1, FZD6, GCNT1, GDNF, GLI3, GPD2, GRHL3, HES1, ID1, ID3, IER3, ITGA6, KCP, LAMA5, LAMC3, LCTL, LGR5, LIF, LIM2, LMNB2, LRIG3, LZTS2, MAF, MERTK, MSX1, MSX2, MYLK, MYO3A, NEK8, NOG, NOS3, NPHP1, NPHP3, OSBPL1A, OTX1, PAX6, PAX7, PDE6B, PERP, PITX2, PLAG1, PMEL, PPP1R13L, PRKD2, RAX, RHBDF2, RPS6KA1, RSPO2, RSPO3, SBNO2, SERPINF1, SFRP2, SHH, SIX6, SLC9A3R1, SLIT2, SMAD3, SMAD6, SMO, SNAI1, STAT6, SULF1, TCIRG1, TEK, TGFBR3, TNNC1, TRPM1, TST, TULP1, TWIST1, VIT, WNT1, WNT2B, WNT5A, YAP1*
**Controls**	**G10**	**cell surface receptor signaling pathway**	1.10E-07	6	*AXIN2, BAX, BMP4, BSG, CTSZ, DMRT3, EPHA2, EPHA7, FOXA1, FOXO1, GDNF, GLI3, ID3, ITGA6, KCP, LAMA5, MSX1, MSX2, NOG, PERP, PITX2, PPP1CA, PRKD2, RSPO2, RSPO3, SFRP2, SHH, SLIT2, SMO, SULF1, TCIRG1, TNNC1, TWIST1, WNT1, WNT2B, WNT5A, YAP1*
**Controls**	**G09**	**cytoskeleton organization**	4.60E-09	4	*ADAMTS12, ADAMTS9, AGPAT2, ALDH1A3, ANKRD1, ANXA1, ARHGEF19, ASB2, AXIN2, B4GALT1, BAX, BMP4, BSG, CCN2, CCND1, CCNO, CDK4, CDON, CES1, CFLAR, CLDN1, COL27A1, CORIN, CTSZ, DNAAF1, DNPH1, DSG2, ECM1, ELF3, ENO3, EPHA2, EPHA7, EPPK1, F11R, FGF18, FLNA, FOXA1, FOXO1, FRMD6, FZD4, FZD6, GCNT1, GDNF, GLI3, GNPDA1, GORAB, GRHL3, HDAC7, HES1, ID1, ID3, IER3, ITGA6, KANK2, KCNN4, KCP, KLK6, KRT18, KRT8, LAMA5, LGR5, LIF, LMNB2, LZTS2, MAF, MAK, MCIDAS, MEOX1, MGMT, MSX1, MSX2, MYLK, NEBL, NOG, NOS3, NOX4, NPHP3, NRAP, NRTN, PAX6, PAX7, PERP, PGM5, PITX2, PKM, PMEL, PPL, PPP1CA, PPP1R13L, PRKD2, PRKDC, PRSS8, PTPRU, RBP1, RHBDF2, RREB1, RSPO2, RSPO3, S100A4, S1PR2, SBNO2, SFRP2, SHH, SLC25A37, SLC9A3R1, SLIT2, SMAD3, SMAD6, SMO, SNAI1, SP1, STAT6, SULF1, TBXAS1, TCF7L2, TCIRG1, TFAP2C, TGFBI, TGFBR3, TIE1, TNNC1, TST, TWIST1, VASN, VASP, VIT, VTN, WASF2, WLS, WNT1, WNT2B, WNT5A, YAP1*
**Controls**	**G08**	**developmental process involved in reproduction**	7.30E-05	2	*ABCA4, ACAA2, ACOX2, ALDH1A3, ANKRD1, ANXA1, APOE, ARHGEF17, ARHGEF19, AVEN, AXIN2, B4GALT1, BAK1, BARD1, BAX, BMF, BMP4, BSG, C5AR1, CAST, CCN2, CDK4, CFLAR, CTSZ, DLEU2, DOCK8, DUSP23, E2F1, EPHA7, FANK1, FAP, FLNA, FOSL1, FOXA1, FOXO1, GADD45A, GADD45B, GDF10, GDF15, GDNF, GLA, GLI3, GNPDA1, HSPA1B, ID1, ID3, IER3, IL1A, INPP5D, ITGA6, KANK2, KCP, KRT18, LATS2, MAL, MEOX1, MERTK, MGMT, MSX1, MSX2, NLRP2, NOD1, NOG, NOS3, NOX4, PAK4, PAX7, PERP, PKHD1, PMEL, PPP1CA, PRKDC, PRSS8, RASSF3, RAX, REST, RHBDF2, RPS6KA1, RTKN, S100A4, SBNO2, SDF2L1, SERPINF1, SFRP2, SHC1, SHH, SLC9A3R1, SLIT2, SMAD3, SMAD6, SMO, SNAI1, SPHK1, TCF7L2, TEK, TFAP2C, TFPI2, TMEM161A, TNFRSF10B, TNFRSF10C, TNFRSF10D, TP53I3, TRAF1, TRAF5, TWIST1, WNT1, WNT5A, YAP1*

**Controls**	**G07**	**extracellular structure organization**	3.40E-07	2	*ADAMTS18, ANXA1, APOA1, APOE, ARHGEF19, B4GALT1, BAX, BSG, CCN2, CERS2, CFLAR, CNN2, DOCK8, ENO3, EPPK1, ERBB2, F5, FAP, FLNA, FZD6, GLI3, GRHL3, ID3, IL1A, KANK1, KLK6, LMNB2, MERTK, MSX2, MTR, MYH9, MYL12A, MYLK, NOG, NOS3, PAPSS2, PAX6, PAX7, PITX2, PKM, PLSCR1, PPL, PTPN6, RAD51B, RHBDF2, RREB1, SERPINA5, SHH, SMAD3, TFAP2C, TFPI2, THBD, TNNC1, VTN, WASF2, WNT1, WNT5A, YAP1, ZFP36L2*
**Controls**	**G06**	**odontogenesis of dentin-containing tooth**	3.60E-07	2	*ANXA1, ANXA3, APOA1, ATIC, BAAT, BAK1, C5AR1, CCND1, CDK4, CERS2, CFLAR, CLDN1, ENO3, EPPK1, GLI3, KLK6, LMNB2, MTR, PAX7, PKM, PTPN3, PTPRU, RHBDF2, TBXAS1, TGFBR3, TNNC1, VTN, WNT1, YAP1*
**Controls**	**G05**	**positive regulation of biological process**	7.70E-05	2	*ABCA4, ACAA2, ADAMTS1, ADAMTS12, ADAMTS18, AFAP1L2, AFP, AGPAT2, ANKRD1, ANO1, ANXA1, ANXA3, APOA1, APOE, ARHGAP17, ARHGEF17, ARHGEF19, ASB2, ATP2A1, ATP6V1C2, AXIN2, BAK1, BARD1, BAX, BCAR3, BMF, BMP4, BSG, C1QTNF12, C5AR1, CAST, CCBE1, CCN2, CCND1, CD4, CDC14B, CDC42BPG, CDK4, CDON, CES1, CFLAR, CGN, CISH, CNN2, COL4A5, CROT, CXCL5, DDIT4L, DLEU2, DNMBP, DOCK8, DRD4, DUSP23, E2F1, ECM1, EFNA5, ELF3, EMP2, EPHA2, EPHA7, ERBB2, ESM1, F11R, FAM110C, FGF18, FLNA, FOXA1, FOXO1, FYB2, FZD4, FZD6, GABRE, GADD45A, GADD45B, GDF10, GDF15, GDNF, GLI3, GNPDA1, GORAB, GPBAR1, GPR87, GPRC5A, GRHL3, HDAC7, HEBP1, HES1, HHAT, HLA-DPB1, HRC, HSPA1B, ICAM3, ID1, IER3, IFITM1, IL1A, IL4R, INPP5D, IRS4, ISG20, ITGA6, ITGB1BP2, ITGB7, KANK1, KANK2, KCNN4, KCP, KLK6, KREMEN2, KRT18, KRT8, LAMA5, LATS2, LDLRAP1, LGR5, LIF, LMNB2, LPL, LTB4R, LZTS2, MAK, MAL, MAPK15, MEOX1, MERTK, MSX1, MSX2, MYH9, NECTIN2, NEDD9, NEK8, NFKB2, NLRP2, NLRX1, NOD1, NOG, NOP2, NOS3, NOX4, NPHP1, NPHP3, NR2F6, NRTN, NXN, OSBPL1A, OTUD7B, PABPN1, PAK4, PAMR1, PARP4, PAX6, PDE6B, PDE7A, PERP, PITX2, PKHD1, PLEKHA4, PLEKHG6, PLSCR1, PMEL, POLR2H, POMC, PPP1CA, PPP1R13L, PRKD2, PRKDC, PRSS33, PRSS8, PTBP1, PTPN3, PTPN6, PTPRU, RAB11FIP1, RAB32, RASSF3, RAX, RCC2, RGL3, RHBDF1, RHBDF2, RHOH, RIN1, RPS6KA1, RREB1, RSPO2, RSPO3, RTKN, S100A4, S100A6, S1PR2, SAMD11, SBNO2, SCUBE3, SFRP2, SHC1, SHH, SHKBP1, SIVA1, SLC39A4, SLC44A2, SLC7A8, SLC9A3R1, SLIT2, SMAD3, SMAD6, SMO, SMYD2, SNAI1, SP1, SPHK1, STAT6, STK33, STOML2, STX3, SULF1, SULT1A1, SYDE1, SYDE2, TAF4B, TBC1D10C, TCF7L2, TCIRG1, TCTN2, TEAD3, TEK, TFAP2C, TGFBR3, THNSL2, TIE1, TKFC, TMEM161A, TMEM88, TNFRSF10B, TNFRSF10C, TNFRSF10D, TNNC1, TOM1L1, TPCN1, TRABD2B, TRAF1, TRAF5, TRH, TRIM6, TRIP6, TRPM1, TRPM3, TSPAN4, TST, TWIST1, UNC93B1, VASN, VEGFD, VTN, WASF2, WLS, WNT1, WNT2B, WNT5A, WNT8B, WTIP, YAP1, ZFP36L2*
**Controls**	**G04**	**regeneration**	8.30E-05	1	*AXIN2, BMP4, GDF10, GLI3, HDAC7, ID1, ID3, IFITM1, MSX2, NOG, OPTC, REST, SFRP2, SMAD3, SMAD6, TCIRG1, TWIST1*
**Controls**	**G03**	**regulation of apoptotic process**	3.20E-05	1	*ADAMTS9, APOA1, APOE, B4GALT1, BSG, CCN2, CES1, CFLAR, COL24A1, COL27A1, COL4A5, COLGALT1, ELF3, F11R, FAP, HSPG2, ICAM3, ITGA6, ITGB7, LAMA5, LAMC3, LMNB2, LPL, MEOX1, NFKB2, OLFML2B, SCUBE3, SFRP2, SMAD3, SULF1, TGFBI, TNNC1, TWIST1, TYRP1, VIT, VTN*
**Controls**	**G02**	**regulation of osteoblast differentiation**	3.80E-05	1	*ADAMTS1, AFP, ANXA1, B4GALT1, BAK1, BAX, BMP4, BSG, C14orf39, CCND1, CENPI, CFAP157, CFTR, CTDSPL, DMRT3, FLNA, FOSL1, FOXA1, FZD4, GLI3, HES1, HSPA2, IL1A, KRT8, LGR5, LIF, MERTK, NECTIN2, NOG, NOS3, NPHP1, PITX2, PLAG1, PRKD2, PRKDC, RBP1, RFX2, RSPO3, SERPINA5, SERPINF1, SFRP2, SHH, SLC26A6, SLIT2, SNAI1, SPAG6, SULF1, TAF4B, TFAP2C, TNNC1, TYRP1, WNT2B, WNT5A*

**Controls**	**G01**	**response to wounding**	5.30E-05	1	*ACOX2, ADAMTS1, ADAMTS9, AFAP1L2, AGPAT2, ALDH1A3, ANKRD1, ANO1, ANXA1, ANXA3, APOA1, APOE, ARHGEF17, ARHGEF19, ATP2A1, ATP6V1C2, AXIN2, B4GALT1, BAK1, BARD1, BAX, BCAR3, BMF, BMP4, C1QTNF12, C1RL, C5AR1, CAST, CASZ1, CCBE1, CCN2, CCND1, CCNJ, CCNO, CD4, CDC14A, CDC14B, CDC20B, CDK4, CDON, CES1, CFAP69, CFLAR, CFTR, CHD1L, CHMP4C, CIP2A, CISH, CLDN1, CNN2, COLGALT1, CROT, CTSZ, CXCL5, DDB2, DLEU2, DNPH1, DOCK8, DRD4, E2F1, ECM1, EFNA5, ELF3, EMILIN2, EMP2, EPHA2, EPHA7, EPHX2, ERBB2, ESM1, FAM110C, FANK1, FAP, FES, FEZF1, FGF18, FGFR1OP, FLNA, FOSL1, FOXA1, FOXO1, FYB2, FZD4, GADD45A, GADD45B, GAL3ST1, GDF10, GDF15, GDNF, GLA, GLI3, GNPDA1, GORAB, GPRC5A, GRHL3, H1F0, HDAC7, HES1, HIST1H1C, HLA-DPB1, HMGN3, HOXB9, HRC, HSPA1B, HSPA2, HSPB8, ICAM3, ID1, ID3, IFITM1, IL1A, IL4R, INPP5D, IRS4, ITGA6, KANK1, KANK2, KCNN4, KCP, KLK6, LATS2, LDLRAP1, LGR5, LIF, LMNB2, LMOD1, LMX1A, LPL, MAF, MAK, MAL, MAPK15, MCIDAS, MEOX1, MERTK, MFGE8, MGMT, MSX1, MSX2, MYH9, MYLK, NECTIN2, NEDD9, NFKB2, NLRP2, NLRX1, NOD1, NOG, NOP2, NOS3, NOX4, NPHP1, OPTC, OSBPL1A, OTX1, PACSIN3, PAK4, PAMR1, PARP4, PAX6, PAX7, PBX2, PERP, PITX2, PKHD1, PKM, PLAG1, PLEKHA4, PLSCR1, POLR1C, POLR2H, POMC, PPP1CA, PPP1R13L, PRKD2, PRKDC, PRSS8, PTBP1, PTPN6, PTPRU, RAB11FIP1, RAD51B, RASSF3, RAX, RBP1, RCC2, REST, RFX2, RHBDF2, RPS6KA1, RREB1, RSPO2, RSPO3, S100A4, S100A6, S1PR2, SBNO2, SCUBE3, SERPINF1, SERTAD1, SERTAD3, SFRP2, SH3D19, SHC1, SHH, SHKBP1, SIX6, SLC24A1, SLC26A6, SLC44A2, SLC4A11, SLC50A1, SLC7A8, SLC9A3R1, SLIT2, SMAD3, SMAD6, SMARCD2, SMO, SMTN, SNAI1, SP1, SPHK1, SPTLC3, STAT6, STN1, STOML2, STON1, STX3, STXBP2, SULF1, SYDE1, TAF4B, TBXAS1, TCF7L2, TCIRG1, TEAD3, TEK, TFAP2C, TFEC, TFPI2, TGFBR3, THBD, TIE1, TKFC, TMEM161A, TMEM98, TNFRSF10B, TNFRSF10C, TNNC1, TOM1L1, TPBG, TPCN1, TPPP3, TRABD2B, TRAF1, TRAF5, TRH, TRIM11, TRIM6, TRIP6, TRMT112, TRPV3, TSC22D4, TULP1, TWIST1, TYRP1, UNC93B1, VAMP3, VASP, VEGFD, VIT, VTN, WASF2, WLS, WNT1, WNT2B, WNT5A, WTIP, YAP1, ZFP36L2, ZIC2*
**Controls**	**G00**	**tissue development**	4.50E-06	1	*ANKRD1, ANXA1, APOA1, APOE, ARHGAP17, ARHGEF17, ARHGEF19, CCDC114, CCDC8, CCN2, CDC14A, CDC14B, CDC42BPG, CFAP157, CFAP74, CFLAR, CHMP4C, CLMN, CNN2, DIAPH3, DNAAF1, DNAAF5, DNAI2, EFNA5, EMP2, EPPK1, F11R, FES, FGFR1OP, FLII, FLNA, FRMD6, GADD45A, GRHL3, HSPA1B, ID1, INF2, KANK1, KANK2, KIF19, KRT18, KRT8, LAMA5, LIF, LMOD1, LZTS2, MAL, MAPK15, MCIDAS, MYH9, MYLK, NEBL, NECTIN2, NEDD9, NOX4, NPHP1, NRAP, OSBPL1A, PACSIN3, PAK4, PARD6G, PARP4, PAX6, PDLIM4, PGM5, PIH1D3, PKHD1, PLS3, PMEL, PPL, PRKD2, PRSS8, RHOH, RTKN, RTTN, S1PR2, SH3D19, SHC1, SHH, SHROOM4, SLC9A3R1, SLIT2, SMAD3, SMTN, SPAG17, SULT1A1, SYDE1, TEK, TESK2, TPPP3, TUBB6, UGT8, VASP, WASF2, WEE1, WTIP*
**RSTS**	**G07**	**RNA processing**	1.10E-12	42	*AATF, ANP32A, APOBEC3C, CPAMD8, CPSF3, DCTPP1, DKC1, EBNA1BP2, EIF2S3, ENY2, FBL, GAPDH, GAR1, GNL2, HNRNPA1, HNRNPA2B1, HNRNPD, HNRNPH1, HPRT1, LSM6, LY6E, LYAR, MAGOHB, MBNL3, METTL1, MXD3, NDC1, NIP7, NOB1, NOP16, NOP58, NUDT1, NUDT5, NUP35, NUTF2, PA2G4, PABPC1, PABPC1L, PBK, PNN, POLR3K, PPIA, PRKCD, PUS7, RBMX, RPF2, RPL12, RPL23A, RPL35, RPL41, RPL7A, RPL8, RPLP1, RPS26, RPS3, RPS6, RPS7, RSL1D1, RUVBL1, SEM1, SLIRP, SNAPC1, SNRNP40, SNRNP48, SNRPD1, SNRPD2, SNRPD3, SNRPF, SNRPG, SRSF3, SRSF7, SUV39H1, TERT, TMCO6, TNFRSF1B, TTK, UNG, ZBTB8OS, ZC3HAV1*
**RSTS**	**G06**	**DNA metabolic process**	1.90E-08	17	*ABRAXAS1, APOBEC3C, BAZ1A, CDC7, CDK1, CHTF18, DAXX, DBF4B, DCTPP1, DKC1, DNAJC2, FANCB, FANCG, GAR1, GNL3, HNRNPA1, HNRNPA2B1, HNRNPD, MDC1, MSH6, NAP1L1, NME1, NSMCE4A, NUDT1, PBK, PHB, PMS2P1, POLA1, POLD2, PPIA, PRIM1, PRIM2, PRKCD, REC8, RECQL4, RPA2, RPS3, RUVBL1, SEM1, SFR1, SSBP1, SSRP1, TERT, TIPIN, UNG, ZNF367*
**RSTS**	**G05**	**purine ribonucleoside monophosphate biosynthetic process**	2.40E-05	13	*CTPS1, DCTPP1, GAPDH, GMPS, HPRT1, IMPDH2, MTAP, MTHFD1, NDC1, NME1, NPPB, NUDT1, NUDT5, NUP35, PAICS, PFAS, PPAT, PPCDC, PRPS2, SEM1, UNG*
**RSTS**	**G04**	**ribonucleoprotein complex biogenesis**	2.60E-12	11	*AATF, ANP32A, CPSF3, DKC1, EBNA1BP2, EIF2S3, ENY2, FBL, GAR1, GNL2, HNRNPA1, HNRNPA2B1, LSM6, LY6E, LYAR, MAGOHB, MXD3, NDC1, NIP7, NOB1, NOP16, NOP58, NUP35, NUTF2, PA2G4, PBK, PRKCD, RPF2, RPL12, RPL23A, RPL35, RPL7A, RPS6, RPS7, RSL1D1, RUVBL1, SEM1, SNRPD1, SNRPD2, SNRPD3, SNRPF, SNRPG, SRSF3, SRSF7, SUV39H1, TMCO6, TTK*
**RSTS**	**G03**	**ribosome biogenesis**	1.70E-08	6	*AATF, DKC1, EBNA1BP2, FBL, GAR1, GNL2, LSM6, LYAR, NIP7, NOB1, NOP16, NOP58, PA2G4, RPF2, RPL12, RPL23A, RPL35, RPL7A, RPS6, RPS7, RSL1D1, SUV39H1, TTK*
**RSTS**	**G02**	**telomere maintenance**	5.10E-06	6	*CHTF18, DKC1, DNAJC2, GAR1, GNL3, HNRNPA1, HNRNPA2B1, HNRNPD, NME1, PBK, PHB, POLA1, POLD2, PPIA, PRIM1, PRIM2, RECQL4, RPA2, TERT*
**RSTS**	**G01**	**cellular response to antibiotic**	1.10E-03	4	*CDK1, CSRP1, DAXX, FXN, HNRNPA1, MYB, NET1, PBK, PPIF, PRDX4, PRKCD, RACK1, RPS3, TXNDC17*
**RSTS**	**G00**	**positive regulation of helicase activity**	6.50E-04	2	*MSH6, RPA2, SSBP1*
